# Genomic diversity and signatures of selection in meat and fancy rabbit breeds based on high-density marker data

**DOI:** 10.1186/s12711-022-00696-9

**Published:** 2022-01-21

**Authors:** Mohamad Ballan, Samuele Bovo, Giuseppina Schiavo, Michele Schiavitto, Riccardo Negrini, Luca Fontanesi

**Affiliations:** 1 Division of Animal Sciences, Department of Agricultural and Food Sciences, University of Bologna, Viale Giuseppe Fanin 46, 40127 Bologna, Italy; 2 Associazione Nazionale Coniglicoltori Italiani (ANCI), Contrada Giancola Snc, 71030 Volturara Appula, FG Italy; 3 Associazione Italiana Allevatori, Via G. Tomassetti 9, 00161 Rome, Italy

## Abstract

**Background:**

Domestication of the rabbit (*Oryctolagus cuniculus*) has led to a multi-purpose species that includes many breeds and lines with a broad phenotypic diversity, mainly for external traits (e.g. coat colours and patterns, fur structure, and morphometric traits) that are valued by fancy rabbit breeders. As a consequence of this human-driven selection, distinct signatures are expected to be present in the rabbit genome, defined as signatures of selection or selective sweeps. Here, we investigated the genome of three Italian commercial meat rabbit breeds (Italian Silver, Italian Spotted and Italian White) and 12 fancy rabbit breeds (Belgian Hare, Burgundy Fawn, Champagne d’Argent, Checkered Giant, Coloured Dwarf, Dwarf Lop, Ermine, Giant Grey, Giant White, Rex, Rhinelander and Thuringian) by using high-density single nucleotide polymorphism data. Signatures of selection were identified based on the fixation index (F_ST_) statistic with different approaches, including single-breed and group-based methods, the latter comparing breeds that are grouped based on external traits (different coat colours and body sizes) and types (i.e. meat vs. fancy breeds).

**Results:**

We identified 309 genomic regions that contained signatures of selection and that included genes that are known to affect coat colour (*ASIP*, *MC1R* and *TYR*), coat structure (*LIPH*), and body size (*LCORL*/*NCAPG*, *COL11A1* and *HOXD*) in rabbits and that characterize the investigated breeds. Their identification proves the suitability of the applied methodologies for capturing recent selection events. Other regions included novel candidate genes that might contribute to the phenotypic variation among the analyzed breeds, including genes for pigmentation-related traits (*EDNRA*, *EDNRB*, *MITF* and *OCA2*) and body size, with a strong candidate for dwarfism in rabbit (*COL2A1*).

**Conclusions:**

We report a genome-wide view of genetic loci that underlie the main phenotypic differences in the analyzed rabbit breeds, which can be useful to understand the shift from the domestication process to the development of breeds in *O. cuniculus*. These results enhance our knowledge about the major genetic loci involved in rabbit external traits and add novel information to understand the complexity of the genetic architecture underlying body size in mammals.

**Supplementary Information:**

The online version contains supplementary material available at 10.1186/s12711-022-00696-9.

## Background

The European rabbit (*Oryctolagus cuniculus*), usually simply referred to as rabbit, is the only species that has been domesticated exclusively in western Europe. Its domestication started from the wild populations in the South of France that originally derived from the wild populations of the *O. c. cuniculus* subspecies spread in the Iberian Peninsula, which experienced a postglacial expansion (reviewed in [1]). Among the possible animal domestication trajectories that have been theorised [2], domestication of the rabbit better matches the directed pathway that does not involve preliminary steps of habituation of animals to human beings and begins with the capture of wild animals, with the aim of controling their breeding and reproduction. It seems plausible that this process occurred quite recently in rabbit, starting in the French monasteries and castles in the High Middle Ages and, continuing until the XV–XVI centuries [3, 4], and then may have continued through the dispersion and transfer of rabbits in the North of Europe, until the most recent constitution of some modern breeds [5]. Domestication of the rabbit occurred after a first genetic bottleneck that involved the wild subpopulations from which the domestic lines were then derived, accompanied by limited recurrent introgression from the wild types [6, 7]. However, this resulted in only slightly modified allele frequencies at many loci between the wild and domestic rabbit populations, which suggests that the domestication process had a relatively weak effect on standing genetic variation in many regulatory regions of the genome [6]. These changes occurred mainly in genes that are involved in brain and neuronal development, which indicates that the resulting modified behaviour traits were important for the domestic rabbit to adapt to the human environment. Thus, in this species, the domestication process relied on derived genetic material, which contained variants that determine favourable behavioral traits and facilitate handling and breeding [6].

The domestication process was integrated or was followed by artificial selection processes that led to the constitution of many breeds. The resulting rabbit breeds can be distinguished based on their broad phenotypic diversity, in terms of external traits that are valued by fancy breeders [1, 8, 9]. It is also worth mentioning that the most recently constituted rabbit lines or strains and the modern breeds have been derived by cross-breeding pre-existing varieties or morphs, with the aim to introgress desirable traits to create new lines or combinations of morphological features or to improve production traits in specialized meat lines [1, 8, 9]. However, coat colours and coat colour patterns are the most relevant traits that differentiate many rabbit breeds, as also demonstrated by the fact that many breeds are named according to their colouration [1, 9].

In rabbits as well as in many other mammals, several coat colour loci were first described by classical genetic studies that confirmed the Mendelian segregation of these inherited traits and established homology across species [10]. Subsequent molecular characterizations identified the causal mutations or associated markers at some of these loci. At the *extension* locus, three mutated alleles (*E*^*D*^ or *E*^*S*^, determining the dominant black/steel coat colour; *e*^*J*^, determining the Japanese brindling pattern identified in the Japanese and Rhinelander breeds [12]; *e*, determining the recessive yellow/red coat colour) are caused by mutations in the *melanocortin 1 receptor* (*MC1R*) gene [11, 12]. The *agouti* locus is determined by mutations in the *agouti signaling protein* (*ASIP*) gene that form the recessive black non-agouti (*a*) and tan (*a*^*t*^) alleles [13, 14]. Several alleles at the *albino* locus (*C* series) are caused by mutations in the *tyrosinase* (*TYR*) gene that produce the chinchilla, the Himalayan (of the Californian breed), and the full albino coat colours (of the New Zealand White and related breeds and populations) [15, 16]. At the *English spotting* locus, a marker in the *v-kit Hardy-Zuckerman 4 feline sarcoma viral oncogene homolog* (*KIT*) gene is associated with the spotted pattern of the Checkered Giant and Rhinelander breeds, whose classical spotted design is due to the heterozygous genotype *En/en* that preserves these animals from a megacolon defect associated with the *En* allele [17]. Other coat colour loci might be involved in the spotted phenotypes of rabbits but, to date, the corresponding genes have not been identified [1, 10].

In addition to coat colour, breeds can be distinguished by their hair structure. For example, a mutation in the *lipase member H* (*LIPH*) gene determines the *Rex* locus *R*^*1*^ [18] that confers soft down-hair. The shape and position of the ears are other morphological traits that differentiate some breeds (e.g. several lop breeds). Another main morphological feature of rabbit breeds is body size: breeds are traditionally classified into dwarf, small, medium, and large classes according to their adult body weight [9]. Carneiro et al. [19] identified a large deletion in the *high mobility group AT-hook 2* (*HMGA2*) gene as the causal mutation for one type of dwarfism in rabbit. However, other loci might also contribute to the reduced size of some dwarf rabbits and several loci involved in dwarfism have been reported in this species (reviewed in [20]).

Although several studies have successfully started to dissect the genetic mechanisms underlying some external traits in rabbit, these have mainly focused on a few candidate genes, and a complete genetic picture of the phenotypic diversity of many rabbit breeds is still lacking, not only in terms of coat colour but also in terms of body size, meat production and performance traits. Few studies in this species have been designed to investigate the variability at the genome-wide level and identify footprints of recent selection that distinguish rabbit breeds [6, 19, 21].

In the current study, we used high-density single nucleotide polymorphism (SNP) genotyping data from three commercial meat breeds and 12 fancy breeds that differ for several external traits to evaluate the level of genetic diversity and identify signatures of selection in the rabbit genome that may explain the phenotypic variability that differentiates these breeds.

## Methods

### Animals

Biological specimens (hair roots or buccal swaps) were collected from 660 rabbits from 15 breeds, including three commercial meat lines (Italian Silver, Italian Spotted and Italian White) and 12 fancy breeds (Belgian Hare, Burgundy Fawn, Champagne d’Argent, Checkered Giant, Coloured Dwarf, Dwarf Lop, Ermine, Giant Grey, Giant White, Rex, Rhinelander and Thuringian). All rabbits had the standard breed characteristics, as registered in the corresponding breed herd book maintained by the Italian Rabbit Breeders Association (ANCI). The description of the breeds and the number of animals analysed for each breed are in Table 1. Sampled rabbits were chosen to avoid highly related individuals (no full- or half-sibs).

**Table 1:** Details on the analysed breeds and animals

Breed	Acronym	Number of animals	Coat colour	Body size	Other information and characteristics
Meat breeds					
Italian Silver	ISI	20	Non-agouti black at birth and then silver	Medium	Derived from Champagne d’Argent
Italian Spotted	ISP	93	Himalayan coat colour pattern (white albino with black spots at the extremities)	Medium	Derived from Californian
Italian White	IW	256	White albino	Medium	Derived from New Zealand White
Fancy breeds					
Belgian Hare	BH	24	Brownish/dark red	Medium	
Burgundy Fawn	BF	6	Red/fawn	Medium	Genotype *e*/*e* at the *Extension* locus
Champagne d’Argent	CdA	19	Non-agouti black at birth and then silver	Medium	
Checkered Giant	CG	79	White with black spots	Giant	Heterozygous at the English spotted locus (*En/en* genotype)
Coloured Dwarf	CD	20	Various colours	Dwarf	
Dwarf Lop	DL	20	Various colours	Dwarf	Lopped ears
Ermine	ER	20	Greysh/cream	Small	
Giant Grey	GG	27	Grey/wild type	Large	
Giant White	GW	20	White albino	Large	
Rex	RE	19	Various colours	Medium	
Rhinelander	RH	28	Tricolour (white with black and red spots)	Medium	Heterozygous at the English spotted locus (*En*/*en* genotype). Genotype *e*^*J*^/*e*^*J*^ at the *Extension* locus
Thuringian	TH	9	Pale red with dark shades	Medium	Genotype *e*/*e* at the *Extension* locus

### Genotyping

DNA was extracted using the Wizard Genomic DNA Purification kit (Promega Corporation, Madison, WI, USA). Animals were then genotyped using the Affymetrix Axiom OrcunSNP array (Affymetrix Inc., Santa Clara, CA, USA), which can analyse 199,692 SNPs. Low-quality SNPs were removed using the Axiom Analysis Suite and the PLINK v.1.9 software [22]. After filtering, 660 samples that had a call rate higher than 0.90 were retained and 139,922 SNPs remained for the subsequent analyses. Minor allele frequency within breeds and across breeds was not used to filter SNPs to avoid potential biases derived by the different number of animals analysed per breed.

### Genetic diversity and population genomic parameters

Observed (H_o_) and expected (H_e_) heterozygosity and fixation index (F_ST_) were calculated with the PLINK v.1.9 software [22]. F_ST_ values were based on the Hudson estimator, as this index is independent of sample size [23]. The inbreeding coefficient of an individual (I) relative to the subpopulation (S) (F_IS_) was calculated as F_IS_ = 1 − H_o_/H_e_, [24]. The genetic distance between pairs of breeds was estimated as the average F_ST_ value across all SNPs [25, 26]. The resulting averaged F_ST_ values were used to build an F_ST_ matrix of size 15 × 15 that was then used to build a Neighbour-Joining (NJ) tree with the function “nj” in R v.4.0.4 based on 10,000 bootstrap replicates. Genetic differences between the 15 breeds were also evaluated using the genotyped SNPs with a multidimensional scaling (MDS) analysis, as implemented in PLINK v.1.9. Linkage disequilibrium (LD) was measured using *r*^2^ for all SNP pairs on each chromosome using PLINK v.1.9 [22]. In addition, LD decay was estimated in bins of 50 kb to compare differences between and within breeds. Effective population size (N_e_) at recent generations was computed using SNP data with the SNeP v.1.1 software based on default parameters [27]. Plots were generated in R v.4.0.4 [28].

### Exploratory analysis of markers under selection

An exploratory analysis to detect outlier markers that could be under selection was performed using the *PCAdapt* package in R [29]. This analysis does not require prior knowledge on population structure and was performed on the merged dataset of the 15 rabbit breeds that consisted of 139,922 SNPs. Briefly, *PCAdapt* applies a principal component analysis (PCA) that selects the components (*K*) that explain the greatest amount of variation, followed by a statistical test used to detect outliers SNPs. As suggested by [30], the number of principal components *K* to work with was selected based on a Scree plot. For the identification of outliers SNPs, *PCAdapt* calculated a vector of *z*-scores *z*_*j*_ = (*z*_*j*1_,…, *z*_*jK*_) obtained by regressing each *j*-th SNP by the *k*-th principal components via a multiple linear regression model [29]. Then, the Mahalanobis distance ($D$) statistical test was computed to detect ouliers SNPs as follows:

$${D}_{j}^{2}=({{z}_{j}}-\overline{\mathbf{z}}{)}^{T}{{\varvec{\Sigma}}}^{-1}\left({{z}_{j}}-\overline{\mathbf{z}}\right),$$

where ${\varvec{\Sigma}}$ is the ($K\times K)$ covariance matrix of the *z*-scores and $\overline{z}$ is the vector of the $K$ *z*-score means [31]. Mahalanobis distances were successively transformed into *P*-values [29]. The threshold to identify outlier SNPs was defined based on a Bonferroni corrected *P*-value of 0.1.

### Detection of signatures of selection

The F_ST_ analysis was further exploited to detect signatures of selection in the analysed rabbit breeds. F_ST_ single-marker-based analysis was performed by considering the markers at the extreme lower end of the distributions (99.95th percentile of distribution). Since the OryCun2.0 rabbit genome assembly is not well defined (the N50 length for the contigs is 64,648 bp), many genomic regions are probably misplaced in the current assembly. Therefore, identification of signatures of selection relied mainly on window-based analyses that could reduce the misassembly bias caused by small contigs.

F_ST_ was computed in 350-kb sliding genomic windows, with a step size of 100 kb. The choice of the best window size followed the method proposed by Rubin et al. [32]. Briefly, windows of variable sizes (from 50 to 500 kb) were evaluated for the number of windows with less than three SNPs. Window counts decreased asymptotically and stabilized after the 350-kb threshold (see Additional file 1: Table S1), resulting in 7951 genomic windows, with on average 17.5 ± 7 SNPs. Windows with less than three SNPs were not considered in the analyses. F_ST_ values were averaged across the SNPs in each genomic window. Two approaches were then applied to identify signatures of selection: (i) a single-breed approach to capture breed-specific features that could characterize each breed; and (ii) an approach based on groups of breeds to capture common features of several breeds.

For the first approach (single-breed approach), two methods were used to calculate window-based F_ST_ values:

i. in Method 1 (M1), for each breed, the F_ST_ value of SNPs was computed by comparing the given breed against all rabbits of the remaining *N* − 1 breeds (*N* = 15), considered as a unique population [7] and averaged across all SNPs within a genomic window;
ii. in Method 2 (M2), *N* − 1 F_ST_ pairwise comparisons were performed for each breed by comparing the breed against one of the remaining *N* − 1 breeds. The F_ST_ value of each genomic window for each comparison was computed as in M1 and then averaged across the *N* − 1 F_ST_ pairwise comparisons for that window and breed [26, 33, 34].

In the second approach, groups of breeds were defined and contrasted in pre-defined pairwise comparisons. Groups of breeds were defined according to the following common features: (i) coat colours, (ii) coat colour patterns, (iii) body size, and (iv) commercial meat lines vs fancy breeds. Details of the groups of breeds and of the group-based F_ST_ pairwise analyses are summarised in Additional file 1: Table S2.

Following Rubin et al. [19], we considered two stringency levels to identify the signatures of selection. Based on the most stringent level, signatures of selection were identified from genomic windows at the 99.8th percentile of the distribution of the F_ST_ values. Suggestive signatures of selection were detected using a less stringent threshold that considered the 99.0th percentile of the distribution.

To facilitate identification of candidate genes from the window-based approaches, considering potential assembly problems of the OryCun2.0 genome version (contig N50 = 64.648 kb), which could affect the precise genome position of annotated relevant genes, identified windows were further expanded by 200 kb at each side, i.e. about three times the contig N50 at each side [26, 35, 36]. Genomic regions were retrieved from the expanded windows for annotation and identification of overlaps between the two methods (M1 and M2) for each breed and combining results obtained for all breeds. Data were graphically represented via Manhattan plots. Pipelines were developed either in Python or in R (“manhattan” function of the “qqman” library; [37]).

Genomic regions that displayed signatures of selection or suggestive signatures of selection were annotated with the Bedtools v.2.17 (https://bedtools.readthedocs.io/ [38]) by retrieving annotated protein coding genes from the OryCun2.0 NCBI’s GFF file. The functional relevance of the genes annotated in the identified regions was evaluated based on a detailed analysis of the scientific literature, using the Gene Cards information [39] and the GWAS catalogue resource [40]. Over-representation analysis across sets of human traits was carried out for breed groups with Enrichr [41] via Fisher’s exact test. The following libraries were interrogated: (i) the Gene Ontology (GO) Biological Process (PB) branch, (ii) the Kyoto Encyclopedia of Genes and Genomes (KEGG; Human), (iii) WikiPathways (Human), (iv) the MGI Mammalian Phenotype (Level 4) and (v) the GWAS catalogue. For each over-representation analysis, genes located in the 99.8th percentile of genome windows were used as input set. Statistically enriched terms were defined if (i) they included at least two genes of the input set related to two or more of the top windows and (ii) had an adjusted *P*-value for enrichment less than 0.05.

## Results

### Within-breed genomic parameters

**Fig. 1:**
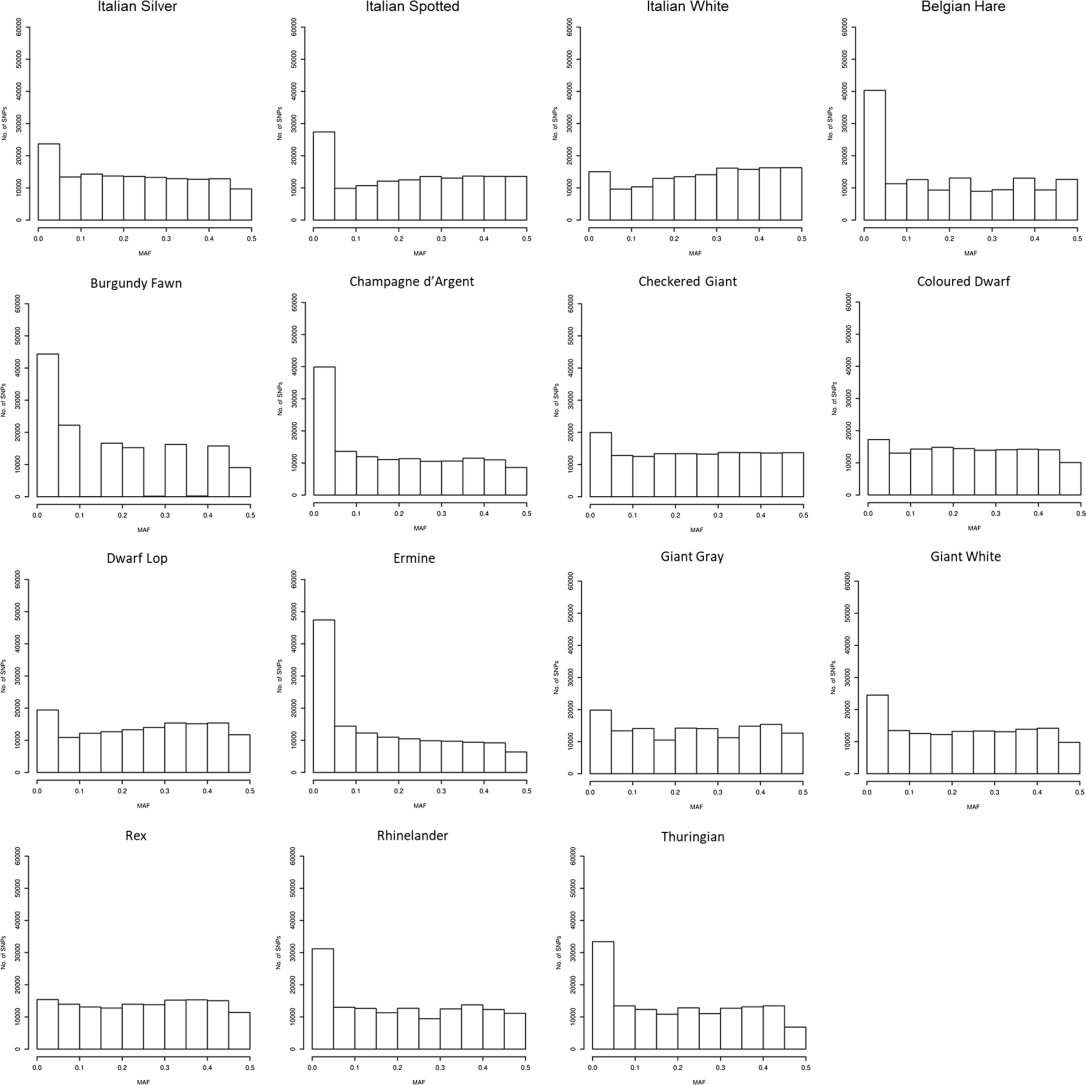
Distribution of minor allele frequencies (MAF)

In total, 660 rabbits from 15 breeds that are characterized by different external features or purposes (coat colour and pattern, body size, fur type, and selected for meat production or for exhibitions/shows) were genotyped. Table 2 summarises some basic population genomic parameters for the analysed breeds. The average within-breed minor allele frequency (MAF) ± s.d. ranged from 0.178 ± 0.160 in the Ermine breed (a rare fancy breed) to 0.266 ± 0.152 in the Italian White breed (a selected meat line). Accordingly, the Ermine breed had the largest number of SNPs (N = 47,409) with MAF lower than 0.05 and the Italian White breed had the largest number of informative SNPs (MAF ≥ 0.45) (N = 16,307). See also the MAF distributions in Fig. 1. Observed (H_o_) and expected (H_e_) heterozygosities ranged from 0.278 (Ermine) to 0.407 (Burgundy Fawn) and from 0.307 (Ermine) to 0.360 (Dwarf Lop), respectively. The value of the F_IS_ parameter was positive in four breeds (Coloured Dwarf, Dwarf Lop, Ermine and Rex), indicating non random mating in these breeds.

**Table 2:** Overview of the investigated rabbit breeds and related population parameters

Breed	MAF (sd)	H_o_ (sd)	H_e_ (sd)	F_IS_ (sd)	M1-F_ST_ (sd)	M2-F_ST_ (sd)	N_e_
Meat breeds							
Italian Silver	0.23 (0.15)	0.35 (0.19)	0.34 (0.16)	− 0.03	0.14 (0.08)	0.23 (0.22)	35
Italian Spotted	0.23 (0.15)	0.34 (0.18)	0.33 (0.17)	− 0.01	0.12 (0.08)	0.23 (0.22)	43
Italian White	0.27 (0.15)	0.36 (0.16)	0.36 (0.15)	− 0.01	0.09 (0.07)	0.21 (0.20)	94
Fancy breeds							
Belgian Hare	0.20 (0.16)	0.34 (0.22)	0.32 (0.19)	− 0.06	0.20 (0.13)	0.28 (0.26)	46
Burgundy Fawn	0.18 (0.16)	0.41 (0.26)	0.35 (0.19)	− 0.15	0.23 (0.13)	0.29 (0.27)	17
Champagne d’Argent	0.19 (0.17)	0.33 (0.21)	0.32 (0.19)	− 0.01	0.21 (0.13)	0.27 (0.26)	34
Checkered Giant	0.24 (0.16)	0.34 (0.17)	0.33 (0.16)	− 0.01	0.13 (0.09)	0.22 (0.21)	86
Coloured Dwarf	0.25 (0.17)	0.29 (0.16)	0.35 (0.15)	0.17	0.14 (0.09)	0.22 (0.21)	54
Dwarf Lop	0.26 (0.16)	0.31 (0.17)	0.36 (0.16)	0.12	0.14 (0.09)	0.22 (0.21)	50
Ermine	0.18 (0.16)	0.28 (0.19)	0.31 (0.18)	0.09	0.23 (0.13)	0.28 (0.27)	33
Giant Grey	0.24 (0.15)	0.35 (0.19)	0.35 (0.16)	− 0.01	0.13 (0.09)	0.22 (0.21)	63
Giant White	0.24 (0.16)	0.35 (0.20)	0.34 (0.17)	− 0.02	0.14 (0.09)	0.22 (0.21)	43
Rex	0.25 (0.15)	0.32 (0.19)	0.35 (0.16)	0.08	0.14 (0.09)	0.22 (0.21)	40
Rhinelander	0.22 (0.17)	0.35 (0.21)	0.33 (0.18)	− 0.05	0.17 (0.11)	0.26 (0.24)	42
Thuringian	0.21 (0.16)	0.40 (0.25)	0.35 (0.19)	− 0.13	0.20 (0.12)	0.26 (0.25)	24

MAF: average minor allele frequency (standard deviation); H_o_: observed Heterozygosity (standard deviation); H_e_: expected Heterozygosity (standard deviation); F_IS_: inbreeding coefficient individual (I) relative to the subpopulation (S), (sd: standard deviation); A M1-F_ST_: average F_ST_ value computed by using Method 1 (standard deviation); A M2-F_ST_: average F_ST_ value computed by using Method 2 (standard deviation); N_e_: effective population size at the most recent generation (generation 13)

**Fig. 2:**
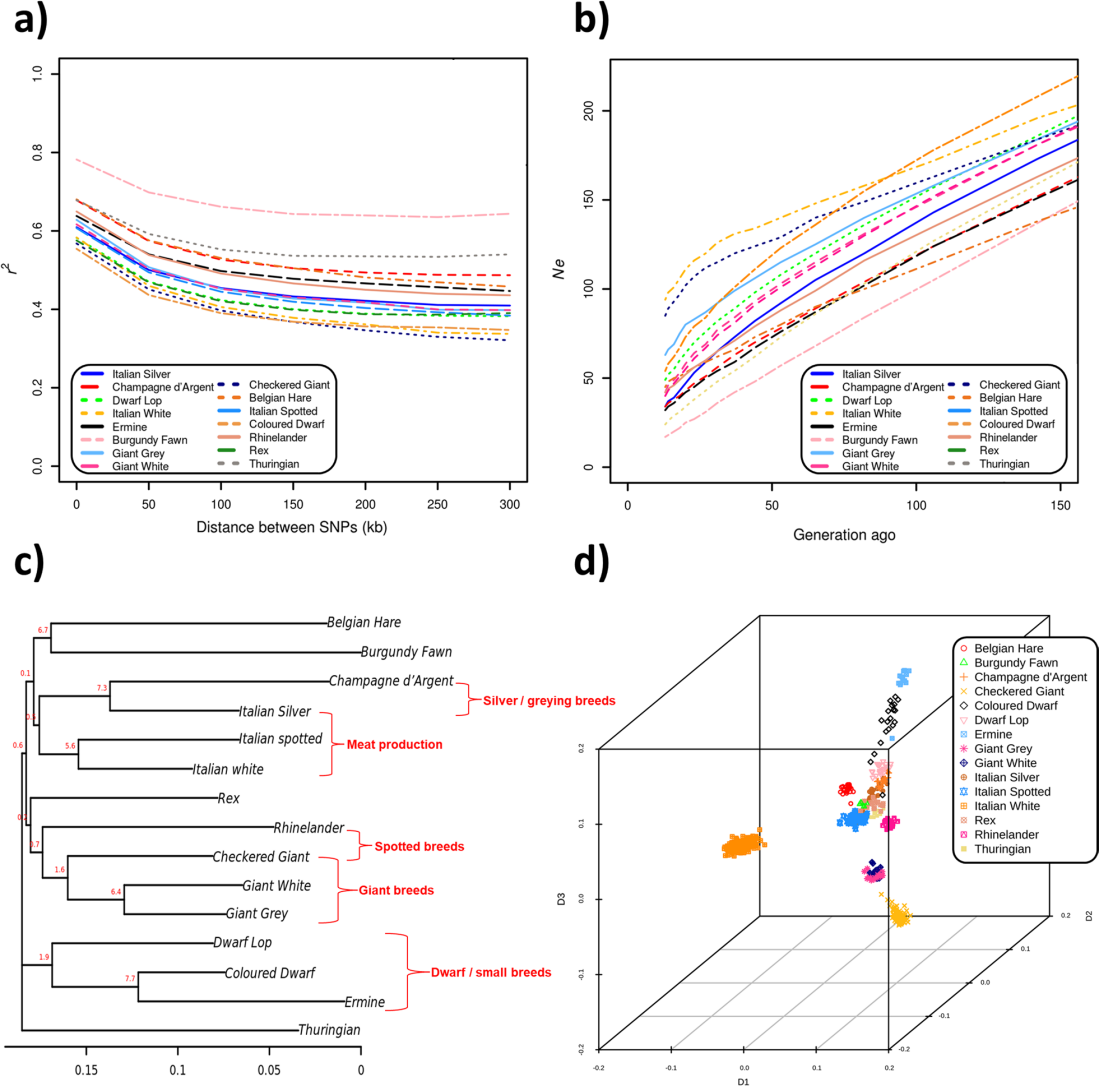
Results of population genomic analyses of 15 rabbit breeds. **a** Linkage disequilibrium (LD) decay; **b** Effective population size (N_e_); **c** Neighbor Joining (NJ) tree (next to the branches, the bootstrap test values are indicated in red, expressed as percentage over 10,000 replicates) and **d** 3D multidimensional scaling (MDS) plot

The LD decay between pairs of SNPs over a distance up to 300 kb is shown in Fig. 2a. LD dropped within the first 100 kb for all breeds and then reached approximate asymptotic but quite sizeable *r*^2^ values. The Burgundy Fawn and Thuringian breeds had the highest level of LD and the smallest effective population size (N_e_) across 50 generations [(Fig. 2b and Table 2) and (see Additional file 1: Table S3); N_e_ = 17 and N_e_ = 24, respectively]. It is worth mentioning that the results in these two breeds could be affected by their small sample sizes (less than 10). Among the other breeds, the smallest N_e_ was estimated for the Ermine, Italian Silver and Champagne d’Argent breeds (N_e_ = 32, 34 and 34, respectively) and the largest N_e_ for the Italian White (N_e_ = 134) and Checkered Giant (N_e_ = 124) breeds.

### Genetic diversity and relationships among breeds

The level of population differentiation was estimated using the fixation index F_ST,_ which was calculated as the average of pairwise breed comparisons per SNP using two methods (M1 and M2) (Table 2). The highest F_ST_ values were observed for the Burgundy Fawn and Ermine breeds for both methods (M1: 0.245 and 0.238; and M2: 0.284 and 0.282) and the lowest values for the Italian White breed (M1: 0.095; and M2: 0.216). Two groups of breeds could be identified based on the F_ST_ statistics: (i) a group of highly differentiated breeds, which included several fancy breeds, in addition to the Burgundy Fawn and Ermine breeds, i.e. the Belgian Hare, Champagne d’Argent, Rhinelander, and Thuringian breeds; and (ii) a group of less differentiated breeds that included the three commercial breeds (Italian Silver, Italian Spot and Italian White) and several fancy breeds (Checkered Giant, Coloured Dwarf, Dwarf Lop, Giant Grey, Giant White and Rex).

In the single-breed pairwise F_ST_ analyses (single-SNP-based F_ST_ matrix), the highest F_ST_ values were found for the Burgundy Fawn vs Ermine breeds (F_ST_ = 0.351) and the lowest values for the Giant White vs Giant Grey breeds (F_ST_ = 0.119) (see Additional file 1: Table S4). The NJ tree based on F_ST_ distances (Fig. 2c) clustered the analysed breeds according to their morphological features and main purpose or type. The two dwarf breeds (Coloured Dwarf and Dwarf Lop) were clustered together with the Ermine breed, which is a small-sized breed. The three giant breeds (Checkered Giant, Giant Grey and Giant White) were also grouped together. The Italian Spotted and the Italian White breeds were grouped together in a cluster that, at a lower level, also included the Italian Silver and Champagne d’Argent breeds, which contributed to the genetic pool of the Italian Spotted breed. The Burgundy Fawn and Belgian Hare breeds were located in the same cluster as the Thuringian breed, which was positioned outside all other clusters. Similar results were obtained based on the F_ST_ window-based analysis (see Additional file 1: Table S5 and Additional file 2: Fig. S1) that was also used to identify signatures of selection, as described below. The multidimensional scaling plots also clearly distinguished several breeds or groups of breeds, with a spatial disposition that resembled the NJ tree topography (Fig. 2d and see Additional file 2: Fig. S2).

### *PCAdapt* analysis: overview of markers under selection

**Fig. 3:**
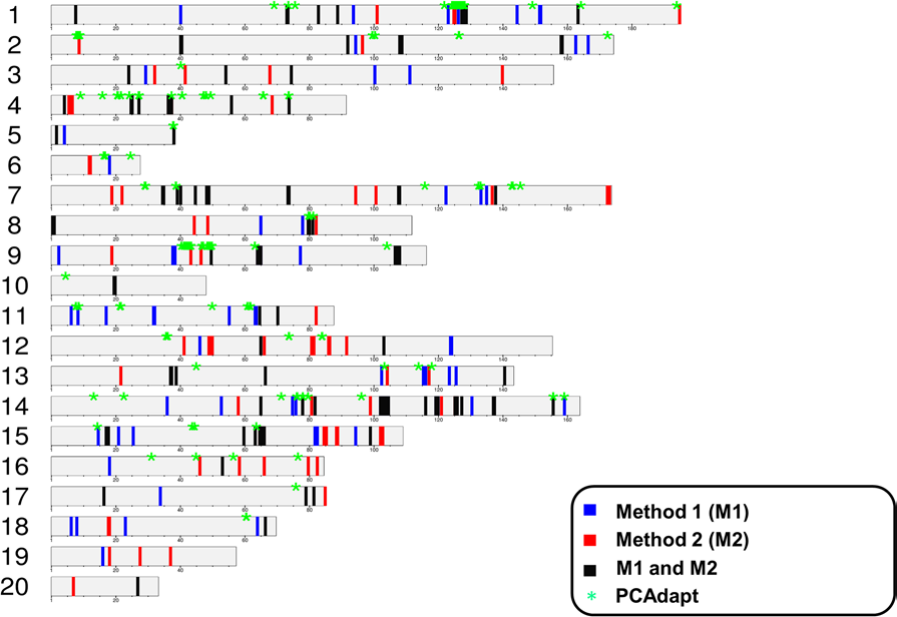
Genomic regions with signatures of selection that were identified using window-based F_ST_ analyses in the single-breed approach (Method 1 and Method 2) and *PCAdapt* analysis. Outlier windows (99.8th percentile threshold) were expanded and merged into genomic regions. Only the assembled autosomes are presented and unassembled scaffolds are not included

According to Cattell’s graphical rule, the last point before the curve flattens corresponds to the proper number of principal components that capture the population structure well [30]. Ten components (*K* = 10) were selected based on the Scree plot obtained from *PCAdapt* (see Additional file 2: Fig. S3). This explorative analysis identified 280 outlier SNPs in the full dataset with all 15 rabbit breeds. The Manhattan plot obtained from the *PCAdapt* analysis (see Additional file 2: Fig. S4) showed a few major peaks corresponding to several candidate genes that were also identified with the F_ST_ analyses (see below). These genes were located on *O. cuniculus* chromosome (OCU) 1 (*TYR*), OCU2 [*ligand dependent nuclear receptor corepressor like* (*LCORL*)/*non-SMC condensin I complex subunit G* (*NCAPG*)], OCU5 [*cadherin 13* (*CDH13*)], OCU8 [*endothelin receptor type B* (*EDNRB*)], OCU9 [*protein tyrosine phosphatase non-receptor type 2* (*PTPN2*)] and OCU14 (*LIPH*) (see Additional file 1: Table S6). The complete list of outlier markers, with overlapping or nearby annotated genes, is in Additional file 3: Table S7. Figure 3 reports the positions of these outliers markers in the rabbit genome. However, this analysis based on all breeds did not enable the potential origins of the observed signals to be directly deduced (i.e. the breeds from which they derived or how they related to the phenotypic features of the genotyped animals), although the function of some of the genes (e.g. *TYR* determines several coat colours of the *albino* series and *LIPH* affects the coat structure of the Rex breed [15, 16, 18]) provided support that the correponding peaks contained signals that would be expected based on the phenotypic characteristics of some of the investigated breeds.

### Signatures of selection detected using FST analyses of single breeds

In the analyses that compared one breed against all other breeds (Table 2) using Method 1, the window-averaged F_ST_ values across the genome ranged from 0.092 (Italian White vs all) to 0.231 (Burgundy Fawn vs all). Using Method 2, these same breeds also constituted the extremes (Italian White, F_ST_ = 0.206; Burgundy Fawn, F_ST_ = 0.287). F_ST_ values obtained from the two methods showed strong and significant correlation (*r* > 0.7, *P*-value < 2E−16) (see Additional file 1: Table S8). Statistics on the single breed window-based F_ST_ analyses (Method 1 and Method 2) are reported in Additional file 1: Table S9. The comparison of the F_ST_ values from the two methods suggested that Method 2 better highlighted genetic differences that may characterise the tested breed against all other breeds, as its F_ST_ values were always higher than those obtained with Method 1.

In these analyses considering all 15 breeds, 210 and 1050 candidate selection signature windows were identified by each method based on the 99.8th (14 windows × 15 breeds) and 99.0th (70 windows × 15 breeds) percentile thresholds, respectively. The percentages of overlapping genomic windows obtained by Method 1 and Method 2 considering all breeds were 48% (99.8th percentile) and 54% (99.0th percentile), respectively. By merging the results obtained in the analysed breeds, Method 1 identified 190 (99.8th) and 869 (99.0th) unique candidate windows and Method 2 identified 193 (99.8th) and 883 (99.0th) unique candidate windows. After merging and expanding windows detected by the two methods by ± 200 kb (Windows-M1 ∪ Windows-M2), 78 (99.8th) and 400 (99.0th) unique overlapping genomic regions were identified, comprising 47% (99.8th) and 51% (99.0th) of the overlapping genomic regions identifed by Method 1 and Method 2. Figure 3 shows the genome distribution of these regions (78 regions; 99.8th), combined for all breeds and the two methods, along with the distribution of 280 outlier SNPs obtained based on the *PCAdapt* analysis. The details for each breed are shown in Additional file 2: Fig. S5 and the Manhattan plots obtained for each breed using Method 1 and Method 2 are in Additional file 2: Figs. S6 and S7, respectively. The number of overlapping outlier regions identified using both methods ranged from two regions in the Italian Spotted and Giant Gray breeds to 11 regions in the Belgian Hare breed (see Additional file 1: Table S10). The complete lists of genome windows and regions identified from each of these analyses are in Additional file 3: Tables S11 and S12, respectively, and the list of annotated genes from the OryCun2.0 genome version is in Additional file 3: Table S13. The list of genes obtained from the single-marker-based F_ST_ analysis with the single-breed approach (Method 1 and Method 2) are in Additional file 1: Table S14 and the lists of markers obtained with Methods 1 and Method 2 are in Additional file 3: Tables S15 and S16, respectively. A summary of the most relevant results obtained by the window-based single breed approach is in Table 3 and in Additional file 1: Table S17. Several genomic regions that harbour strong candidate genes for their involvement in the determination of breed-specific features were identified with Method 1 and/or Method 2, indicating that the two methods could be considered, to some extent, as complementary in our approach based on outlier genomic windows detected with F_ST_ values against all other breeds averaged over SNPs in the window.

**Table 3:** The most important genomic regions and candidate genes derived from the window-based single-breed F_ST_ analyses

Traits	OCU:position^a^	Candidate gene	Breed (method: F_ST_ value)^b^	Previous studies^c^
Coat colour and structure	1:127,563,000–127,668,085	TYR**	Burgundy Fawn (M1: 0.79; M2*: 0.62), Italian White (M1: 0.49; M2*: 0.50), Italian Spotted (M1: 0.54)	[15, 16]
	4:5,435,027–5,439,803	ASIP**	Giant Grey (M1*: 0.49; M2: 0.53), Belgian Hare (M2*: 0.63)	[13]
	8:79,700,292–79,724,918	EDNRB**	Rhinelander (M1: 0.64; M2: 0.63)	[78]
	9:37,191,805–37,434,390	*MITF*	Giant White (M1*: 0.44; M2: 0.54)	–
	14:80,045,788–80,094,927	LIPH**	Rex (M1*: 0.45; M2: 0.56)	[18]
	15:17,113,617–17,196,501	EDNRA**	Italian White (M1: 0.54; M2: 0.60), Thuringian (M2: 0.71)	–
	17:78,036,010–78,397,189	OCA2**	Checkered Giant (M1: 0.62; M2: 0.57)	–
	Un0267:152,309–153,232	*MC1R*	Rhinelander (M2: 0.60)	[12]
Body size	2:8,357,629–8,403,513, 2:8,404,807–8,620,864	NCAPG**, LCORL**	Dwarf Lop (M2: 0.62), Ermine (M2: 0.72)	[19]
	13:36,771,716–36,779,306	MEX3A**	Coloured Dwarf (M1: 0.69; M2: 0.61), Ermine (M1*: 0.70; M2*: 0.61)	–
	13:36,843,459–36,895,416	ARHGEF2**	Coloured Dwarf (M1: 0.69; M2: 0.61), Ermine (M1*: 0.70; M2*: 0.61)	–
	13:37,616,687–37,631,494	DCST1**	Coloured Dwarf (M1: 0.69; M2:0.61), Ermine (M1*: 0.70; M2*: 0.61)	–
	13:37,644,467–37,654,971	ZBTB7B**	Coloured Dwarf (M1: 0.69; M2: 0.61), Ermine (M1*: 0.70; M2*: 0.61)	–
	13: 38,651,582–38,702,056	GATAD2B**	Coloured Dwarf (M1: 0.55; M2: 0.55)	–
	13:61,054,286–61,261,797	*COL11A1*	Coloured Dwarf (M1*: 0.44), Ermine (M1*: 0.61)	[19]
	18: 66,434,580–66,630,682	GRK5**	Dwarf Lop (M2: 0.63)	–
	Un0251:72,110–94,586	*COL2A1*	Dwarf Lop (M2: 0.57)	–

Additional information for this table is in Additional file 1: Table S17

F_ST_ values from both methods were reported (M1 and M2); in case for which the relevant identified gene was based only on one method (M1 or M2), the method is indicated

OCU: *Oryctolagus cuniculus* chromosome

^a^Position of the candidate gene, in basepairs, on the *O. cuniculus* reference genome (OryCun2.0)

^b^Method used in the single breed approach: M1 or M2. F_ST_ value of the window including the candidate gene

^c^Candidate genes previously reported in other studies in rabbit

*Top 70 windows; all other F_ST_ results are from the top 14 windows containing the reported candidate gene

**Relevant genes have been identified based on F_ST_ window-based analysis and single marker-based analysis (see Additional file 3: Tables S15 and S16)

Some of these genes have already been reported to determine coat colour in some of the investigated breeds: *TYR* (identified in the Burgundy Fawn, Italian White, and Italian Spotted breeds), a gene located on OCU1 with variants that are responsible for the allele series of the *albino* locus and characterise the breeds for which this region was highlighted [15, 16]; *ASIP*, which is located on OCU4, was identified in Giant Grey rabbits, which carry a wild type allele at the *agouti* locus, and in Belgian Hare rabbits, which carry a missense mutation in this gene [13]; *MC1R*, a gene localized in the unplaced scaffold Un0267 [also known as GL018965 (ENSEMBL) or NW_003159591 (NCBI)], which was identified in the Rhinelander breed in which it determines the *e*^*J*^ allele of the *extension* locus that causes the classical tricolour phenotype of this breed [12].

Other coat colour genes that had not yet been associated with pigmentation phenotypes in rabbit, were found in several outlier genomic windows: the *EDNRB* gene, which was also identified with the *PCAdapt* analysis, is a gene involved in Hirschsprung disease type 2 in humans; it was located in an OCU8 outlier genomic window in the Rhinelander breed; the *melanocyte inducing transcription factor* (*MITF*) gene, which is involved in regulation of melanocyte development and in transcription of melanogenesis enzyme genes, was located in an OCU9 F_ST_ extreme window in the Giant White breed; the *endothelin receptor type A* (*EDNRA*) gene, which is suggested to be involved in loss of pigmentation in goats [42], was located in an extreme F_ST_ region of OCU15 in the Italian White and Thuringian breeds; and the *OCA2 melanosomal transmembrane protein* (*OCA2*) gene, which is the homolog of the mouse *p* (pink-eyed dilution) gene that is involved in type 2 oculocutaneous albinism, was located in a OCU17 outlier region in the Checkered Giant breed.

Several additional signatures of selection were identified in genomic regions that contained genes already known to affect other external traits in rabbits. For example, in Rex rabbits, a signature of selection was identified in a region of OCU14 that harbours the *LIPH* gene, which is responsible for the effect of the *Rex*^*1*^ locus on coat structure [18]. A genomic window on OCU2 that harbours the *LCORL* and *NCAPG* genes, which are known to affect body size in several mammals (e.g. [43–48]) and was previously identified in a signature of selection in the rabbit genome [19], was detected in the Dwarf Lop and Ermine breeds. The genomic regions with the *LIPH* and *LCORL/NCAPG* genes were also identified with the *PCAdapt* analysis. Another region on OCU13 that was previously detected by Carneiro et al. [19] and harbours the *collagen type XI alpha 1 chain* (*COL11A1*) gene was identified in the Coloured Dwarf and Ermine breeds. This gene is essential in skeletal morphogenesis and variants have been associated with body height in humans [49, 50].

Other candidate genes that potentially affect body size were located in several other extreme genomic windows for some of the breeds investigated. Among these novel candidate genes (reported for the first time in this study), *collagen type II alpha 1 chain* (*COL2A1*), located in an extreme genomic region of an unassembled scaffold (Un0251) in the Dwarf Lop breed, has been reported to cause a wide spectrum of skeletal disorders in mammals, including achondrogenesis type II in humans [51–53] and the bulldog-type dwarfism in cattle [54–58]. Another region on OCU18 that carries a candidate gene involved in body size (*G protein-coupled receptor kinase 5*; *GRK5*) was also identified in the Dwarf Lop breed. Variability in the *GRK5* gene is strongly associated with body height in humans [50].

The complexity of the genetic factors that affect dwarfism and small body size was also evidenced by some differences among the three dwarf/small body size breeds. For example, a genomic window on OCU13 (position 38.50–38.85 Mb) that contains a body size-related gene (*GATA zinc finger domain containing 2B*, or *GATAD2B*; [59]) was identified in the Coloured dwarf breed. Close to this window, another window (position 37.10–37.45 Mb) was identified in this same breed (99.8th percentile) and in the Ermine breed (99.0th percentile). This window includes several genes associated with body size and height in humans: *Rho/Rac guanine nucleotide exchange factor 2* (*ARHGEF2*) associated with body size in children; *mex-3 RNA binding family member A* (*MEX3A*) associated with adult body size; *zinc finger and BTB domain containing 7B* (*ZBTB7B)*, associated with adult body size and birth weight; and *DC-STAMP domain containing 1* (*DCST1*) associated with height [50, 60, 61].

### FST analyses between groups of breeds

**Fig. 4:**
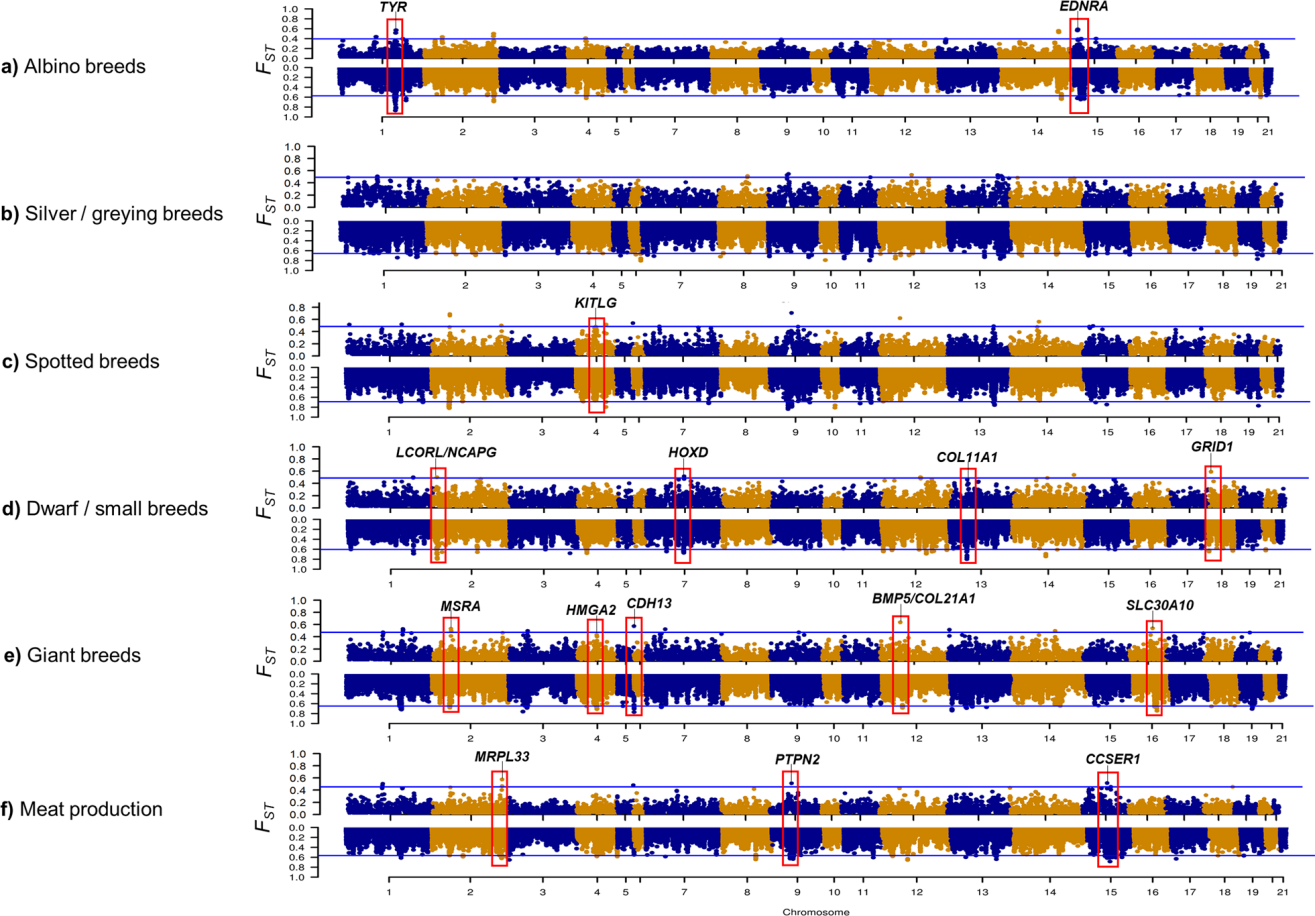
Miami plots of the genome-wide window-based (top) and single-marker-based (bottom) F_ST_ analyses of groups of breeds. **a** Albino breeds; **b** Silver/greying breeds (genes are not reported as they map to scaffolds); **c** Spotted breeds; **d** Dwarf/small breeds; **e** Giant breeds; **f** Meat rabbit lines. For single marker-based analysis: each dot represents a SNP. The blue line identifies the threshold value (99.95th percentile of the distribution). For window-based analyses: each dot represents a 350-kb genome window. The blue line identifies the threshold value (99.8th percentile of the distribution). Unassembled scaffolds are not included

To identify additional signatures of selection and to confirm regions identified in the single-breed analyses, some breeds were grouped together according to common features (shared coat colours, colour patterns, body size and use/specialization). Detailed statistics on the window-based F_ST_ analyses obtained for different groups of breeds are in Additional file 1: Table S18, and the complete list of the genomic windows and regions identified with the six pairwise group comparisons is in Additional file 3: Table S19. The most relevant genes identified in these analyses are in Table 4. The results obtained from the F_ST_ single-marker-based analysis for the groups of breeds are in Additional file 1: Table S20 and Additional file 3: Table S21. The annotated Miami plots (Fig. 4) include the most relevant results obtained from the window-based and single-marker-based F_ST_ analyses.

**Table 4:** The most important genomic regions and candidate genes derived from the window-based F_ST_ analyses of groups of breeds

Traits	Comparison	OCU:position^a^	Candidate gene	F_ST_	Previous studies^b^
Albino breeds	(Italian white + Giant White) vs all other breeds	1:127563000–127668085	TYR**	0.52	[15, 16]
		15:17113617–17196501	EDNRA**	0.58	–
Silver/graying of coat	(Italian Silver + Champagne d’Argent) vs all other breeds	Un513:25550–60684	ADNP2**	0.60	–
		Un0329:192475–292759	*NFATC1*	0.54	–
Checkered/spotted phenotype	(Checkered Giant + Rhinelander) vs all other breeds	4:67503403–67541344	*KITLG*	0.51	–
Dwarf/small body size	(Coloured Dwarf + Dwarf Lop + Ermine) vs all other breeds	2:8357629–8403513, 2:8404807–8620864	LCORL**, NCAPG**	0.50	[19]
		18:7460895–8191494	GRID1**	0.59	–
		Un0030:1681671–2068484	NTRK2**	0.54	–
		Un0030:348764–635797	FRMD3**	0.57	–
		7:115660028–115662696	*HOXD*	0.40*	[19]
		13:61054286–61261797	*COL11A1*	0.42*	[19]
Large body size	(Checkered Giant + Giant Grey + Giant White) vs all other breeds	12:45671510–45793930; 12:45977386–46101862	BMP5**, COL21A1**	0.64	–
		16:82626965–83800640, 16:53149315–53161403	CDH13**, *SLC30A10*	0.57	–
		2:39527266–39938880	MSRA**	0.53	–
		4:44715759–44848250	HMGA2**	0.40*	[19]
Meat production	Meat rabbit lines (Italian Silver + Italian Spotted + Italian White) vs all other breeds	2:158326199–158338803	MRPL33**	0.57	–
		15:58389801–59653895	CCSER1**	0.52	–
		9:49249318–49360593	PTPN2**	0.51	–
		Un0030:1681671–2068484	*NTRK2*	0.49	–

^a^Position of the candidate gene, in bp, on the *O. cuniculus* reference genome (OryCun2.0). OCU: *Oryctolagus cuniculus* chromosome

^b^Candidate genes previously reported in other studies in rabbit

*Top 70 windows; all other F_ST_ results are from the top 14 windows containing the reported candidate gene

**Relevant genes have been identified based on F_ST_ window-based analysis and single marker-based analysis (see Additional file 3: Table S21)

By grouping the two albino breeds (Giant White and Italian White), we identified a peak on OCU1 that includes the *TYR* gene (among the top 99.8th outlier genomic windows), as expected according to previous studies [15, 16]. In this comparison, an outlier genomic windows on OCU15 harbours the *EDNRA* gene [42], confirming our novel result reported in the single-breed F_ST_ analysis. The analysis that was based on the two silver/greying breeds (Champagne d’Argent and Italian Silver), which was mainly aimed at identifying genome regions that might be involved in their peculiar progressive graying of the hairs, identified outlier windows on OCU9 (two windows, 43.05–43.40 Mb and 46.20–46.55 Mb), on OCU8 (two windows from 64.4 to 65.10 Mb), on OCU13 (two windows from 115.15 to 115.85 Mb and two from 123.20 to 125.65 Mb), on OCU1 (a window from 78.05 to 78.40 Mb), on OCU12 (a window from 74.90 to 75.25 Mb), and on unassembled scaffolds (Un0088, Un0265, Un0329 and Un0513). These outlier genomic regions contain several genes that could be involved in defining this coat colour related trait (Table 4) since they play roles in the regulation of cell death (*ADNP2*; scaffold Un513) and in the control of aging in hair follicle stem cells (*NFATC1*; scaffold Un0329) [62–64]. Analysis of the two checkered spotted breeds (Checkered Giant and Rhinelander) as a group identified a signature of selection on OCU4 among the top outlier regions, which was located close to the *KIT ligand* (*KITLG*) gene. KITLG is the ligand of the tyrosine-kinase receptor encoded by the *KIT* gene that is involved in cell migration processes. As the classical spotted phenotype in these breeds has been associated with heterozygotes for a variant in the *KIT* gene [17], *KITLG* could contribute to the specific positioning of the pigmented skin areas of the checkered pattern. Notably, no signature of selection was evident in the *KIT* gene region in all comparisons, likely because the *English spotted* phenotype derives from heterozygosity at this locus [17], which might have reduced F_ST_ values for this region. The genotype information and the observed heterozygosity for variants in the *KIT* gene region are in Additional file 3: Tables S22 and S23, respectively. The mean observed heterozygosity for the two spotted breeds was (0.567 for Rhinelander and 0.626 for Checkered Giant) was close to that expected according to our previous study on this gene [17].

When the three dwarf/small breeds were considered together, a signature of selection in the OCU2 region that harbors the *LCORL* and *NCAPG* genes was again observed, in addition to regions on several other chromosomes (OCU1, 12, 14 and 18) and unassigned scaffolds (Un0030, Un0044 and Un0076), most of which include genes that were previously associated with body size and height in humans or other species. For example, the region on OCU18 includes the *glutamate ionotropic receptor delta type subunit 1* (*GRID1*) gene and the four outlier continuous windows on the unassigned scaffold Un0030 include the *neurotrophic receptor tyrosine kinase 2* (*NTRK2*) and the *FERM domain containing 3* (*FRMD3*) genes, which have been associated with body weight in humans [50]. Other regions were identified by Carneiro et al. [19] to be associated with small body size and dwarfism in rabbits. Among these regions, in our analysis, in addition to the *LCORL*/*NCAPG* region, we also confirmed the region on OCU7 containing the *HOXD* gene cluster and the region on OCU13 containing the *COL11A1* gene but only at the less stringent threshold of 99.0th percentile.

The top 99.8th genome windows that were identified when grouping the three giant breeds (Checkered Giant, Giant Grey and Giant White) were located on nine chromosomes (OCU1, 2, 3, 5, 7, 12, 13, 14 and 16) and on several unassigned scaffolds (Un0030 and Un0366). These windows include several genes related to growth traits and regulators of developmental processes. For example, the most extreme window, on OCU12, harbours the *bone morphogenetic protein 5* (*BMP5*) gene, which plays a key role in skeletal morphogenesis [65], and the *collagen type XXI alpha 1 chain* (*COL21A1*) gene, which has been associated with body size in humans [61]. The next top three windows, on OCU5, 16 and 2, harbour other genes that were previously associated with body size and height [61] [*cadherin 13* (*CDH13*), *solute carrier family 30 member 10* (*SLC30A10*) and *methionine sulfoxide reductase A* (*MSRA*), respectively]. The signal on OCU5, harbouring the *CDH13* gene, was also detected in the *PCAdapt* analysis (see Additional file 2: Fig. S4). It is also interesting to note that another genome window on OCU4 that was identified at the 99.0th percentile threshold, contained the *HMGA2* gene, which has been consistently reported to be associated with stature and body size in humans and other species [47, 66–68]. A large deletion in this gene is also responsible for a form of dwarfism in rabbit [19]. The genotype information and the observed heterozygosity values for variants in the *HMGA2* gene region are in Additional file 3: Tables S24 and S25, respectively. The mean observed heterozygosity was higher in the dwarf breeds (0.380 in Dwarf Lop, 0.731 in Coloured Dwarf and 0.593 in Ermine) than in the Giant breeds (0.127 in Giant White, 0.123 in Giant Grey and 0.124 in Checkered Giant).

The signatures of selection that were identified by combining the three meat rabbit breeds overlapped partially with those obtained in the comparisons based on groups of rabbits of similar body size. Outlier regions were identified on nine chromosomes and on two unplaced scaffolds (see Additional file 3: Table S19), which included a number of novel candidate genes. The top 99.8th genome window on OCU2 includes the *mitochondrial ribosomal protein L33* (*MRPL33*) gene, which has been reported to be a candidate gene for muscle development in ducks [69]. The second top genomic window on OCU15 contains the *coiled-coil serine rich protein 1* (*CCSER1*), which has been suggested to be involved in fat deposition in pigs [70]. Another important candidate gene in a window that was identified on OCU9, *protein tyrosine phosphatase non-receptor type 2* (*PTPN2*), confirms the results of the *PCAdapt* analysis (see Additional file 2: Fig. S4). This gene is a member of the PTP family which includes signaling molecules that regulate cell growth, differentiation, the mitotic cycle, and oncogenic transformation [71]. Another gene that controls cell differentiation (*neurotrophic receptor tyrosine kinase 2*; *NTRK2*) is located in an identified genome window on the unassigned scaffold Un0030. In humans, mutations in this gene are associated with severe obesity [72].

The over-representation analysis based on genes that were located in the extreme outlier windows (99.8th percentile) and were detected for each of the five tested groups of breeds identified three over-represented biological features (one biological process and two human phenotypes) for comparisons involving the two silver/greying breeds and the two checkered spotted breeds (see Additional file 1: Table S26). The enriched terms did not point to any relevant characteristics related to the targeted phenotypes.

## Discussion

Domestication and the subsequent directional artificial and natural selection, along with several other genetic events, have shaped the genome of all domestic animals and differentiated many breeds and populations within species. These genetic resources, which were generally constituted relatively recently, have been used to dissect the genetic mechanisms that determine extreme morphological and physiological features in several species (e.g. [36, 73–76]). The large number of rabbit breeds represents a unique and mostly unexplored resource, which can help to dissect the genetic architecture of external traits in this lagomorph species and, as a mirror, also in other mammals [1]. The rabbit is used as an animal model for applied and basic biological studies, mainly where the rodents have demonstrated several limits [77].

In this study, we investigated different fancy rabbit breeds that have characteristic phenotypes, i.e. coat colours, colour patterns, and body size and morphology. In addition, three pure breeds used to produce a three-way crossbred commercial meat line, were analysed.

As a proof of concept to demonstrate that the applied methodologies can capture recent signatures of selection, several of the results that we obtained highlight previously identified genes that affect coat colour (*ASIP*, *MC1R* and *TYR*; [11–16]), coat structure (*LIPH*; [18]), and body size (*LCORL/NCAPG*, *COL11A1* and *HOXD*; [19]) in rabbit. Other identified regions of signatures of selection also contain many novel candidate genes for phenotypic variation among the analysed breeds. Thus, complementing the candidate gene approach of previous studies in rabbit that were successful mainly for monogenic traits, the hitchhiking comparative mapping approach across breeds that we applied here, can provide a more complete view of the genetic elements that determine the main phenotypic differences in this species. In particular, many signatures of selection pointed to novel candidate genes for body size and could contribute to understand the genetic mechanisms that affect similar traits and phenotypes in other mammals. In addition, several other genes that affect coat colour and were not previously known to be involved in this phenotype in rabbits were identified in regions containing signatures of selection. Overall, 309 unique genomic regions, obtained by combining the results from single-breed analyses and analyses of groups of breeds, were identified as implicated in the most relevant genetic differences between the breeds investigated.

In spite of the insights obtained from our results, it is important to note a few limitations and how to overcome them, at least in part. Based on how the rabbit breeds have been constituted, with bottleneck effects, genetic drift, and introgression potentially playing important roles, a genome region that possesses a pattern of differentiation between breeds does not necessarily prove that the region has been under selection and has functional roles. The function of the annotated genes in the identified regions, which usually derives from what is known from other species, can be useful in some cases to interpret the obtained results. However, not all genes are functionally well described in the literature, which leaves many uncertainties, especially for complex traits or when multiple pathways are involved in the breeds’ differential phenotypes. Therefore, to extract meaningful biological features, for group-based results, we applied enrichment approaches that attempted to improve the insights into the complexity of body size and selection for productive performances. However, this analysis was not very informative, probably due to the heterogeneity of the biological mechanisms, with involvement of a large number of genes that are present in the identified genomic regions. The inclusion of additional animals per breed, additional breeds, and other breed populations, e.g. sampled in more countries (rabbit breeds are, in many cases, named according to common features but the genetic history of each national stock can differ [8, 9]) may be able to improve the comparative resolution that would be needed to reduce the noise generated by stochastic effects and demographic and specific population-derived perturbations. Other statistical methodologies that have been proposed to identify signatures of selection, along with additional genomic information (i.e. whole-genome sequencing data), could be useful to complement the approaches and data used in this study and to capture other signals of selection [6, 19]. In addition, several signals of selection were detected in unassigned scaffolds. Thus, improvement of the assembly of the rabbit reference genome is needed to better detect, at the chromosome level, signatures of selection that could have been fragmented or missed in the current study.

Our study further improves the complex puzzle of coat colour genetics in the rabbit, which was already drafted in its main framework by previous studies on candidate genes, as mentioned [1]. Signatures of selection were detected in genomic windows containing *EDNRB*, *EDNRA*, *MITF,* and *OCA2* that, to date, were not reported to affect coat colour in rabbits, although the role of these genes on coat colour is well known in several other species. We previously excluded a polymorphism in the *EDNRB* gene to be associated with the *English spotting* locus [78] but, according to our results from this study, this gene may have a role in modifying or regulating the position/extension of the bicolor (red and black) spotted patterns over the unpigmented background of the Rhinelander breed. Similar secondary roles in defining the main coat colours in the Giant White, Italian White, Thuringian, and Checkered Giant breeds, or the result of other hitchhiking effects may explain the identification of highly differentiated regions in these breeds that include *MITF*, *EDNRA,* and *OCA2*. However, the role of some of these genes in the coat colour of these breeds should be further analysed. The silvering or hereditary greying of hair, which is expected to be caused by a recessive *si* allele at the *Silver* locus (reviewed in [1]), was investigated by comparing the Champagne d’Argent and Italian Silver (a breed derived from Champagne d’Argent) breeds. No obvious candidates were reported in the outlier genomic windows that were identified in this analysis, although the function of some of the genes in these regions was directly or indirectly consistent with this coat colour phenotype. More detailed analyses, including whole-genome sequencing data, could be useful to fine map the candidate region that harbors the causative mutation(s) for this coat colour locus.

The complexity and heterogeneity of the signatures of selection that were detected in the comparisons involving the dwarf/small body size breeds, the giant breeds, and the three meat breeds indicate that a large number of genes with small effects are involved in determining body size and production performances in the rabbit, similarly to what is reported in other mammals (e.g. [42, 44–47, 49]. A few major genes (*LCORL*/*NCAPG*, the *HOXD* gene family, and *COL11A1*), which have alleles that might contribute to reducing body size in rabbit, were identified in this study, confirming the results already reported in a previous study based on Netherland Dwarf rabbits [19]. No signal in the *HMGA2* gene region was identified in the dwarf/small body size breeds that we analysed in our study, although a large deletion that overlaps the promoter region and the first three exons of this gene has been reported to be the causative mutation of a form of dwarfism [19]. This could be due to viable dwarf rabbits being heterozygous for the mutated allele, since the homozygous state is usually lethal [19]. Such a heterozygous condition that does not create extreme allele differences, may not have been captured by the F_ST_ analysis used in our study. However, this interpretation is supported by the results obtained from the observed heterozygosity analysis for variants within the *HMGA2* gene region, since the average level of heterozygosity was close to 0.5. It is also possible that, in our dwarf breeds, other loci cause this extreme reduction in size. For example, in the Dwarf Lop breed, we identified a strong signature of selection in the region of the *COL2A1* gene, which is known to be involved in a broad spectrum of skeletal defects, including dwarfism [51–54]. Identification of a signature of selection in the *HMGA2* gene region in the giant breeds was interesting and was probably due to the presence of alternative alleles for this gene that increase body size. In general, our results based on the comparisons between breeds of extreme body size demonstrate the complexity of the genetic factors that affect this phenotype, which is usually referred as a breed-specific trait in rabbit. Body size, body structure, and stature are quantitative traits for which some major genes have been identified, as already demonstrated in several other mammals [47, 48, 79–81].

Several other interesting candidate genes were detected by comparing the genomes of meat breeds with fancy breeds, which are not selected for muscle growth and performance traits. The most interesting region, which was also found with the *PCAdapt* analysis, contains the *PTPN2* gene, which is involved in several regulatory and cell differentiation mechanisms [71]. Other studies are needed to characterise the function of this gene and its role in growth and performance traits in rabbit and other livestock species.

## Conclusions

This study is the first genome-wide analysis of signatures of selection using high-density SNP genotype data in rabbits. Fifteen rabbit breeds with divergent coat colours and colour patterns, body sizes and uses/specializations were analysed. We detected several regions with significant signatures of selection, which open new avenues for further investigations to characterize the identified candidate genes and confirm their causative role. In particular, this study contributes to enlarge the number of candidate genes for body size and coat colour in this multi-purpose species. The results show that body size is also a complex trait in rabbit, and may be determined by the effects of many genes, among which some could have a potential major effect based on the analysis of signatures of selection. Other investigations with additional breeds and populations and by using different statistical approaches are needed to expand this analysis of signatures of selection in the rabbit genome. Our results will be useful to better understand rabbit domestication, which appears to be nested with the constitution of the main breeds, at least for the most recent developments. These breeds represent important genetic resources for which complete characterization at the genome level has only just started.

## Supplementary Information

**Additional file 1: Table S1.** Statistics for the window selection analysis. **Table S2.** Groups of breeds that were compared in this study. **Table S3.** Estimates of effective population size (N_e_) over time (from 13 to 142 generations ago). **Table S4.** Single-SNP-based F_ST_ distances between pairs of rabbit populations. **Table S5.** Window-based F_ST_ distances between pairs of rabbit populations. **Table S6.** The most relevant results obtained from the *PCAdapt* analysis that overlap with those of the F_ST_ analyses. **Table S8.** Pearson’s correlations for genome windows F_ST_ values obtained from Method 1 and Method 2 (*P*-value < 2E−16). The reported values are the means of the correlations of the SNPs across all the chromosomes and all the genome windows. **Table S9.** Statistics on the single-breed window-based F_ST_ analyses (Method 1 and Method 2). **Table S10.** Statistics on the identified genome regions from the single-breed window-based F_ST_ analyses. **Table S14.** List of relevant genes identified with the F_ST_ single-marker-based analysis in the single-breed approach based on the two methods also applied in the window-based analyses (Method 1 and Method 2). F_ST_ values of the markers at the extreme lower end of the distributions (99.95th percentile) and mapped to genes of interest are presented. **Table S17.** Genome regions including candidate genes and the total number of genes included in the genome windows identified with the window-based single-breed F_ST_ analysis. This table is complementary to Table 3. **Table S18.** Statistics for the window-based F_ST_ analyses in the approach based on groups of breeds. **Table S20.** List of relevant genes identified with the F_ST_ single-marker-based analysis in the approach based on groups of breeds. F_ST_ values of the markers at the extreme lower end of the distributions (99.95th percentile) and mapped to genes of interest are presented. **Table S26.** Gene enrichment analysis. Gene sets (99.0th percentile) related to the group-based F_ST_ analyses were tested for over-represented biological features.

**Additional file 2: Figure S1.** Window-based Neighbor Joining tree. **Figure S2.** Multidimensional scaling plot. The first three components are provided. **Figure S3.** Scree plot used to identify the number of principal components that describe well the population structure of the investigated rabbit breeds. The plot displays in decreasing order the percentage of variance explained by each principal component. **Figure S4.** Manhattan plots of the *PCAdapt* analysis. Each dot represents a 350-kb genome window. The red line identifies the threshold value (0.1 Bonferroni corrected *P*-value). Unassembled scaffolds are not reported. **Figure S5.** Genome regions carrying signatures of selection (99.8th percentile; expanded windows) identified in the studied breeds. Only the assembled autosomes are presented and unassembled scaffolds are not reported. **Figure S6.** Manhattan plots of the genome-wide F_ST_ analyses based on Method 1 (M1). Each dot represents a 350-kb genome window. The blue line identifies the threshold value (99.8th percentile of the distribution). Unassembled scaffolds are not reported. **Figure S7.** Manhattan plots of the genome-wide F_ST_ analyses based on Method 2 (M2). Each dot represents a 350-kb genome window. The blue line identifies the threshold value (99.8th percentile of the distribution). Unassembled scaffolds are not reported.

**Additional file 3: Table S7.** List of outlier markers identified using *PCAdapt*. **Table S11.** Comparative single-breed F_ST_ analysis (M1 = Method 1). The genome windows at the extreme lower end of the distributions (99.0th percentile) are presented. **Table S12.** Comparative single-breed F_ST_ analysis (M2 = Method 2). The genome windows at the extreme lower end of the distributions (99.0th percentile) are presented. **Table S13.** Genome regions identified in the single-breed F_ST_ analyses (99.8th percentile). **Table S15.** List of the markers at the extreme lower end of the distributions (99.95th percentile) identified with the F_ST_ single-marker-based analysis for the single-breed approach using Method 1 (M1). SNPs are reported in decreasing order of F_ST_ value. **Table S16.** List of the markers at the extreme lower end of the distributions (99.95th percentile) identified with the F_ST_ single-marker-based analysis for the single-breed approach using Method 2 (M2). SNPs are reported in decreasing order of F_ST_ value. **Table S19.** Comparative F_ST_ analysis for groups of breeds as defined in Additional file 1: Table S2. The genome windows at the extreme lower end of the distributions (99.0th percentile) are presented. **Table S21.** List of the markers at the extreme lower end of the distributions (99.95th percentile) identified with the F_ST_ single-marker-based analysis using the approach that includes groups of breeds. SNPs are reported in decreasing order of F_ST_ value. SNPs are reported in decreasing order of F_ST_ value. **Table S22.** Genotype information for SNPs within the *KIT* gene region. **Table S23.** Observed heterozygosity of SNPs within the *KIT* gene region. **Table S24.** Genotype information for SNPs within the *HMGA2* gene region. **Table S25.** Observed heterozygosity of SNPs within the *HMGA2* gene region.

## Data Availability

The datasets used and analysed during the current study are available from the corresponding author upon reasonable request and can be shared after an agreement on their use with University of Bologna and ANCI.
