# Supplementary material for: Genomic diversity and signatures of selection in meat and fancy rabbit breeds based on high-density marker data
Source: Genet Sel Evol. 2022 Jan 21;54:3. doi: 10.1186/s12711-022-00696-9 (PMC8780294; doi:10.1186/s12711-022-00696-9)
Supplement: Supplementary file 1 — Additional file 1: Table S1. Statistics for the window selection analysis. Table S2. Groups of breeds that were compared in this study. Table S3. Estimates of effective population size (Ne) over time (from 13 to 142 generations ago). Table S4. Single-SNP-based FST distances between pairs of rabbit populations. Table S5. Window-based FST distances between pairs of rabbit populations. Table S6. The most relevant results obtained from the PCAdapt analysis that overlap with those of the FST analyses. Table S8. Pearson’s correlations for genome windows FST values obtained from Method 1 and Method 2 (P-value < 2E−16). The reported values are the means of the correlations of the SNPs across all the chromosomes and all the genome windows. Table S9. Statistics on the single-breed window-based FST analyses (Method 1 and Method 2). Table S10. Statistics on the identified genome regions from the single-breed window-based FST analyses. Table S14. List of relevant genes identified with the FST single-marker-based analysis in the single-breed approach based on the two methods also applied in the window-based analyses (Method 1 and Method 2). FST values of the markers at the extreme lower end of the distributions (99.95th percentile) and mapped to genes of interest are presented. Table S17. Genome regions including candidate genes and the total number of genes included in the genome windows identified with the window-based single-breed FST analysis. This table is complementary to Table 3. Table S18. Statistics for the window-based FST analyses in the approach based on groups of breeds. Table S20. List of relevant genes identified with the FST single-marker-based analysis in the approach based on groups of breeds. FST values of the markers at the extreme lower end of the distributions (99.95th percentile) and mapped to genes of interest are presented. Table S26. Gene enrichment analysis. Gene sets (99.0th percentile) related to the group-based FST analyses were tested for over-repre [file 12711_2022_696_MOESM1_ESM.doc]

**Table S1 Statistics for the window selection analysis**

| **Window size (bp)** | **Number of windows** | | **Number of SNPs** | | |
| --- | --- | --- | --- | --- | --- |
|  | **Total** | **<3 SNPs** | **Mean** | **Median** | **Standard deviation** |
| 50000 | 47,901 | 20,122 | 2.9 | 3 | 1.3 |
| 100000 | 25,605 | 3044 | 5.0 | 5 | 2.4 |
| 150000 | 17,478 | 1154 | 7.9 | 8 | 3.3 |
| 200000 | 13,339 | 749 | 10.4 | 11 | 4.3 |
| 250000 | 10,839 | 589 | 12.8 | 13 | 5.3 |
| 300000 | 9158 | 509 | 15.2 | 16 | 6.0 |
| 350000 | 7951 | 479 | 17.5 | 19 | 7.4 |
| 400000 | 7060 | 448 | 19.7 | 21 | 8.5 |
| 450000 | 6364 | 440 | 21.0 | 23 | 9.7 |
| 500000 | 5801 | 438 | 24.0 | 26 | 10.9 |

**Table S2** **Groups of breeds that were compared in this study**

| **Trait/feature used to define the groups** | **First group of breeds** | **Second group of breeds** | **Comments** |
| --- | --- | --- | --- |
| Coat colours/patterns | Albino breeds (Italian White + Giant White) | All other breeds |  |
|  | Silver/greying breeds (Italian Silver + Champagne d’Argent) | All other breeds | - |
|  | Spotted breeds (Checkered Giant + Rhinelander) | All other breeds | The two spotted breeds have the checkered pattern |
| Body size | Dwarf/small breeds (Coloured Dwarf + Dwarf Lop + Ermine) | All other breeds | - |
|  | Giant breeds (Checkered Giant + Giant Grey + Giant White) | All other breeds | - |
| Use | Meat rabbit lines (Italian Silver + Italian Spotted + Italian White) | All other breeds | - |

**Table S3 Estimates of effective population size (N_e_) over time (from 13 to 142 generations ago)**

|  | **Generation ago** | | | | | | | | | | | | | | |
| --- | --- | --- | --- | --- | --- | --- | --- | --- | --- | --- | --- | --- | --- | --- | --- |
|  | **13** | **14** | **16** | **18** | **20** | **23** | **27** | **31** | **37** | **44** | **53** | **65** | **82** | **106** | **142** |
| **Meat breeds** |  |  |  |  |  |  |  |  |  |  |  |  |  |  |  |
| Italian Silver | 34 | 37 | 39 | 43 | 47 | 53 | 59 | 65 | 73 | 82 | 92 | 105 | 120 | 143 | 173 |
| Italian Spotted | 43 | 45 | 47 | 50 | 53 | 56 | 60 | 64 | 71 | 78 | 86 | 95 | 106 | 123 | 144 |
| Italian White | 94 | 98 | 101 | 106 | 111 | 116 | 120 | 126 | 131 | 134 | 140 | 148 | 158 | 172 | 196 |
| **Fancy breeds** |  |  |  |  |  |  |  |  |  |  |  |  |  |  |  |
| Belgian Hare | 45 | 48 | 50 | 51 | 53 | 56 | 59 | 62 | 66 | 73 | 80 | 90 | 100 | 115 | 138 |
| Burgundy Fawn | 17 | 18 | 20 | 22 | 25 | 27 | 31 | 36 | 42 | 48 | 57 | 68 | 84 | 105 | 137 |
| Champagne d'Argent | 34 | 36 | 38 | 40 | 43 | 47 | 51 | 56 | 63 | 70 | 78 | 89 | 104 | 124 | 152 |
| Checkered Giant | 85 | 89 | 93 | 97 | 102 | 107 | 112 | 115 | 120 | 124 | 129 | 140 | 149 | 163 | 184 |
| Coloured Dwarf | 54 | 58 | 62 | 67 | 73 | 79 | 84 | 92 | 101 | 111 | 123 | 136 | 155 | 178 | 208 |
| Dwarf Lop | 49 | 52 | 55 | 60 | 64 | 70 | 76 | 82 | 89 | 98 | 108 | 120 | 136 | 157 | 186 |
| Ermine | 32 | 34 | 36 | 39 | 42 | 45 | 50 | 54 | 59 | 67 | 76 | 87 | 102 | 124 | 151 |
| Giant Grey | 63 | 66 | 69 | 75 | 80 | 83 | 87 | 92 | 99 | 106 | 115 | 125 | 140 | 158 | 184 |
| Giant White | 42 | 46 | 49 | 53 | 58 | 64 | 69 | 77 | 84 | 93 | 103 | 115 | 131 | 151 | 181 |
| Rex | 40 | 43 | 47 | 51 | 55 | 61 | 67 | 74 | 81 | 91 | 101 | 113 | 130 | 152 | 181 |
| Rhinelander | 42 | 44 | 46 | 49 | 52 | 56 | 60 | 65 | 71 | 79 | 88 | 99 | 116 | 135 | 163 |
| Thuringian | 24 | 26 | 29 | 31 | 34 | 38 | 43 | 48 | 54 | 62 | 73 | 86 | 103 | 127 | 159 |

**Table S4** **Single SNP based F_ST_ distances between pairs of rabbit populations**

| **Breed^1^** | **BF** | **BH** | **CD** | **CdA** | **CG** | **DL** | **ER** | **GG** | **GW** | **ISI** | **ISP** | **IW** | **RE** | **RH** | **TH** |
| --- | --- | --- | --- | --- | --- | --- | --- | --- | --- | --- | --- | --- | --- | --- | --- |
| **BF** | - |  |  |  |  |  |  |  |  |  |  |  |  |  |  |
| **BH** | 0.323 | - |  |  |  |  |  |  |  |  |  |  |  |  |  |
| **CD** | 0.294 | 0.277 | - |  |  |  |  |  |  |  |  |  |  |  |  |
| **CdA** | 0.329 | 0.324 | 0.278 | - |  |  |  |  |  |  |  |  |  |  |  |
| **CG** | 0.287 | 0.272 | 0.221 | 0.270 | - |  |  |  |  |  |  |  |  |  |  |
| **DL** | 0.290 | 0.274 | 0.178 | 0.274 | 0.208 | - |  |  |  |  |  |  |  |  |  |
| **ER** | 0.351 | 0.341 | 0.160 | 0.342 | 0.292 | 0.247 | - |  |  |  |  |  |  |  |  |
| **GG** | 0.293 | 0.282 | 0.229 | 0.273 | 0.163 | 0.214 | 0.299 | - |  |  |  |  |  |  |  |
| **GW** | 0.297 | 0.286 | 0.238 | 0.280 | 0.175 | 0.224 | 0.306 | 0.119 | - |  |  |  |  |  |  |
| **ISI** | 0.294 | 0.282 | 0.236 | 0.194 | 0.225 | 0.230 | 0.299 | 0.231 | 0.236 | - |  |  |  |  |  |
| **ISP** | 0.302 | 0.262 | 0.232 | 0.282 | 0.218 | 0.226 | 0.297 | 0.220 | 0.228 | 0.188 | - |  |  |  |  |
| **IW** | 0.276 | 0.235 | 0.202 | 0.258 | 0.185 | 0.195 | 0.276 | 0.186 | 0.197 | 0.193 | 0.146 | - |  |  |  |
| **RE** | 0.288 | 0.266 | 0.216 | 0.265 | 0.205 | 0.211 | 0.280 | 0.210 | 0.218 | 0.225 | 0.223 | 0.193 | - |  |  |
| **RH** | 0.317 | 0.300 | 0.247 | 0.301 | 0.214 | 0.235 | 0.310 | 0.236 | 0.241 | 0.255 | 0.251 | 0.227 | 0.233 | - |  |
| **TH** | 0.327 | 0.316 | 0.262 | 0.315 | 0.250 | 0.257 | 0.322 | 0.260 | 0.263 | 0.275 | 0.267 | 0.252 | 0.260 | 0.290 | - |

**^1^**Full breed names are given in Table 1 of main text.

**Table S5** **Window-based F_ST_ distances between pairs of rabbit populations**

| **Breed^1^** | **BF** | **BH** | **CD** | **CdA** | **CG** | **DL** | **ER** | **GG** | **GW** | **ISI** | **ISP** | **IW** | **RE** | **RH** | **TH** |
| --- | --- | --- | --- | --- | --- | --- | --- | --- | --- | --- | --- | --- | --- | --- | --- |
| **BF** | - |  |  |  |  |  |  |  |  |  |  |  |  |  |  |
| **BH** | 0.310 | - |  |  |  |  |  |  |  |  |  |  |  |  |  |
| **CD** | 0.281 | 0.268 | - |  |  |  |  |  |  |  |  |  |  |  |  |
| **CdA** | 0.317 | 0.316 | 0.266 | - |  |  |  |  |  |  |  |  |  |  |  |
| **CG** | 0.272 | 0.263 | 0.211 | 0.259 | - |  |  |  |  |  |  |  |  |  |  |
| **DL** | 0.275 | 0.264 | 0.168 | 0.260 | 0.198 | - |  |  |  |  |  |  |  |  |  |
| **ER** | 0.340 | 0.332 | 0.149 | 0.331 | 0.282 | 0.237 | - |  |  |  |  |  |  |  |  |
| **GG** | 0.279 | 0.271 | 0.218 | 0.262 | 0.154 | 0.203 | 0.288 | - |  |  |  |  |  |  |  |
| **GW** | 0.282 | 0.274 | 0.225 | 0.267 | 0.164 | 0.212 | 0.292 | 0.109 | - |  |  |  |  |  |  |
| **ISI** | 0.279 | 0.271 | 0.225 | 0.182 | 0.215 | 0.219 | 0.288 | 0.220 | 0.223 | - |  |  |  |  |  |
| **ISP** | 0.288 | 0.253 | 0.224 | 0.272 | 0.210 | 0.218 | 0.290 | 0.211 | 0.218 | 0.181 | - |  |  |  |  |
| **IW** | 0.262 | 0.227 | 0.194 | 0.248 | 0.179 | 0.187 | 0.267 | 0.178 | 0.187 | 0.184 | 0.141 | - |  |  |  |
| **RE** | 0.272 | 0.256 | 0.205 | 0.251 | 0.193 | 0.199 | 0.269 | 0.199 | 0.205 | 0.215 | 0.213 | 0.183 | - |  |  |
| **RH** | 0.303 | 0.289 | 0.236 | 0.289 | 0.205 | 0.225 | 0.300 | 0.227 | 0.229 | 0.246 | 0.242 | 0.219 | 0.221 | - |  |
| **TH** | 0.312 | 0.303 | 0.248 | 0.301 | 0.236 | 0.243 | 0.309 | 0.245 | 0.248 | 0.261 | 0.256 | 0.239 | 0.245 | 0.277 | - |

**^1^**Full breed names are given in Table 1 of the main text.

**Table S6 The most relevant results obtained from the *PCAdapt* analysis that overlap with those of the F_ST_ analyses**

| **Chr** | **SNP** | **POS** | **adj-pvalue^1^** | ***P*-value^2^** | **Closet gene** |
| --- | --- | --- | --- | --- | --- |
| 1 | AX-147089636 | 125,419,461 | 2.52E-08 | 1.84E-13 | *TYR* |
| 9 | AX-146989806 | 49,359,540 | 3.14E-08 | 7.09E-12 | *PTPN2* |
| 8 | AX-146982661 | 79,705,280 | 0.000969 | 7.07E-09 | *EDNRB* |
| 14 | AX-147100373 | 96,112,330 | 1.80E-07 | 1.27E-07 | *LIPH* |
| 2 | AX-147084041 | 99,574,468 | 1.77E-08 | 1.68E-07 | *LCORL/NCAPG* |
| 5 | AX-147025349 | 37,916,383 | 9.05E-06 | 4.03E-07 | *CDH13* |

^1^ Adjusted *P*-value based on Bonferroni correction.

^2^ *P*-value of Mahalanobis distances statistical test obtained from *PCAdapt*.

**Table S8 Pearson's correlations for genome windows F_ST_ values obtained from Method 1 and Method 2 (*P*-value < 2E-16)**

**The reported values are the means of the correlations of the SNPs across all the chromosomes and all the genome windows.**

| **Breed** | **Pearson's correlation (Chr)^1^** | **Pearson's correlation (Windows)^2^** |
| --- | --- | --- |
| **Meat breeds** |  |  |
| Italian Silver | 0.821 | 0.771 |
| Italian Spotted | 0.765 | 0.792 |
| Italian White | 0.709 | 0.830 |
| **Fancy breeds** |  |  |
| Belgian Hare | 0.871 | 0.874 |
| Burgundy Fawn | 0.853 | 0.849 |
| Champagne d’Argent | 0.879 | 0.869 |
| Checkered Giant | 0.750 | 0.760 |
| Coloured Dwarf | 0.797 | 0.806 |
| Dwarf Lop | 0.769 | 0.804 |
| Ermine | 0.883 | 0.881 |
| Giant Grey | 0.749 | 0.791 |
| Giant White | 0.784 | 0.792 |
| Rex | 0.771 | 0.796 |
| Rhinelander | 0.844 | 0.835 |
| Thuringian | 0.844 | 0.852 |

^1^ The correlation between F_ST_ values of chromosomal means.

^2^ The correlation between F_ST_ values of genome windows.

**Table S9 Statistics on the single breed window-based F_ST_ analyses (Method 1 and Method 2)**

| **Breed** | **F_ST_ - Method 1 (M1)** | | | | | | | **F_ST_ - Method 2 (M2)** | | | | | | |
| --- | --- | --- | --- | --- | --- | --- | --- | --- | --- | --- | --- | --- | --- | --- |
|  | **T_99.8_^1^** | **T_99.0_^2^** | **Mean** | **s.d.^3^** | **Median** | **Min^4^** | **Max^5^** | **T_99.8_** | **T_99.0_** | **Mean** | **s.d.** | **Median** | **Min** | **Max** |
| **Meat breeds** | | | | | | | | | | | | | | |
| Italian Silver | 0.468 | 0.392 | 0.137 | 0.084 | 0.123 | 0.001 | 0.551 | 0.504 | 0.433 | 0.228 | 0.220 | 0.036 | 0.468 | 0.565 |
| Italian Spotted | 0.470 | 0.399 | 0.124 | 0.084 | 0.106 | 0.001 | 0.550 | 0.515 | 0.450 | 0.229 | 0.218 | 0.026 | 0.470 | 0.619 |
| Italian White | 0.439 | 0.338 | 0.092 | 0.072 | 0.073 | 0.001 | 0.600 | 0.497 | 0.407 | 0.206 | 0.197 | 0.021 | 0.439 | 0.624 |
| **Fancy breeds** | | | | | | | | | | | | | | |
| Belgian Hare | 0.673 | 0.567 | 0.201 | 0.126 | 0.178 | 0.002 | 0.865 | 0.700 | 0.580 | 0.277 | 0.258 | 0.028 | 0.673 | 0.864 |
| Burgundy Fawn | 0.698 | 0.599 | 0.231 | 0.128 | 0.212 | 0.004 | 0.850 | 0.694 | 0.570 | 0.287 | 0.273 | 0.018 | 0.698 | 0.808 |
| Champagne d’Argent | 0.660 | 0.575 | 0.210 | 0.127 | 0.187 | 0.002 | 0.798 | 0.626 | 0.551 | 0.271 | 0.256 | 0.026 | 0.660 | 0.775 |
| Checkered Giant | 0.527 | 0.434 | 0.132 | 0.095 | 0.110 | <0.001 | 0.699 | 0.512 | 0.429 | 0.217 | 0.205 | 0.030 | 0.527 | 0.641 |
| Coloured Dwarf | 0.549 | 0.438 | 0.143 | 0.092 | 0.123 | 0.001 | 0.693 | 0.502 | 0.428 | 0.222 | 0.213 | 0.008 | 0.549 | 0.612 |
| Dwarf Lop | 0.550 | 0.426 | 0.137 | 0.093 | 0.116 | 0.001 | 0.698 | 0.508 | 0.433 | 0.221 | 0.211 | 0.018 | 0.550 | 0.664 |
| Ermine | 0.704 | 0.600 | 0.228 | 0.129 | 0.209 | 0.001 | 0.855 | 0.669 | 0.577 | 0.282 | 0.268 | 0.022 | 0.704 | 0.755 |
| Giant Grey | 0.486 | 0.407 | 0.130 | 0.087 | 0.111 | 0.001 | 0.598 | 0.492 | 0.422 | 0.219 | 0.209 | 0.014 | 0.486 | 0.558 |
| Giant White | 0.495 | 0.419 | 0.136 | 0.091 | 0.116 | 0.002 | 0.615 | 0.506 | 0.439 | 0.224 | 0.212 | 0.012 | 0.495 | 0.635 |
| Rex | 0.530 | 0.427 | 0.136 | 0.091 | 0.115 | 0.001 | 0.719 | 0.513 | 0.435 | 0.223 | 0.212 | 0.026 | 0.530 | 0.688 |
| Rhinelander | 0.605 | 0.492 | 0.174 | 0.109 | 0.152 | 0.002 | 0.763 | 0.591 | 0.506 | 0.255 | 0.240 | 0.051 | 0.605 | 0.708 |
| Thuringian | 0.667 | 0.538 | 0.200 | 0.117 | 0.181 | 0.001 | 0.878 | 0.662 | 0.537 | 0.264 | 0.249 | 0.040 | 0.667 | 0.750 |

^1^F_ST_ values corresponding to the 99.8^th^ percentile; ^2^F_ST_ values corresponding to the 99.0^th^ percentile; ^3^Standard deviation; ^4^Minimum; ^5^ Maximum.

**Table S10 Statistics on the identified genome regions from single breed window-based F_ST_ analyses**

| **Breed** | **No. of genome windows (99.8^th^ percentile)** | | | **No. of genome regions (99.8^th^ percentile)** | | |
| --- | --- | --- | --- | --- | --- | --- |
|  | **Method 1 (M1)** | **Method 2 (M2)** | **Overlap M1 and M2** | **Method 1 (M1)** | **Method 2 (M2)** | **Overlap M1 and M2** |
| **Meat breeds** | | | | | | |
| Italian Silver | 14 | 14 | 7 | 13 | 11 | 5 |
| Italian Spotted | 14 | 14 | 2 | 14 | 12 | 2 |
| Italian White | 14 | 14 | 10 | 9 | 8 | 6 |
| **Fancy breeds** | | | | | | |
| Belgian Hare | 14 | 14 | 11 | 13 | 13 | 11 |
| Burgundy Fawn | 14 | 14 | 10 | 12 | 13 | 6 |
| Champagne d'Argent | 14 | 14 | 5 | 7 | 9 | 3 |
| Checkered Giant | 14 | 14 | 7 | 13 | 10 | 6 |
| Coloured Dwarf | 14 | 14 | 8 | 12 | 15 | 7 |
| Dwarf Lop | 14 | 14 | 8 | 10 | 11 | 6 |
| Ermine | 14 | 14 | 9 | 14 | 14 | 8 |
| Giant Grey | 14 | 14 | 2 | 10 | 10 | 2 |
| Giant White | 14 | 14 | 5 | 12 | 13 | 4 |
| Rex | 14 | 14 | 5 | 11 | 11 | 4 |
| Rhinelander | 14 | 14 | 6 | 9 | 9 | 4 |
| Thuringian | 14 | 14 | 6 | 8 | 8 | 4 |
| **Total** | 210 | 210 | 101 | 167 | 167 | 78 |

| **Traits** | **Gene** | **OCU:position^1^** | **Breed^2^** | **Method: F_ST_ value and Ranking ^3^** |
| --- | --- | --- | --- | --- |
| Coat colour and structure | *TYR* | 1:127563000-127668085 | Italian Spotted  Italian White  Burgundy Fawn | M1= 0.892, R 1; M2 = 0.850, R1  M1 = 0.811, R 1; M2 = 0.799, R1  M1 = 0.861, R 36 |
|  | *ASIP* | 4:5435027-5439803 | Giant Grey  Belgian Hare | M1 = 0.698, R 33; M2 = 0.836, R 1  M1 = 0.943, R1; M2 = 0.955, R1 |
|  | *EDNRB* | 8:79700292-79724918 | Rhinelander | M1 = 0.947, R 1; M2 = 0.876, R 2 |
|  | *LIPH* | 14: 80045788-80094927 | Rex | M1 = 0.765, R 31; M2 = 0.857, R 11 |
|  | *EDNRA* | 15: 17113617-17196501 | Italian White  Thuringian | M1 = 0.669, R 16; M2 = 0.685, R 17  M2 = 0.793, R 70 |
|  | *OCA2* | 17:78036010-78397189 | Checkered Giant | M1 = 0.755, R 36; M2 = 0.703, R 24 |
| Body size | *NCAPG, LCORL* | 2:8357629-8403513, 2:8404807-8620864 | Dwarf Lop  Ermine | M1 = 0.913, R 1; M2 = 0.855, R 6  M1 = 0.857, R 65 |
|  | *MEX3A* | 13:36771716-36779306 | Coloured Dwarf | M1 = 0.722, R 50; M2 = 0.688, R 40 |
|  | *ARHGEF2* | 13:36843459-36895416 | Coloured Dwarf | M1 = 0.722, R 50; M2 = 0.688, R 40 |
|  | *DCST1* | 13:37616687-37631494 | Coloured Dwarf | M1 = 0.709, R 58 |
|  | *ZBTB7B* | 13:37644467-37654971 | Coloured Dwarf | M1 = 0.709, R 58 |
|  | *GRK5* | 18: 66434580-66630682 | Dwarf Lop | M1 = 0.803, R 12; M2 = 0.707, R 30 |

**Table S14 List of relevant genes identified with the F_ST_ single marker-based analysis in the single breed approach based on the two methods also applied in the window-based analyses (Method 1 and Method 2)**

F_ST_ values of the markers at the extreme lower end of the distributions (99.95^th^ percentile) and mapped to genes of interest are presented

^1^Position of the candidate gene, in basepairs, on the *O. cuniculus* reference genome (OryCun2.0). OCU = *Oryctolagus cuniculus* chromosome.

^2^Method used in the single breed analysis: M1 or M2. F_ST_ value of the top significant marker mapped to the candidate gene and ranking of this SNP in the list of the 99.95^th^ percentile top SNP. In a few cases the relevant gene was identified with one of the two methods. The complete list of the 99.95^th^ percentile SNPs is reported in Table S15 (M1) and Table S16 (M2).

| **Traits** | **Genome regions^1^** | **Gene** | **Breed (Method:F_ST_)^2^** | **Size^3^** | **Number of genes^4^** |
| --- | --- | --- | --- | --- | --- |
| Coat colour and structure | 1: 127050001-128450000 | *TYR* | Burgundy Fawn (M1:0.785; M2*: 0.623), Italian White (M1:0.491; M2*:0.495), Italian Spotted (M1:0.539) | 1.399 | 7 |
|  | 4: 4900001 -5950000 | *ASIP* | Giant Grey (M1*:0.486; M2: 0.517) | 1.0499 | 18 |
|  | 8: 79100001-80500000 | *EDNRB* | Rhinelander (M1:0.640; M2:0.627) | 1.399 | 5 |
|  | 9: 37100001-37450000 | *MITF* | Giant White (M1*: 0.435; M2:0.538) | 0.349 | 1 |
|  | 14: 80500001-80850000 | *LIPH* | Rex (M1*:0.446; M2:0.559) | 0.349 | 3 |
|  | 15: 17150001-17500000 | *EDNRA* | Italian White (M1:0.544; M2:0.596), Thuringian (M2:0.711) | 0.349 | 1 |
|  | 17: 77700001-78050000 | *OCA2* | Checkered Giant (M1:0.617; M2:0.568) | 0.349 | 5 |
|  | Un0267: 1-350000 | *MC1R* | Rhinelander (M2:0.601) | 0.349 | 14 |
| Body size | 2: 7700001-8750000 | *NCAPG, LCORL* | Dwarf Lop (M2:0.615), Ermine (M2:0.724) | 1.049 | 9 |
|  | 13: 36050001-37450000 | *MEX3A* | Coloured Dwarf (M1:0.693; M2:0.608), Ermine (M1*:0.704; M2*:0.614) | 1.399 | 60 |
|  | 13: 36050001-37450000 | *ARHGEF2* | Coloured Dwarf (M1:0.693; M2:0.608), Ermine (M1*:0.704; M2*:0.614) | 1.399 | 60 |
|  | 13: 37100001-37450000 | *DCST1* | Coloured Dwarf (M1:0.693; M2:0.608), Ermine (M1*:0.704; M2*:0.614) | 0.399 | 10 |
|  | 13: 37100001-37450000 | *ZBTB7B* | Coloured Dwarf (M1:0.693; M2:0.608), Ermine (M1*:0.704; M2*:0.614) | 0.399 | 10 |
|  | 13: 38500001-39550000 | *GATAD2B* | Coloured Dwarf (M1:0.553; M2:0.546) | 1.049 | 45 |
|  | 13: 61250001 -61950000 | *COL11A1* | Coloured Dwarf (M1*:0.442), Ermine (M1*:0.611) | 0.699 | 1 |
|  | 18: 66150001-66500000 | *GRK5* | Dwarf Lop (M1*: 0.698, M2:0.625) | 0.349 | 6 |
|  | Un0251:1-350000 | *COL2A1* | Dwarf Lop (M1*: 0.532, M2:0.573) | 0.349 | 10 |

**Table S17** **Genome regions including candidate genes and the total number of genes included in the genome windows identified with the window-based single breed F_ST_ analysis (t**his table is complementary to Table 3)

^1^ Position of the genome window, in basepairs, The overlapped windows with the windows identified for each breed is presented.

^2^ Method used in the single breed analyses: M1 or M2. F_ST_ value of the window including the candidate gene.

^3^ The size of overlapped windows in Mega basepairs (Mb) is reported.

^4^ The number of genes in the identified genome windows is presented.

* Top 70 windows. All other F_ST_ results are from the Top 14 windows containing the reported candidate gene.

F_ST_ values from both methods were reported (M1 and M2). In case relevent gene identified based only one method M1 or M2, one method was reported.

**Table S18** **Statistics for the window-based F_ST_ analyses in the approach based on groups of breeds**

| **Test** | **T_99.8_^1^** | **T_99.0_^2^** | **Mean** | **s.d.^3^** | **Median** | **Min^4^** | **Max^5^** |
| --- | --- | --- | --- | --- | --- | --- | --- |
| Albino breeds (Italian White + Giant White) vs. all other breeds | 0.516 | 0.305 | 0.0805 | 0.063 | 0.063 | 0.000 | 0.627 |
| Silver/greying breeds (Italian Silver + Champagne d’Argent) *vs*. all other breeds | 0.491 | 0.409 | 0.134 | 0.088 | 0.115 | 0.000 | 0.602 |
| Spotted breeds (Checkered Giant + Rhinelander) *vs*. all other breeds | 0.483 | 0.383 | 0.106 | 0.081 | 0.085 | 0.001 | 0.709 |
| Dwarf/small breeds (Coloured Dwarf + Dwarf Lop + Ermine) *vs*. all other breeds | 0.487 | 0.391 | 0.119 | 0.082 | 0.100 | 0.000 | 0.591 |
| Giant breeds (Checkered Giant + Giant Grey + Giant White) *vs*. all other breeds | 0.473 | 0.370 | 0.103 | 0.078 | 0.083 | 0.000 | 0.636 |
| Meat rabbit lines (Italian Silver + Italian Spotted + Italian White) *vs*. all other breeds | 0.451 | 0.317 | 0.087 | 0.067 | 0.069 | 0.001 | 0.573 |

^1^*F*_ST_ values corresponding to the 99.8^th^ percentile; ^2^*F*_ST_ values corresponding to the 99.0^th^ percentile; ^3^Standard deviation; ^4^ Minimum; ^5^ Maximum.

**Table S20 List of relevant genes identified with the F_ST_ single marker-based analysis in the approach based on groups of breeds**

F_ST_ values of the markers at the extreme lower end of the distributions (99.95^th^ percentile) and mapped to genes of interest are presented.

| **Traits/Use** | **Comparison** | **OCU:poition^1^** | **Gene** | **F_ST_^2^** | **Ranking^3^** |
| --- | --- | --- | --- | --- | --- |
| Albino breeds | (Italian white + Giant White) *vs* all other breeds | 1:127563000-127668085 | *TYR* | 0.868 | 1 |
| Silver/graying of coat | (Italian Silver + Champagne d’Argent) *vs* all other breeds | Un513:25550-60684 | *ADNP2* | 0.682 | 26 |
| Dwarf/small body size | (Coloured Dwarf + Dwarf Lop + Ermine) *vs* all other breeds | 2:8357629-8403513, 2:8404807-8620864 | *LCORL, NCAPG* | 0.702 | 5 |
|  |  | Un0030:1681671-2068484 | *NTRK2* | 0.586 | 65 |
|  |  | Un0030:348764-635797 | *FRMD3* | 0.606 | 46 |
| Large body size | (Checkered Giant + Giant Grey + Giant White) *vs* all other breeds | 12:45671510-45793930  12:45977386-46101862 | *BMP5*  *COL21A1* | 0.635 | 64 |
|  |  | 16:82626965-83800640 | *CDH13* | 0.703 | 8 |
|  |  | 2:39527266-39938880 | *MSRA* | 0. 0.663 | 20 |
|  |  | 4:44715759-44848250 | *HMGA2* | 0.709 | 7 |
| Meat production | (Italian Silver + Italian Spotted + Italian White) vs. all other breeds |  |  |  |  |
|  |  | 2:158326199-158338803 | *MRPL33* | 0.566 | 5 |
|  |  | 15:58389801-59653895 | *CCSER1* | 0.579 | 17 |
|  |  | 9:49249318-49360593 | *PTPN2* | 0.581 | 45 |

^1^ Position of the candidate gene, in basepairs, on the *O. cuniculus* reference genome (OryCun2.0). OCU = *Oryctolagus cuniculus* chromosome.

^2^ F_ST_ value of top significant marker mapped to the candidate gene.

^3^ Ranking of the top SNP mapped with the gene of interest.

**Table S26 Gene enrichment analysis**

Gene sets (99.0^th^ percentile) related to the group-based F_ST_ analyses were tested for over-represented biological features.

| **Comparison^1^** | **Database** | **Term** | **Overlap^2^** | ***p*-value^3^** | **Genes^4^** | **Windows^5^** |
| --- | --- | --- | --- | --- | --- | --- |
| Spotted | GWAS-catalog | Response to platinum-based neoadjuvant chemotherapy in cervical cancer | 10/22 | 1.71E-20 | *NECAB2, MBTPS1, HSDL1, HSBP1, TAF1C, DNAAF1, OSGIN1, CDH13, SLC38A8, ADAD2* | 2 |
| Spotted | GWAS-catalog | Response to bronchodilator in chronic obstructive pulmonary disease (change in FEV1) | 4/38 | 5.17E-05 | *KCNG4, HSBP1, CDH13, MLYCD* | 2 |
| Silver | GO-BP | QRS duration | 3/55 | 2.40E-02 | *RNF11, CDKN2C, FAF1* | 2 |

^1^Details are given in Table S2; ^2^Number of genes of the input set over the number of genes annotated with the tested term; ^3^Adjusted *p*-value; ^4^Genes of the input set annotated with the tested term; ^5^Number of windows encompassing the input annotated genes.
