# Supplementary material for: Genomic diversity and signatures of selection in meat and fancy rabbit breeds based on high-density marker data
Source: Genet Sel Evol. 2022 Jan 21;54:3. doi: 10.1186/s12711-022-00696-9 (PMC8780294; doi:10.1186/s12711-022-00696-9)
Supplement: Supplementary file 2 — Additional file 2: Figure S1. Window-based Neighbor Joining tree. Figure S2. Multidimensional scaling plot. The first three components are provided. Figure S3. Scree plot used to identify the number of principal components that describe well the population structure of the investigated rabbit breeds. The plot displays in decreasing order the percentage of variance explained by each principal component. Figure S4. Manhattan plots of the PCAdapt analysis. Each dot represents a 350-kb genome window. The red line identifies the threshold value (0.1 Bonferroni corrected P-value). Unassembled scaffolds are not reported. Figure S5. Genome regions carrying signatures of selection (99.8th percentile; expanded windows) identified in the studied breeds. Only the assembled autosomes are presented and unassembled scaffolds are not reported. Figure S6. Manhattan plots of the genome-wide FST analyses based on Method 1 (M1). Each dot represents a 350-kb genome window. The blue line identifies the threshold value (99.8th percentile of the distribution). Unassembled scaffolds are not reported. Figure S7. Manhattan plots of the genome-wide FST analyses based on Method 2 (M2). Each dot represents a 350-kb genome window. The blue line identifies the threshold value (99.8th percentile of the distribution). Unassembled scaffolds are not reported. [file 12711_2022_696_MOESM2_ESM.doc]

**Figure S1. Window-based Neighbor Joining tree.**

**
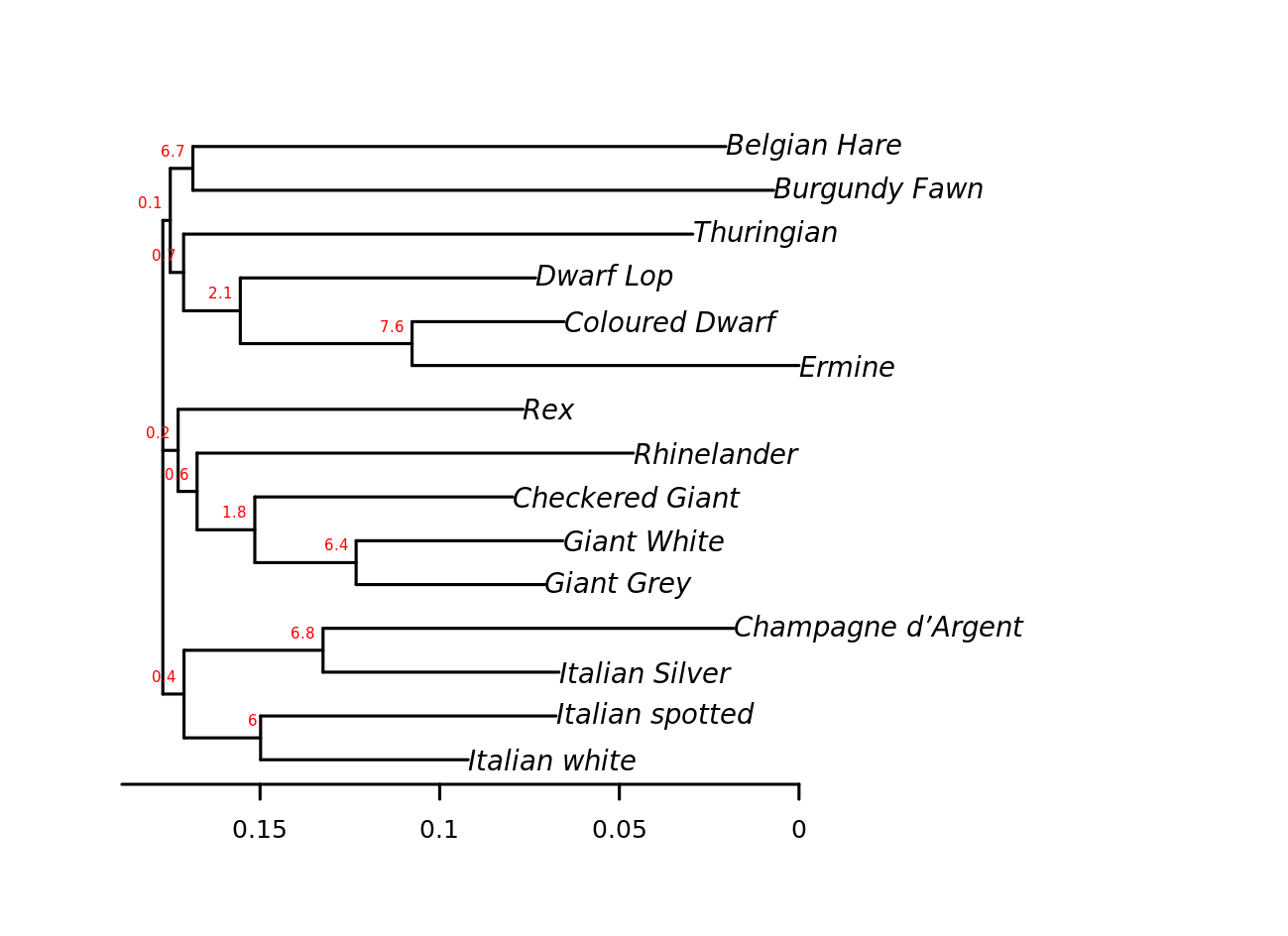
**

**Figure S2. Multidimensional scaling plot.** The first three components are provided.

**
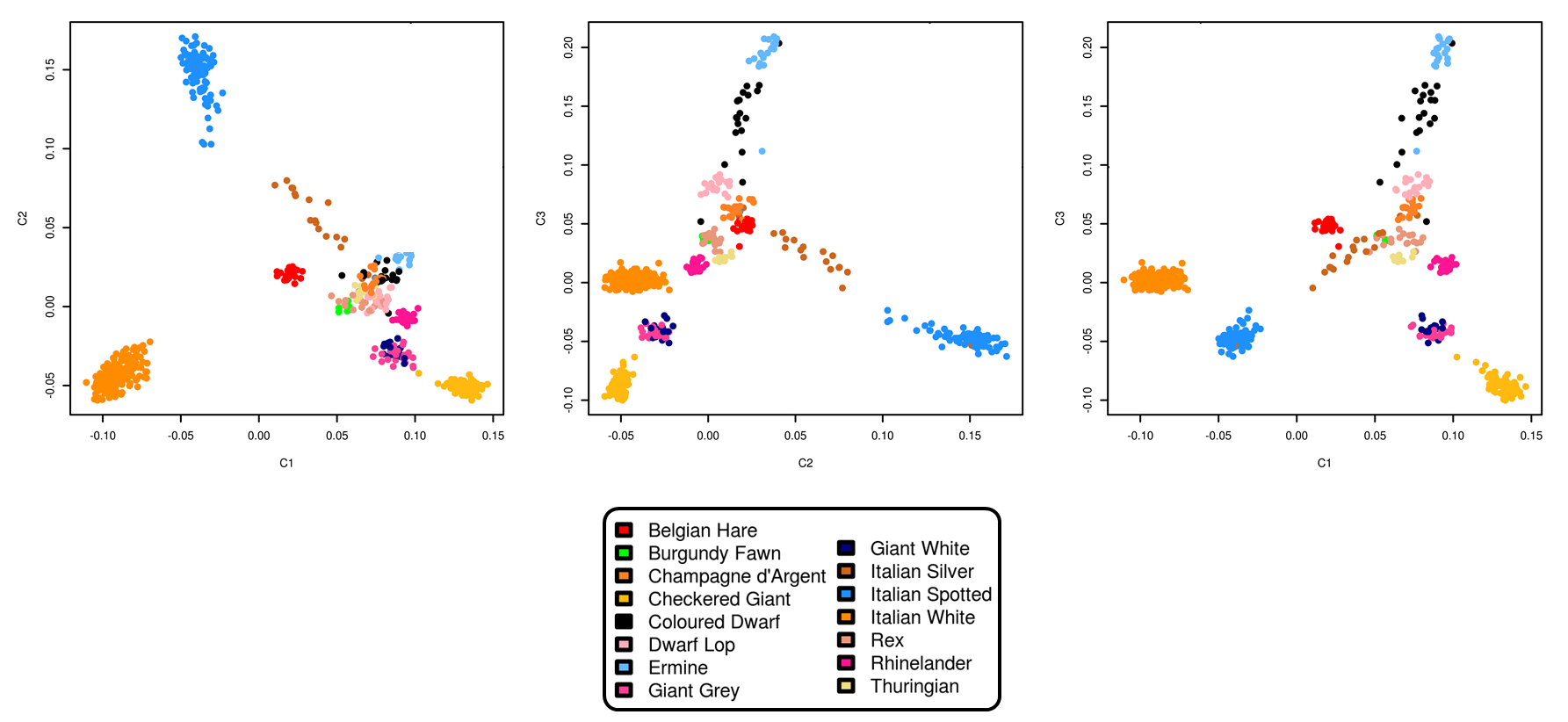
**

**Figure S3. Scree plot used to identify the number of principal components that well describe the population structure of the investigated rabbit breeds.** The plot displays in decreasing order the percentage of variance explained by each principal component.

**
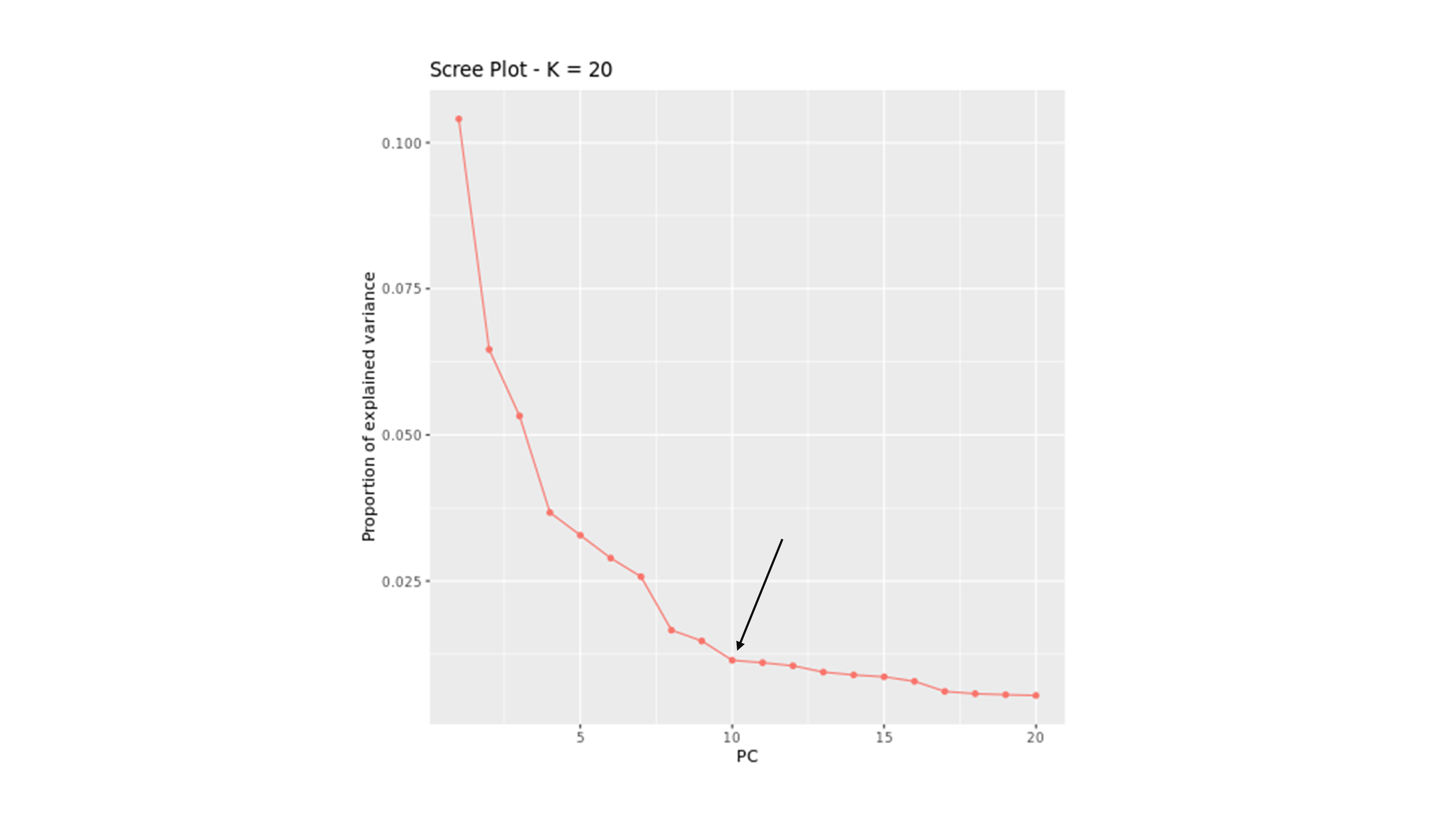
**

**Figure S4. Manhattan plots of the *PCAdapt* analysis.** Each dot represents a 350-kb genome window. The red line identifies the threshold value (0.1 Bonferroni corrected *P*-value). Unassembled scaffolds are not reported.


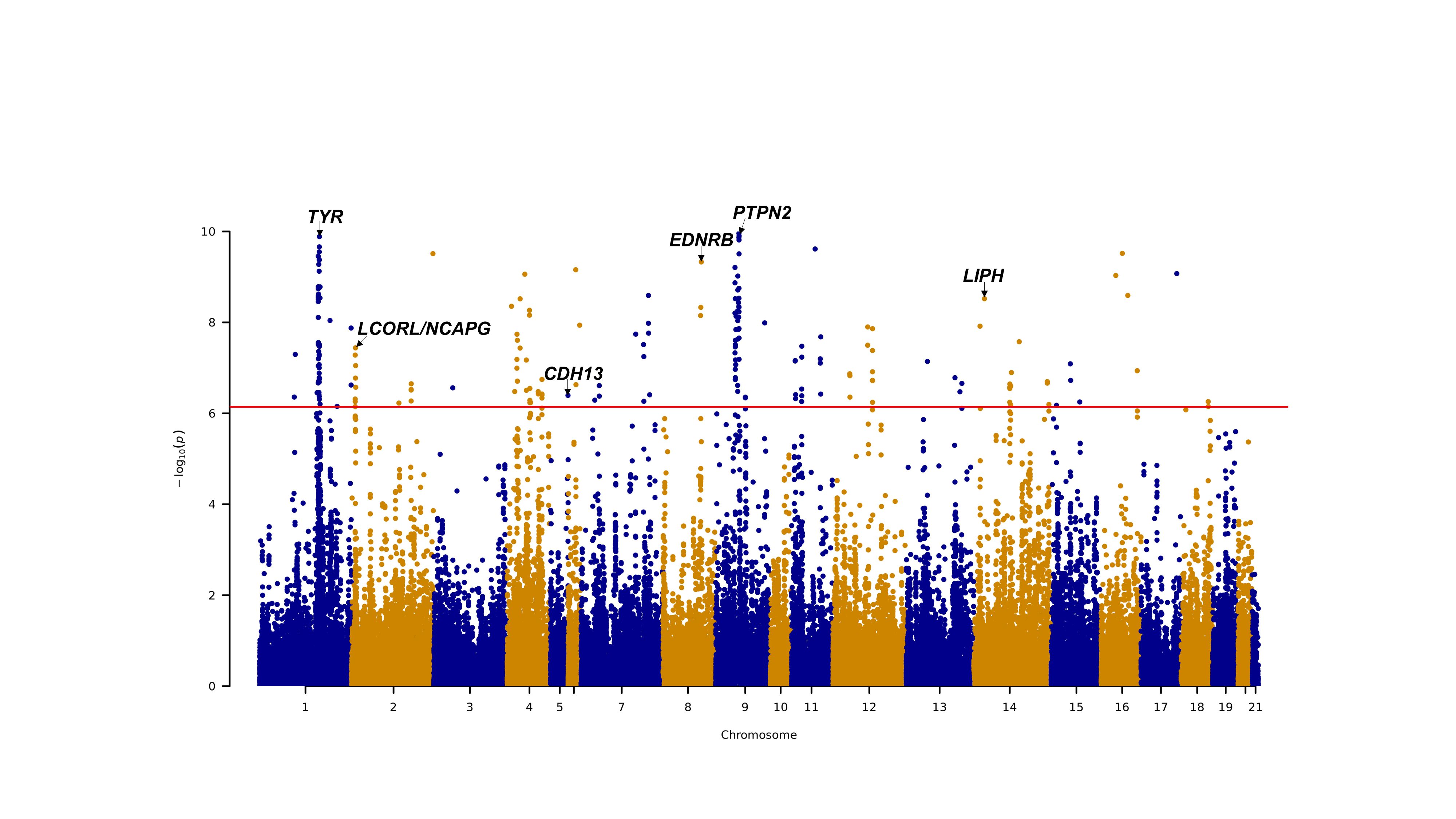


**Figure S5. Genome regions carrying signatures of selection (99.8^th^ percentile; expanded windows) identified in the studied breeds.** Only the assembled autosomes are presented and unassembled scaffolds are not reported.

**
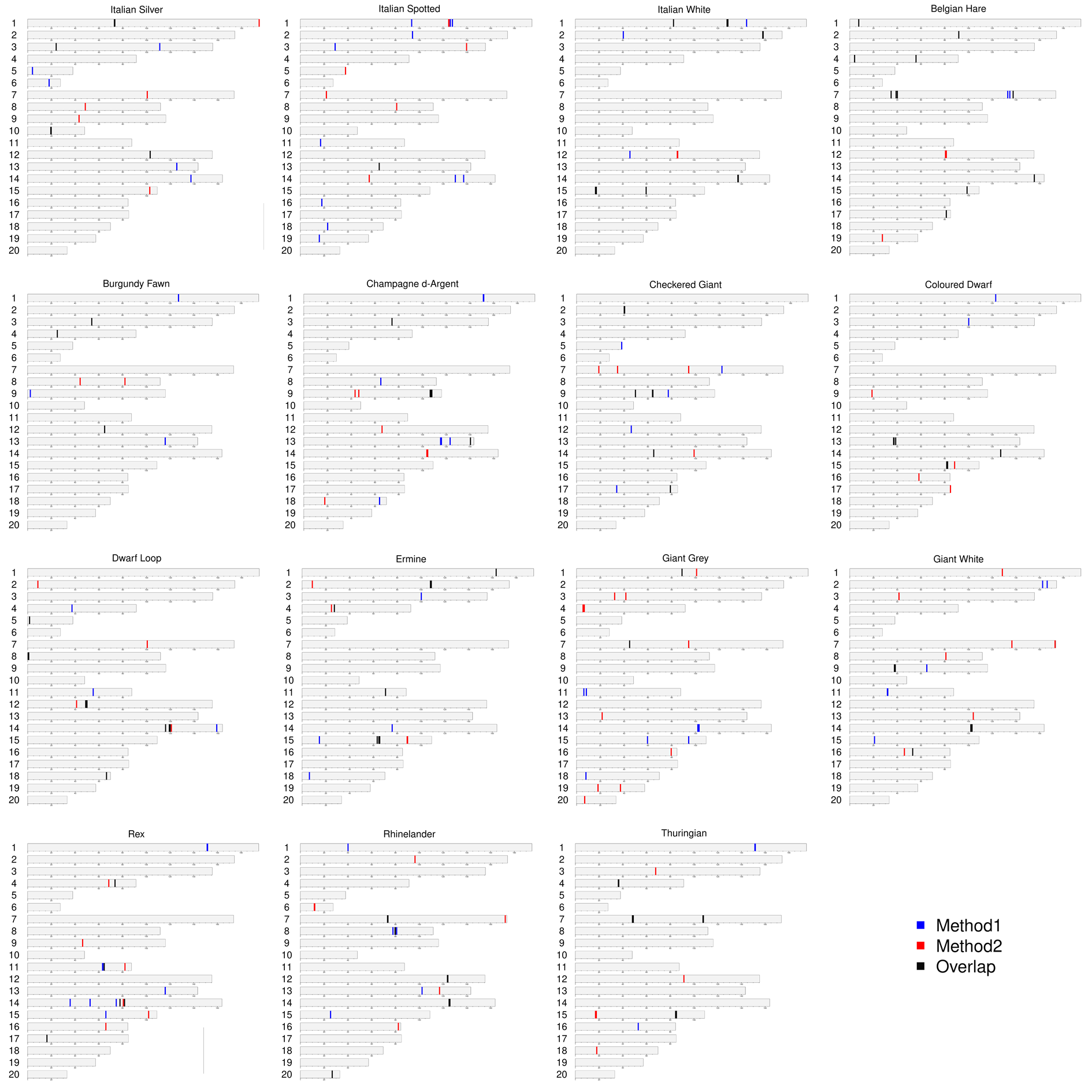
**

**Figure S6. Manhattan plots of the genome-wide F_ST_ analyses based on Method 1 (M1).** Each dot represents a 350-kb genome window. The blue line identifies the threshold value (99.8^th^ percentile of the distribution). Unassembled scaffolds are not reported.

**
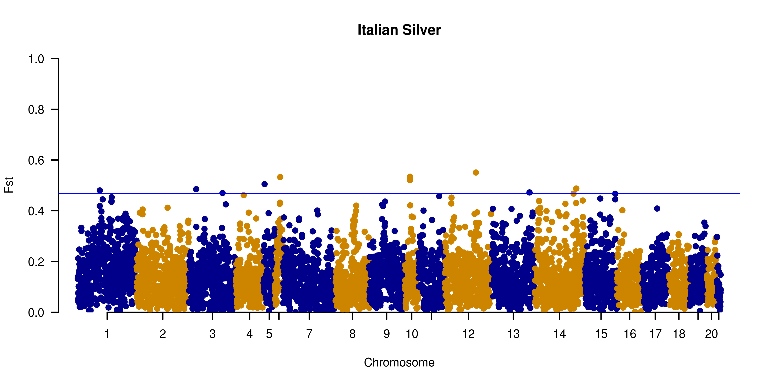

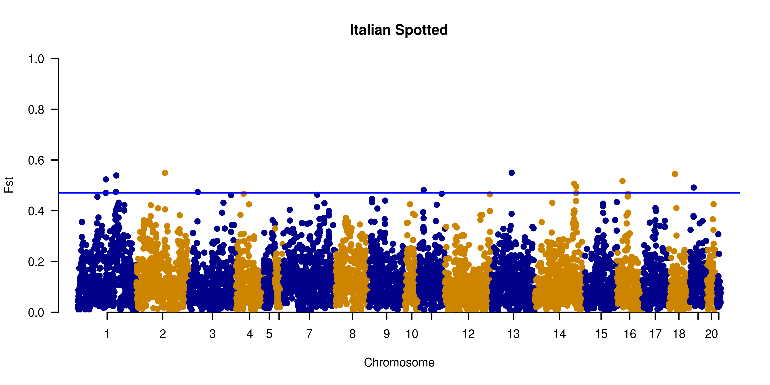

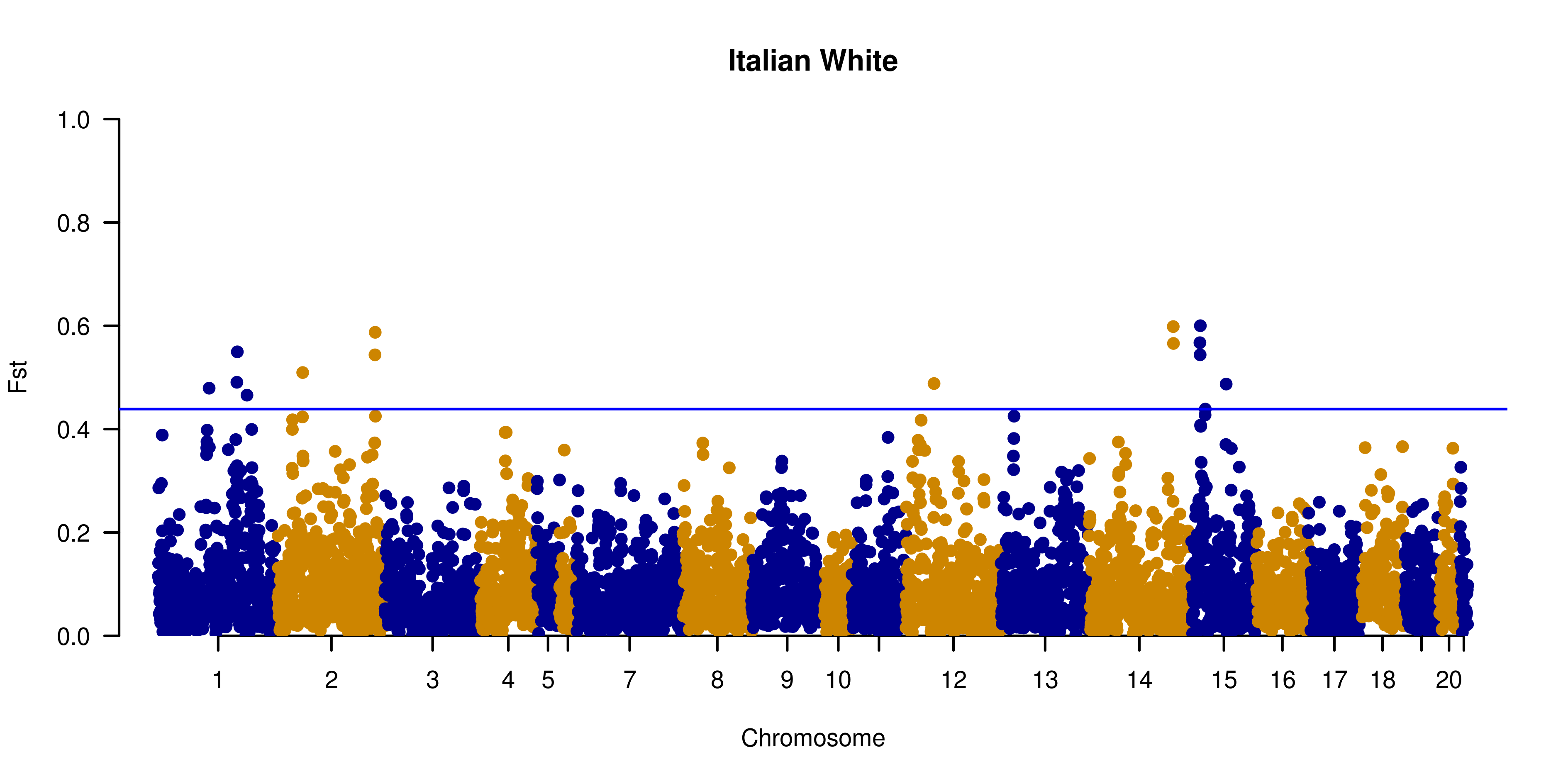

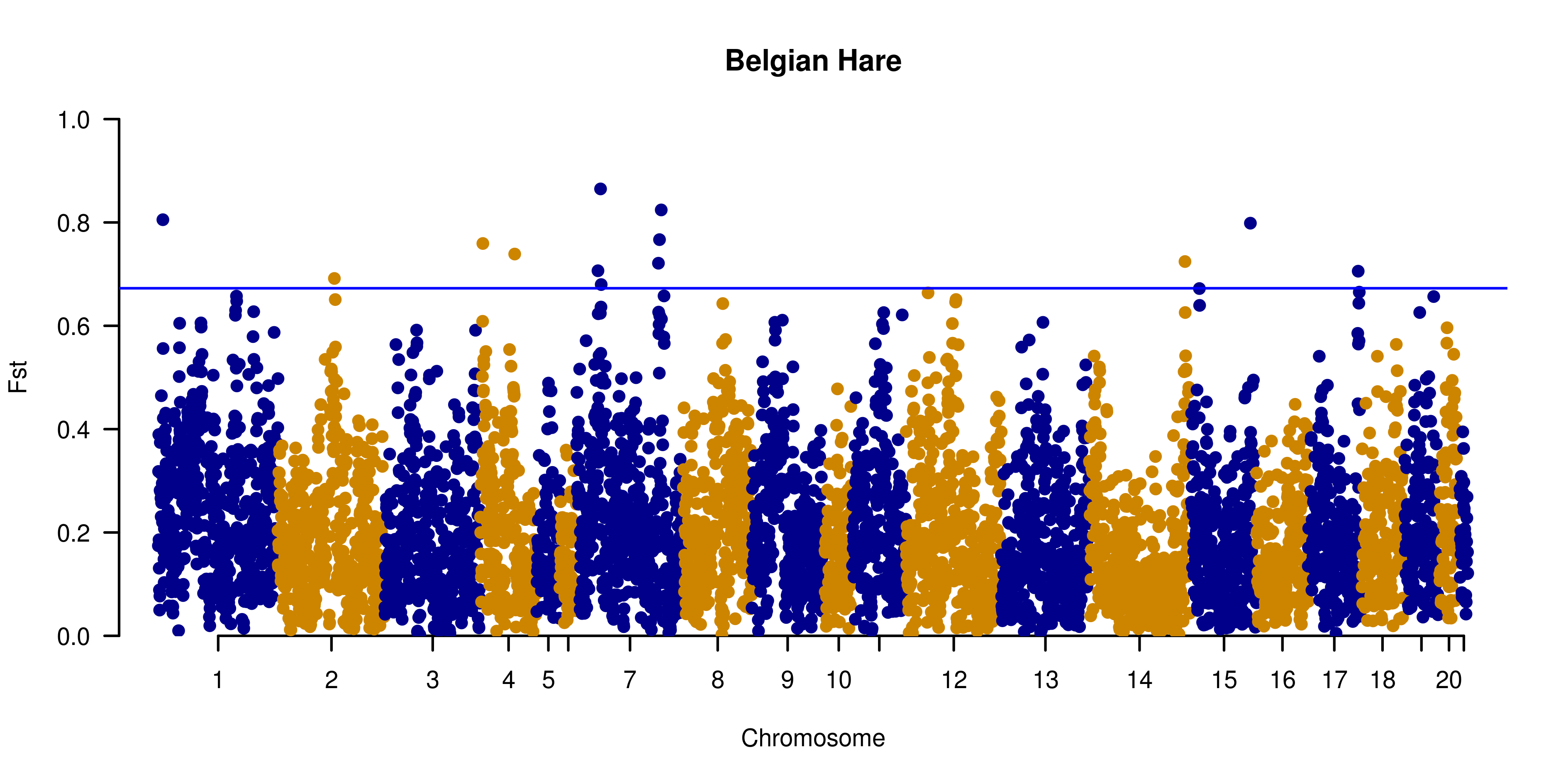

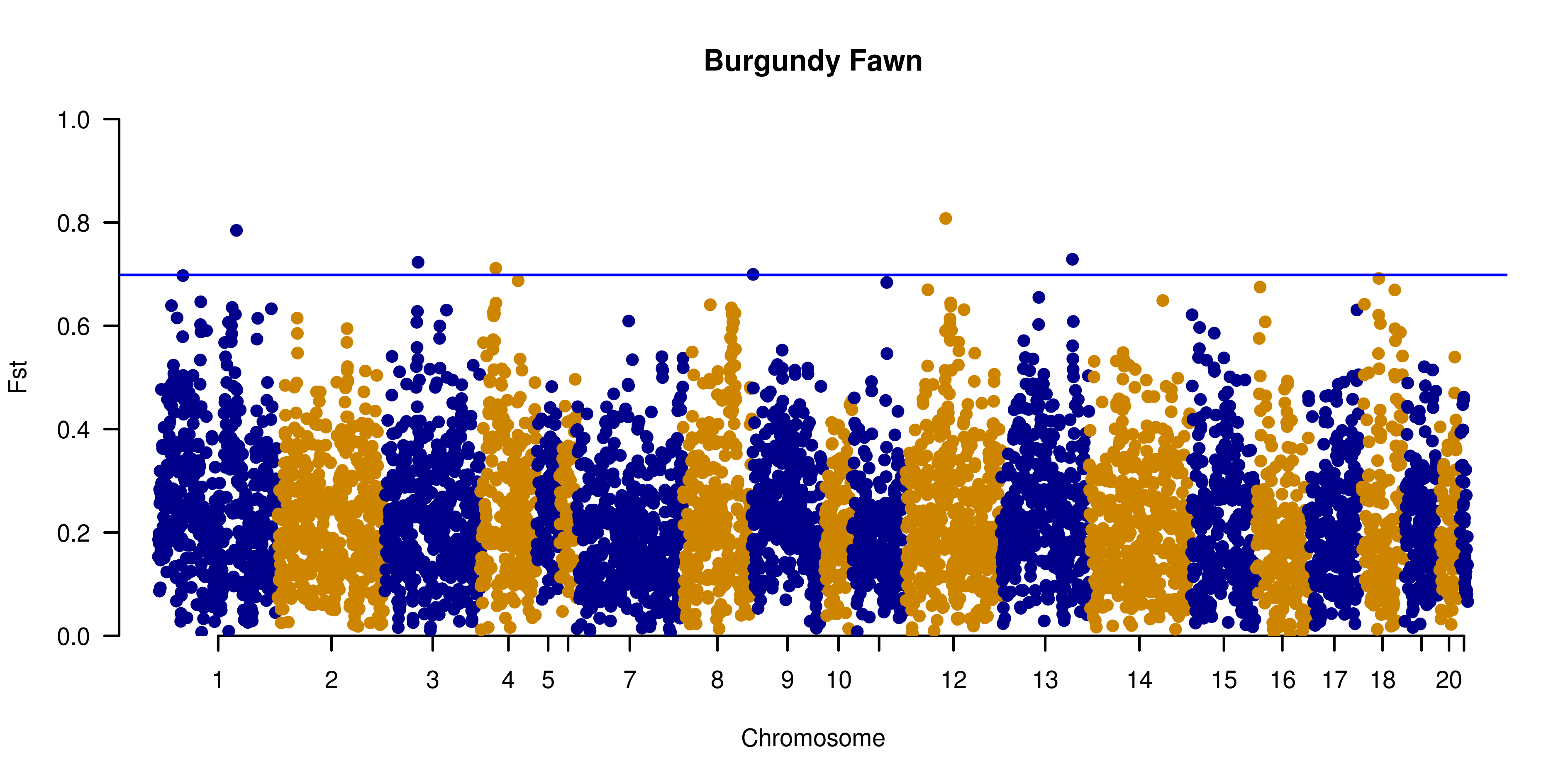

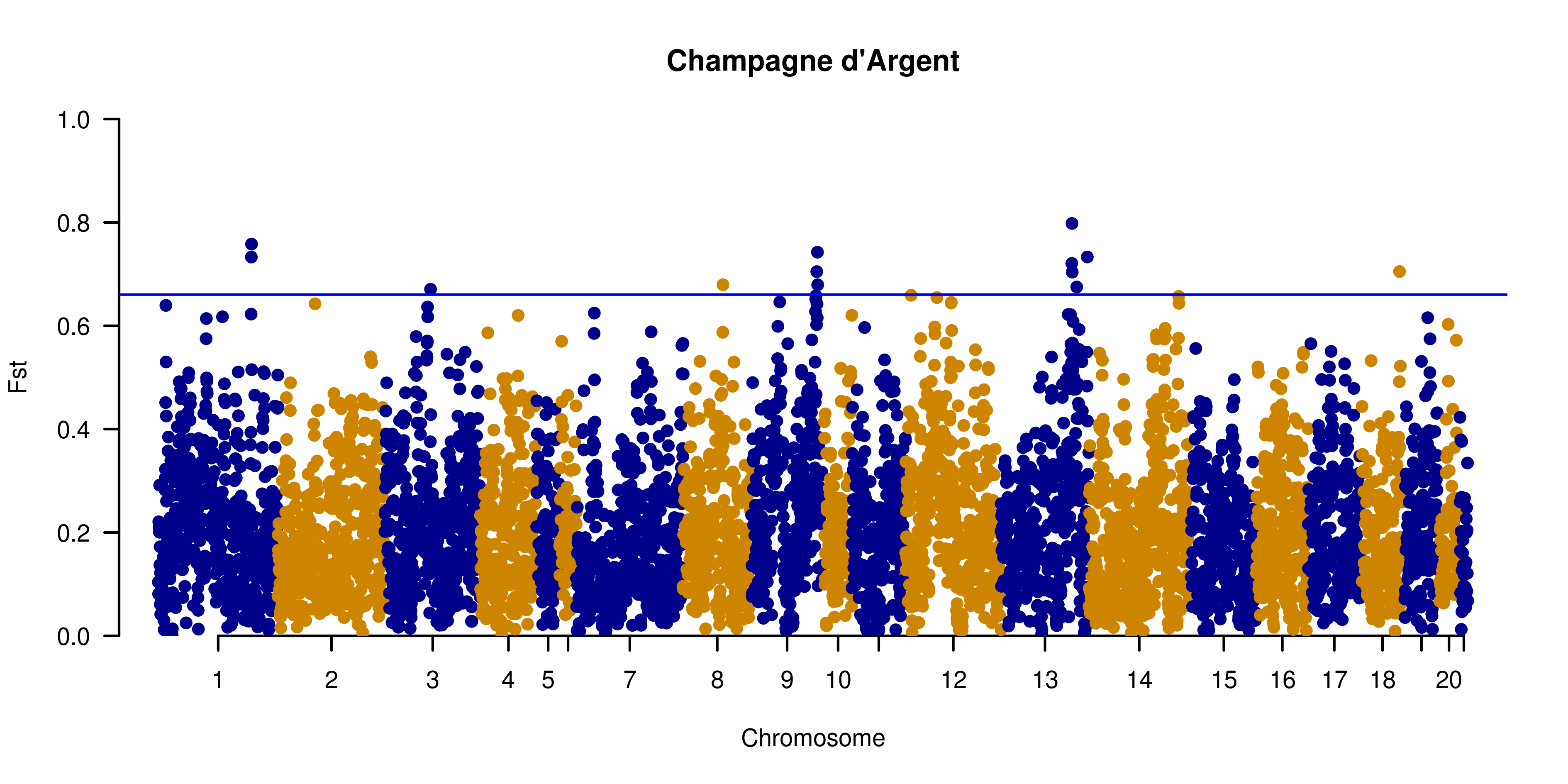

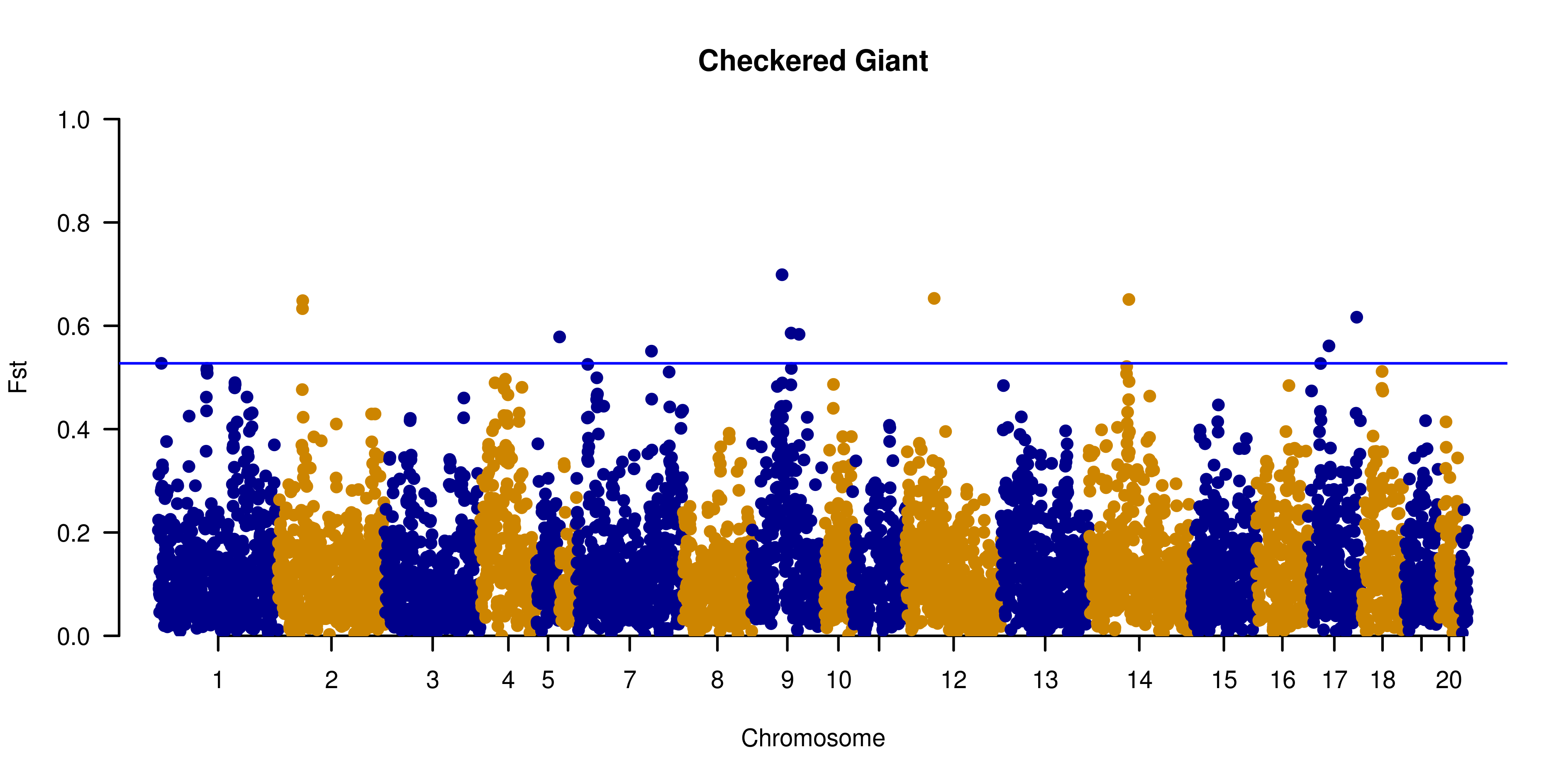

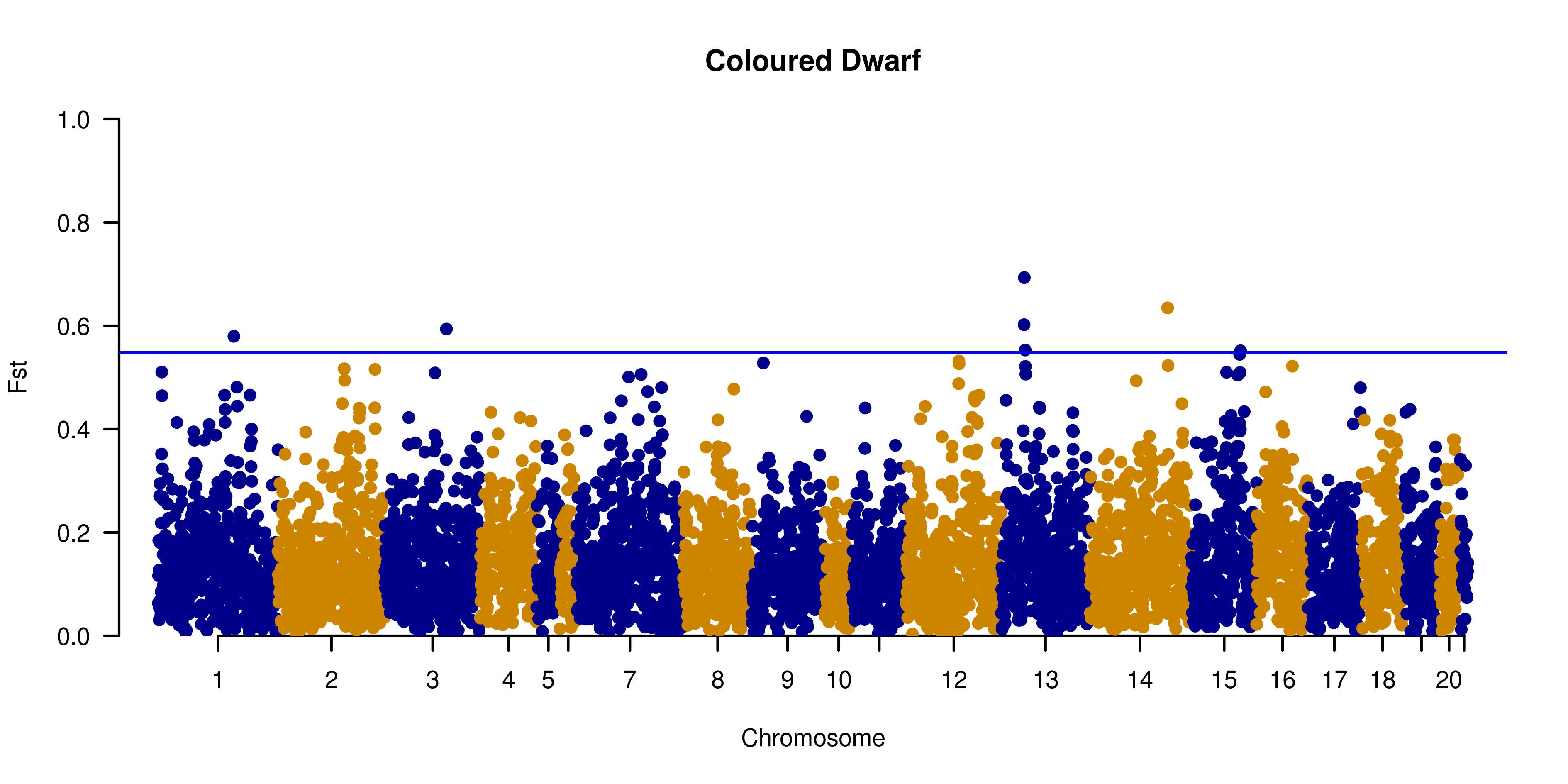
**

**
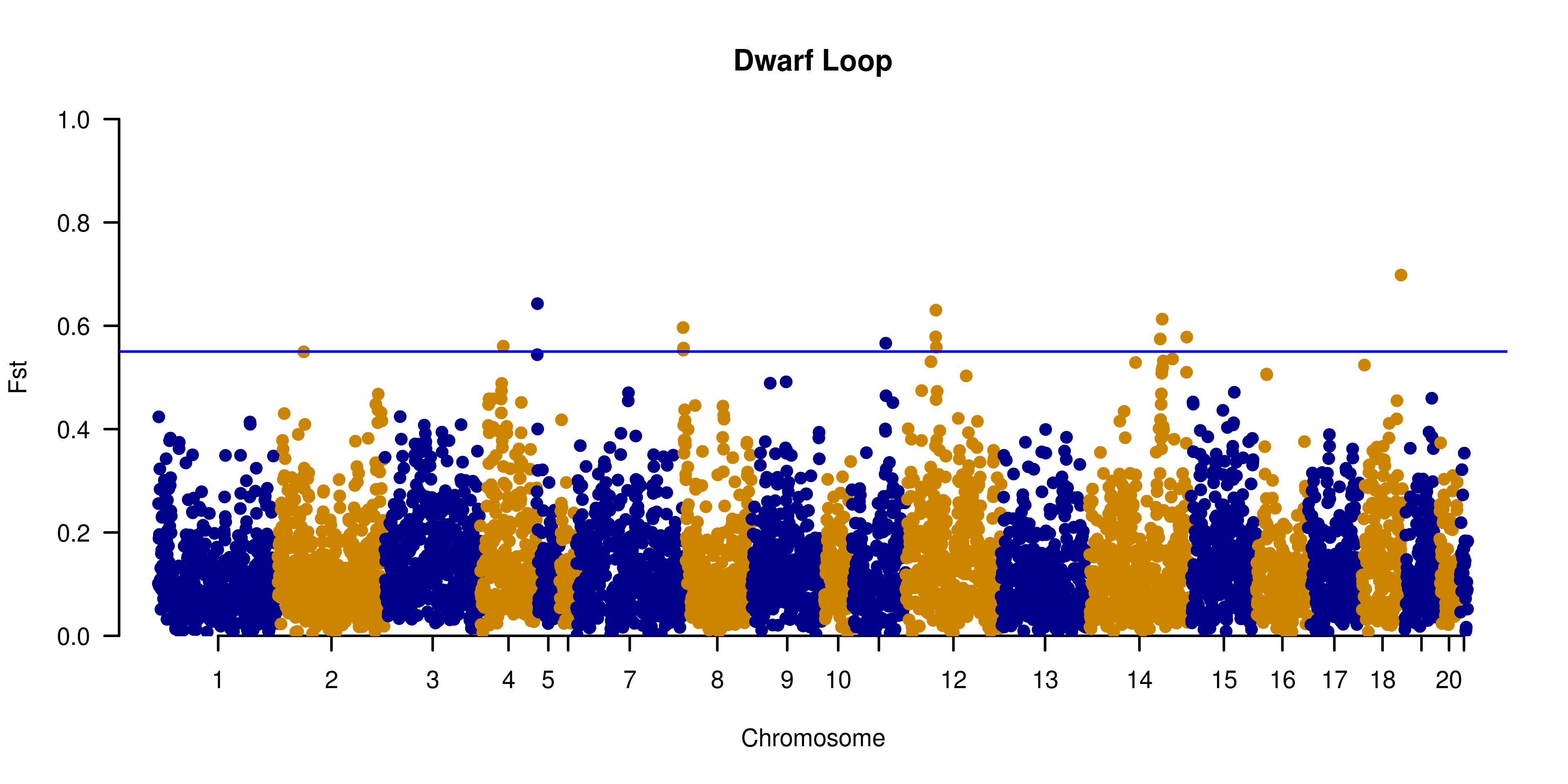

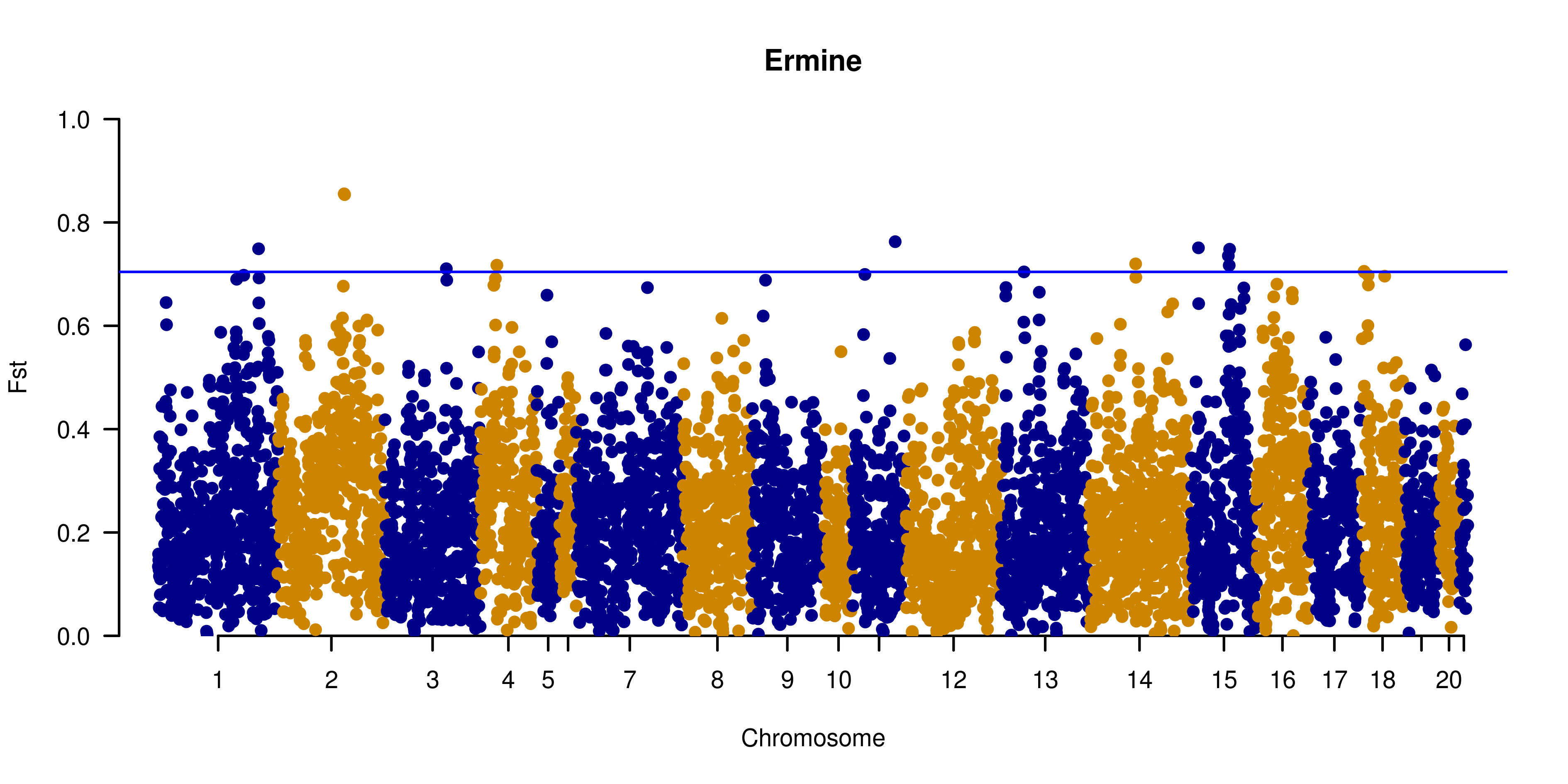
**

**
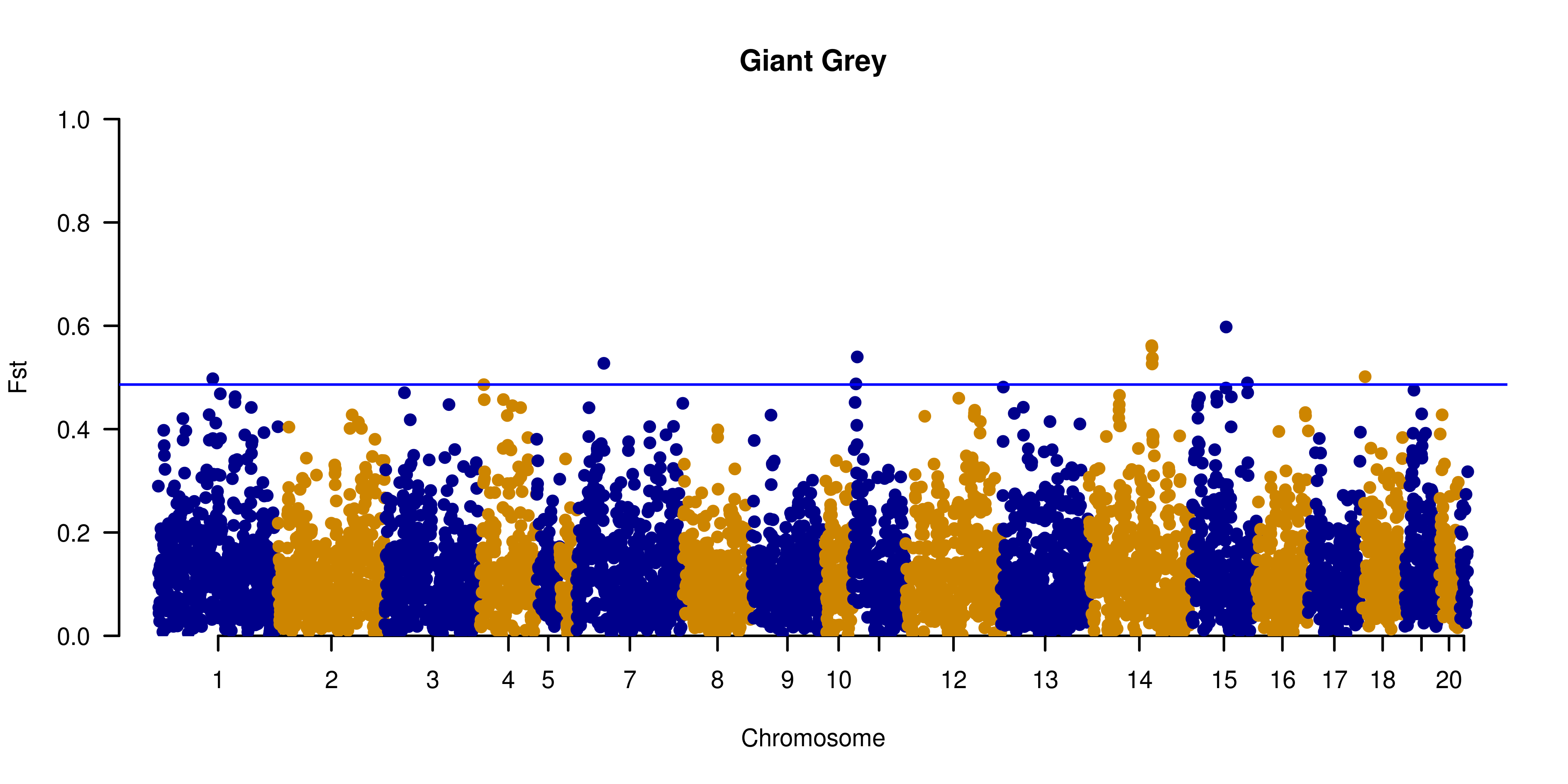

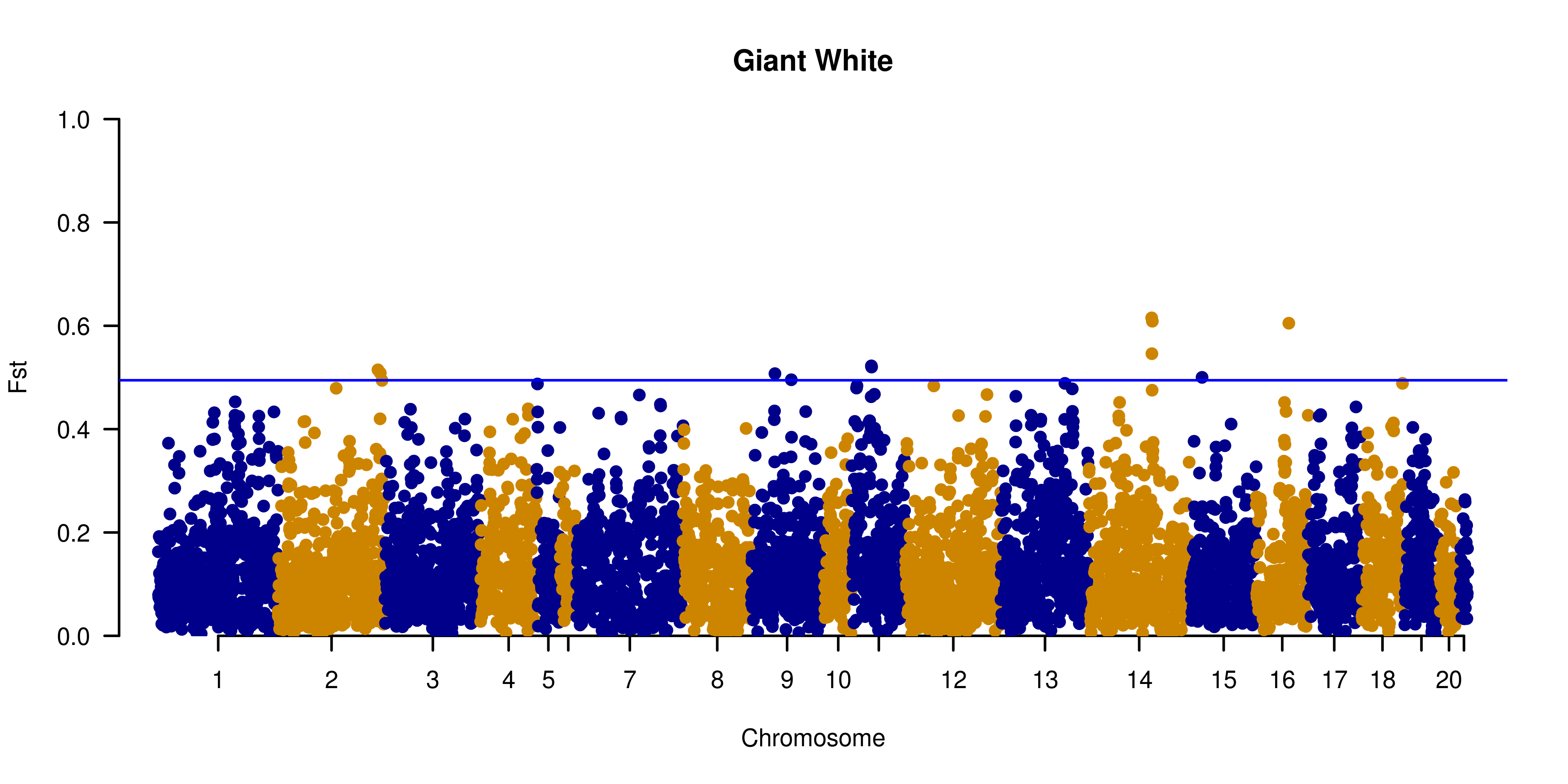
**

**
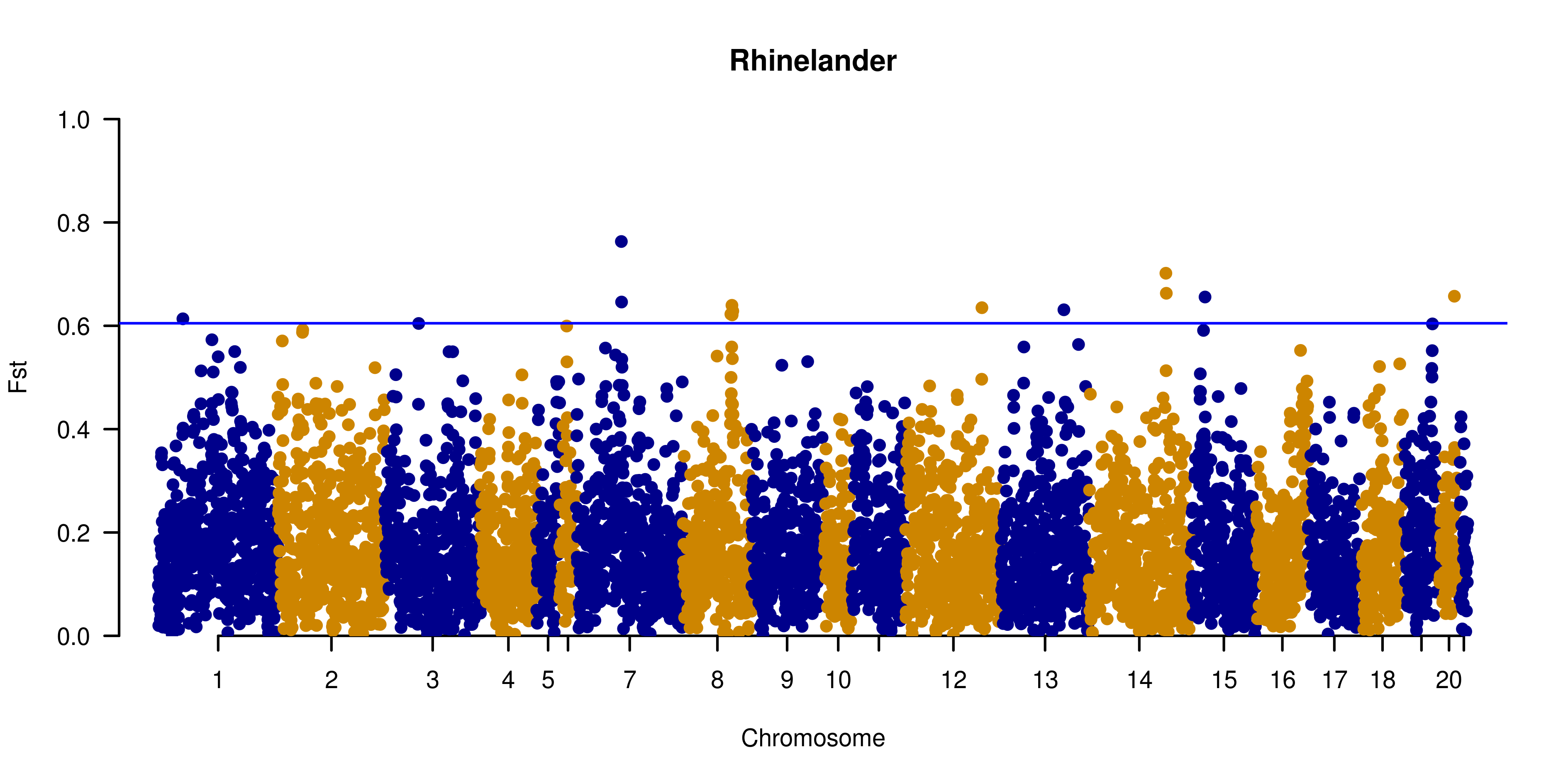
**


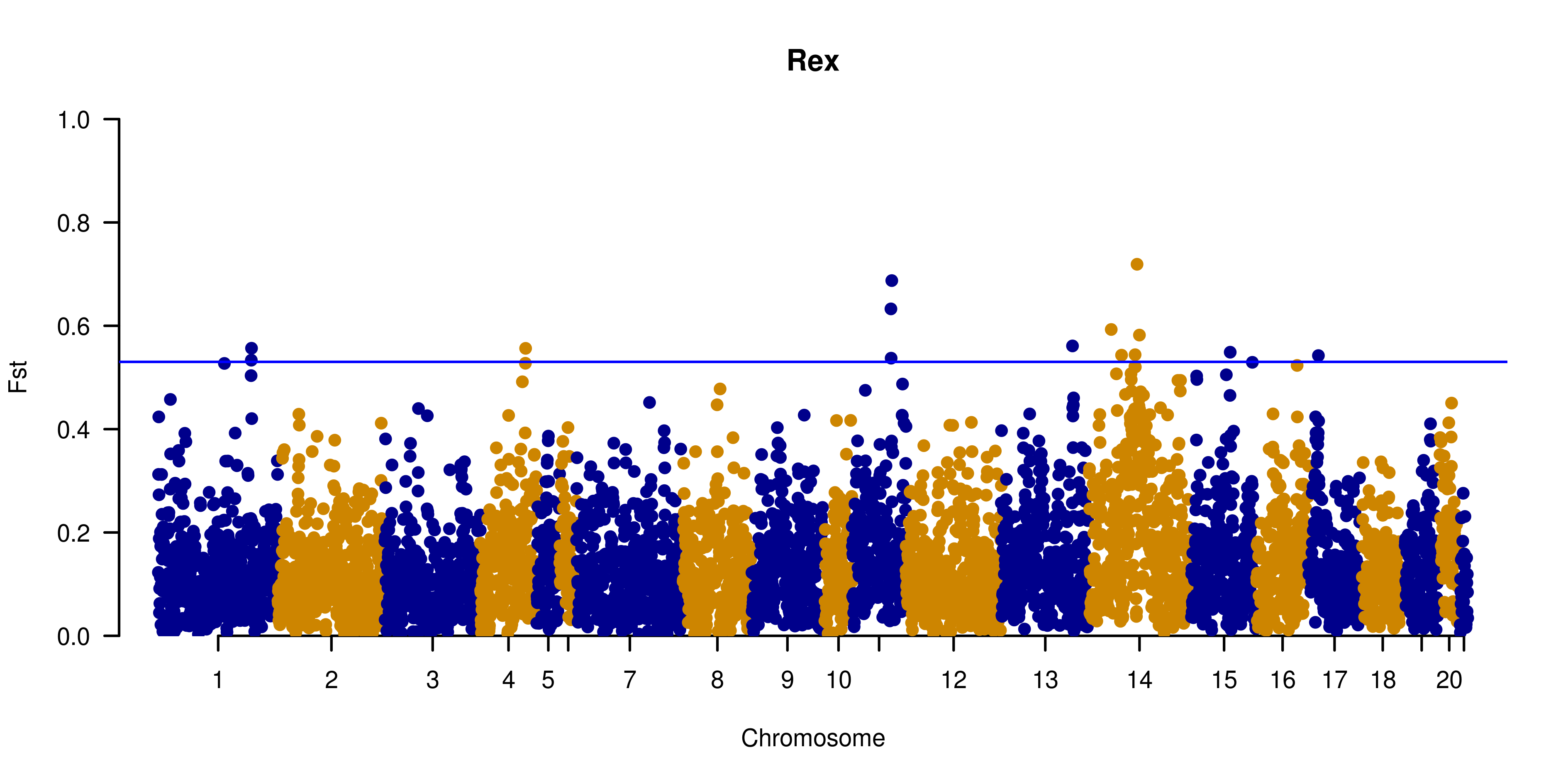


**
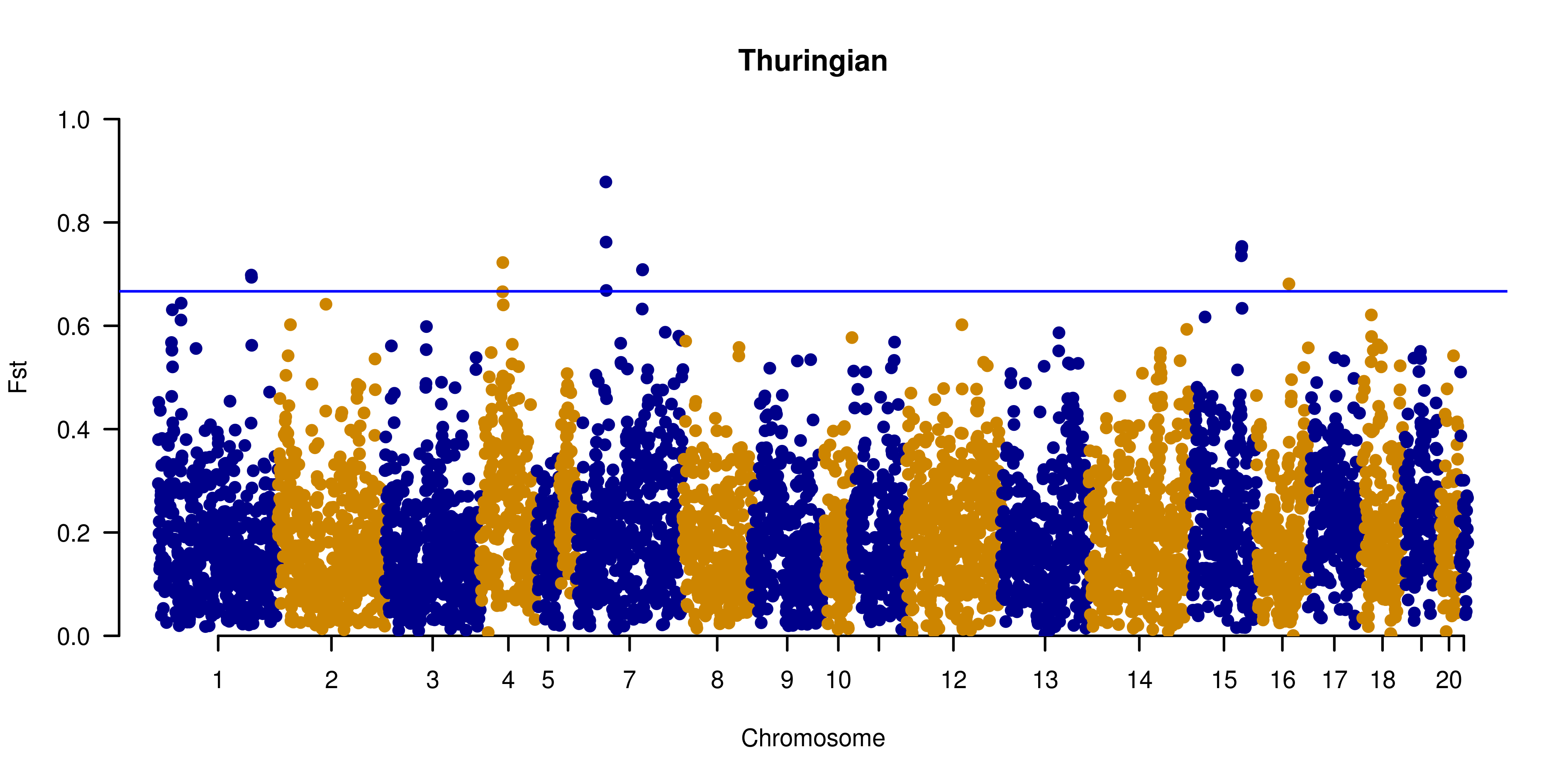
**


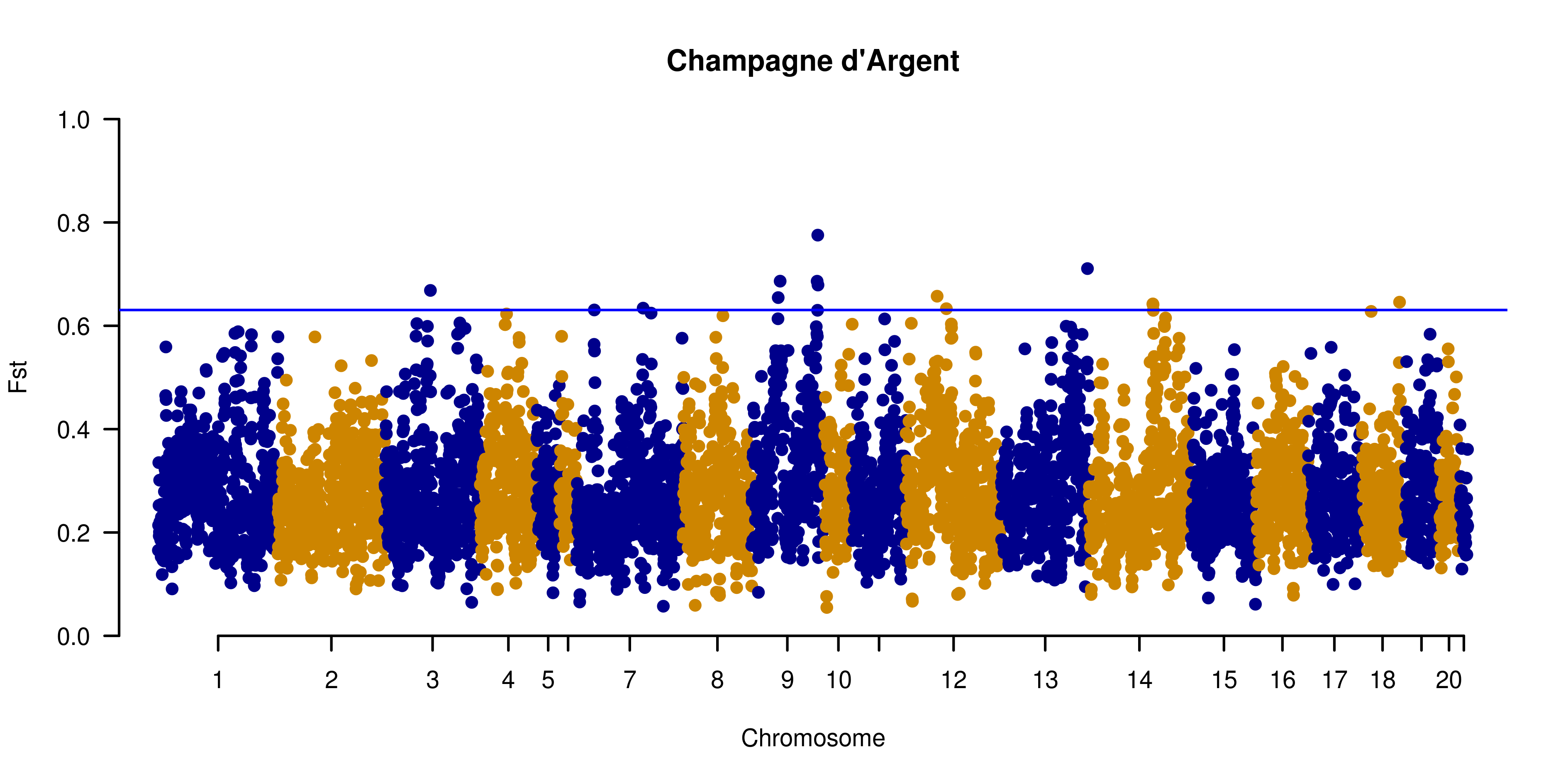

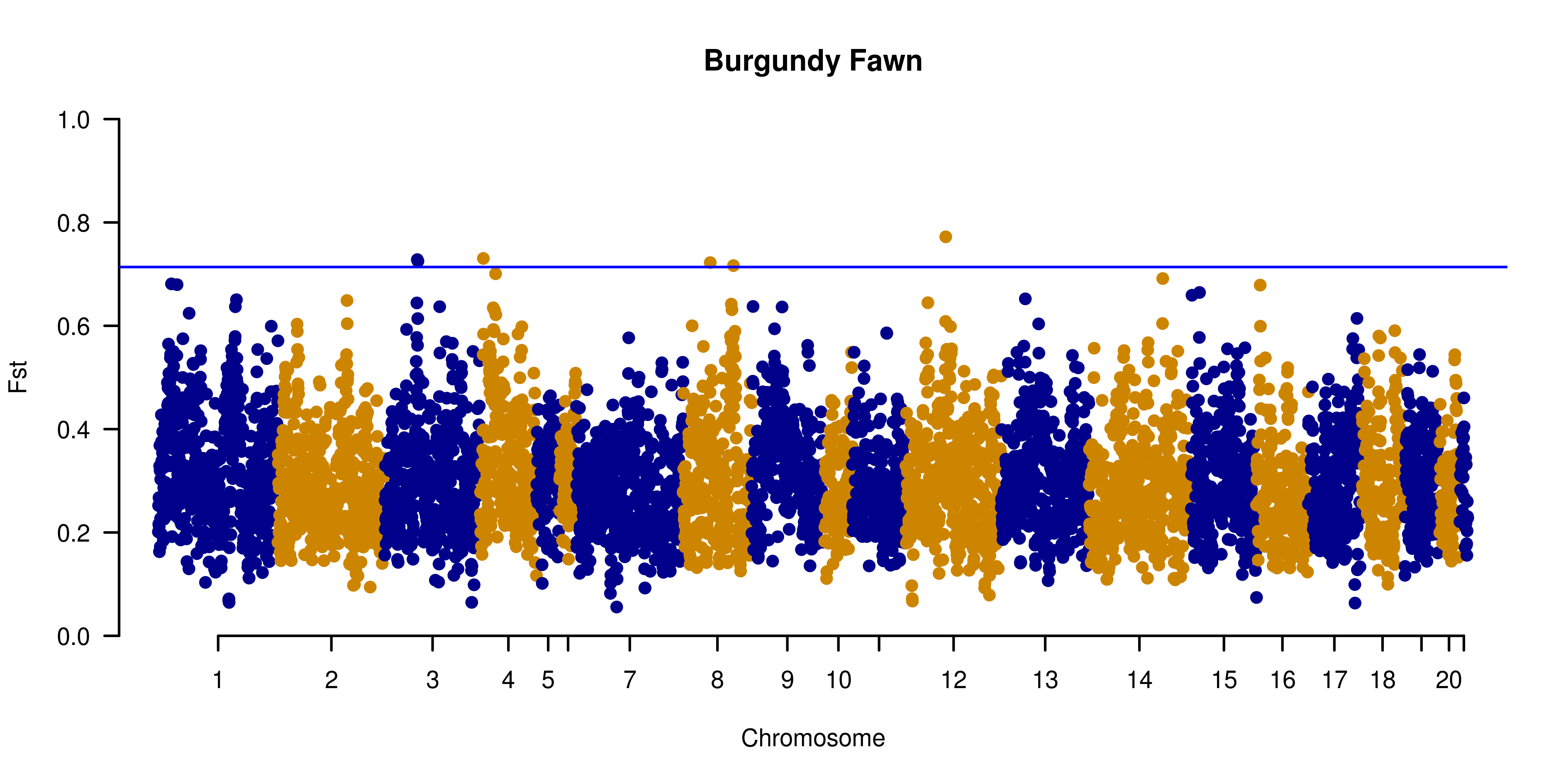

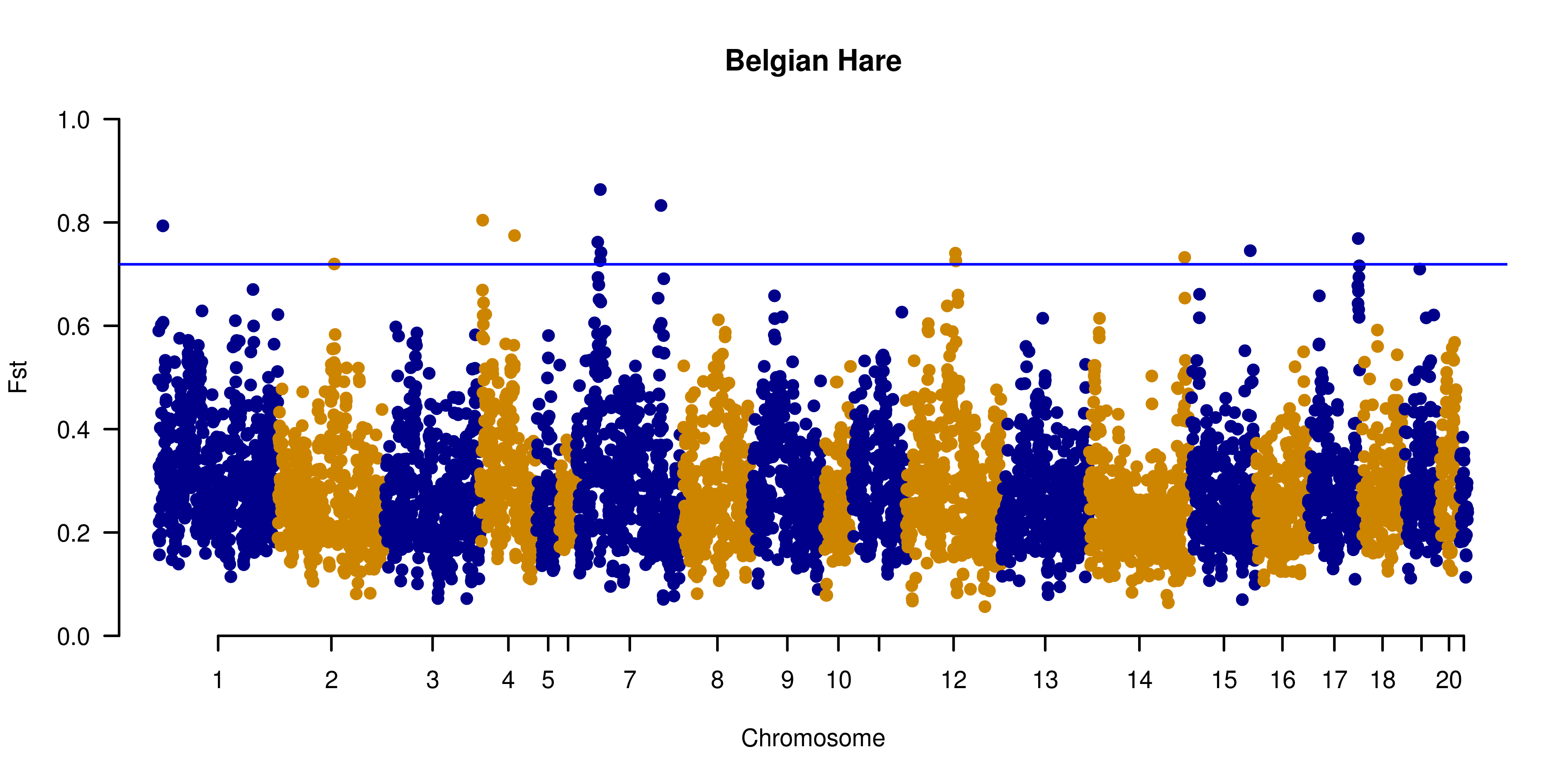
**Figure S7. Manhattan plots of the genome-wide F_ST_ analyses based on Method 2 (M2).** Each dot represents a 350-kb genomic window. The blue line identifies the threshold value (99.8^th^ percentile of the distribution). Unassembled scaffolds are not reported.


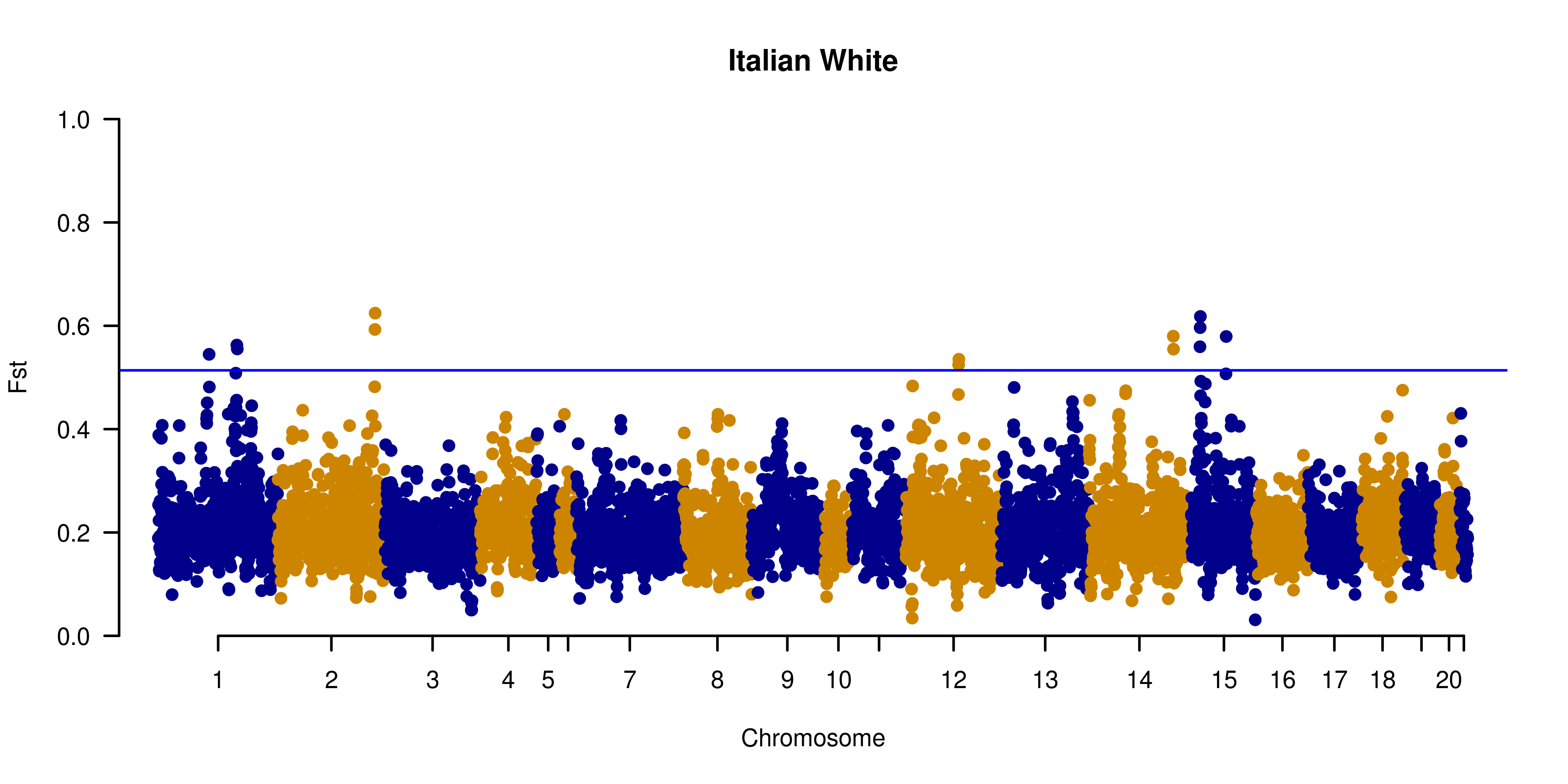

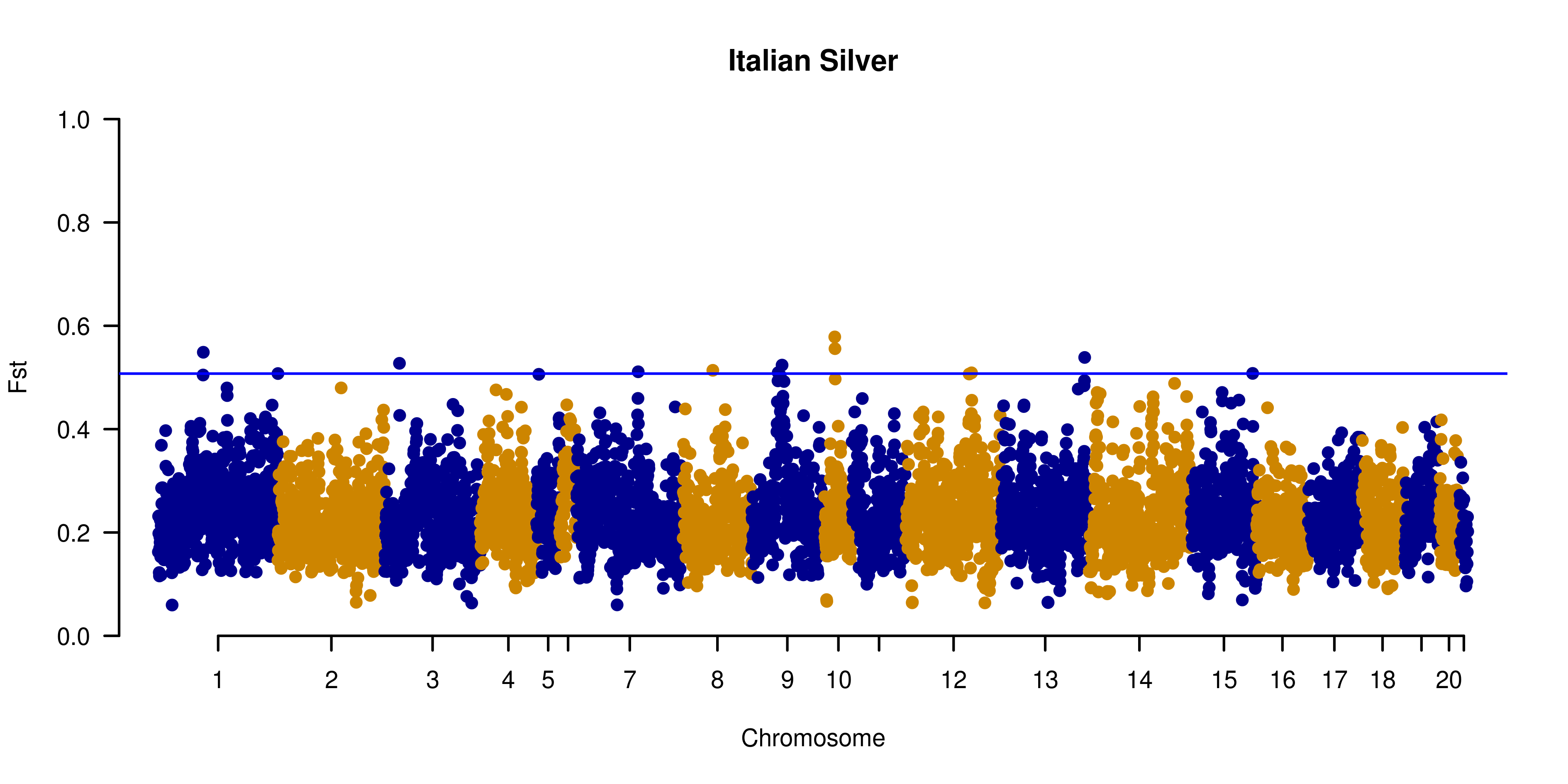

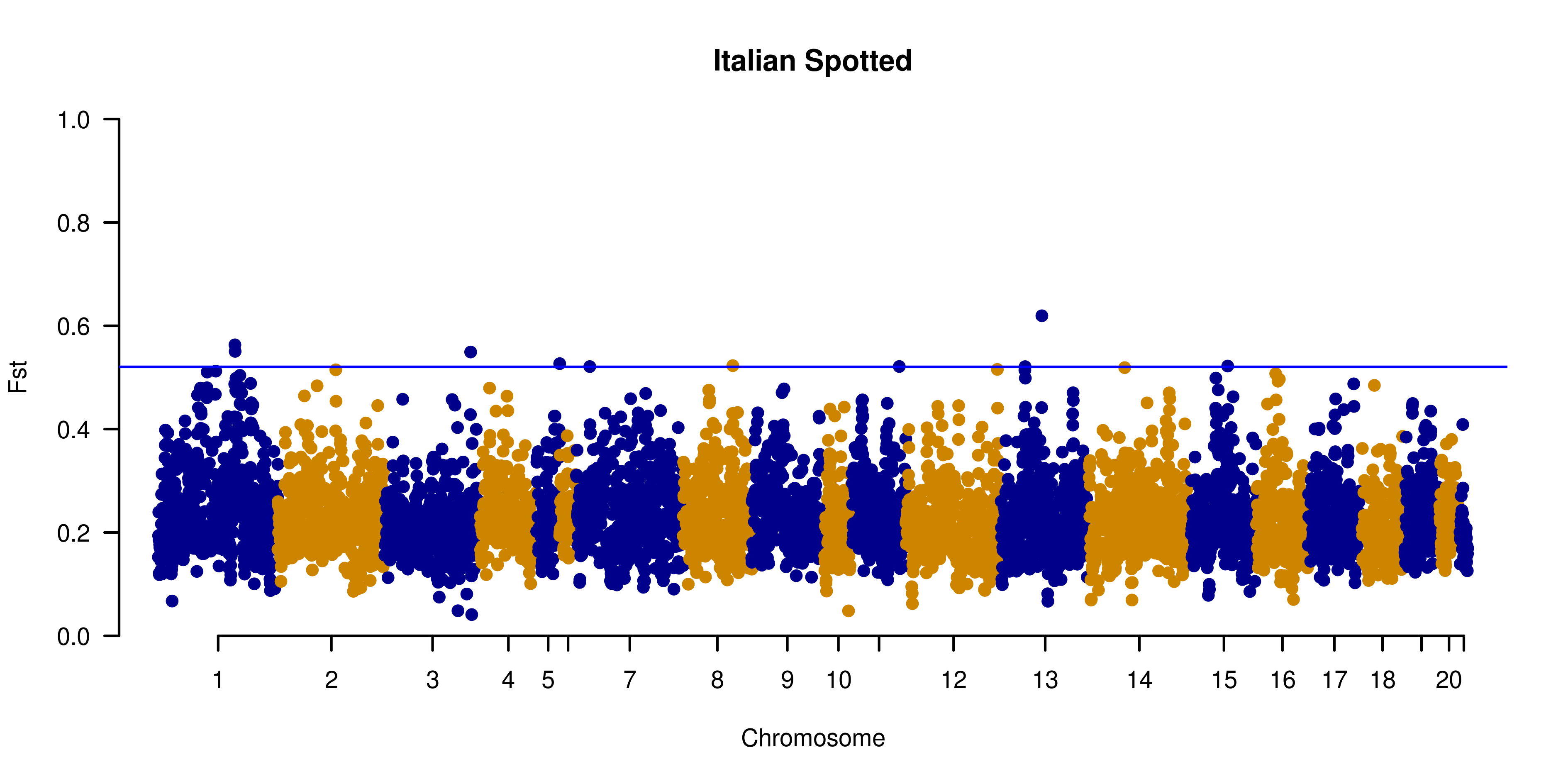

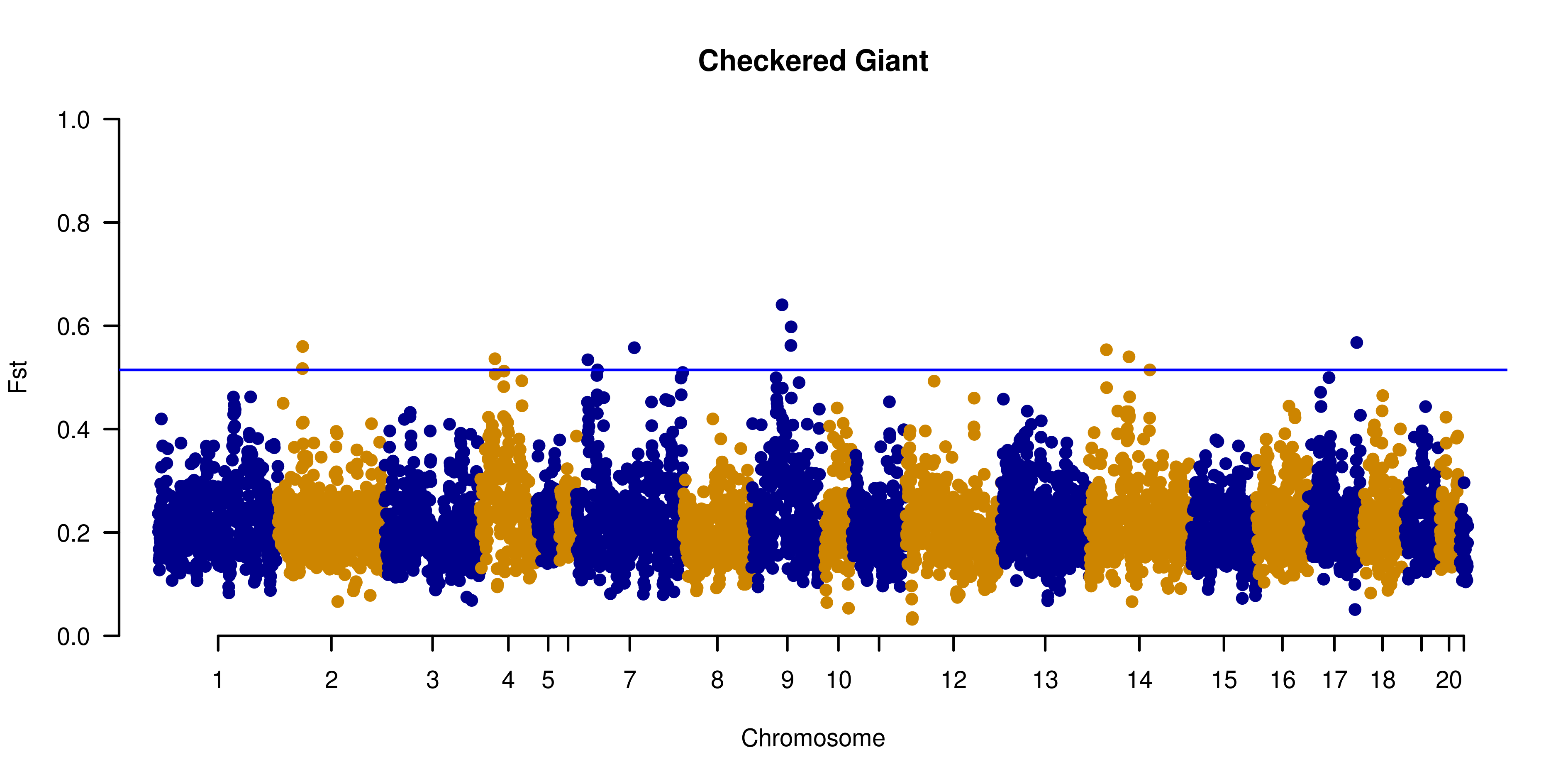

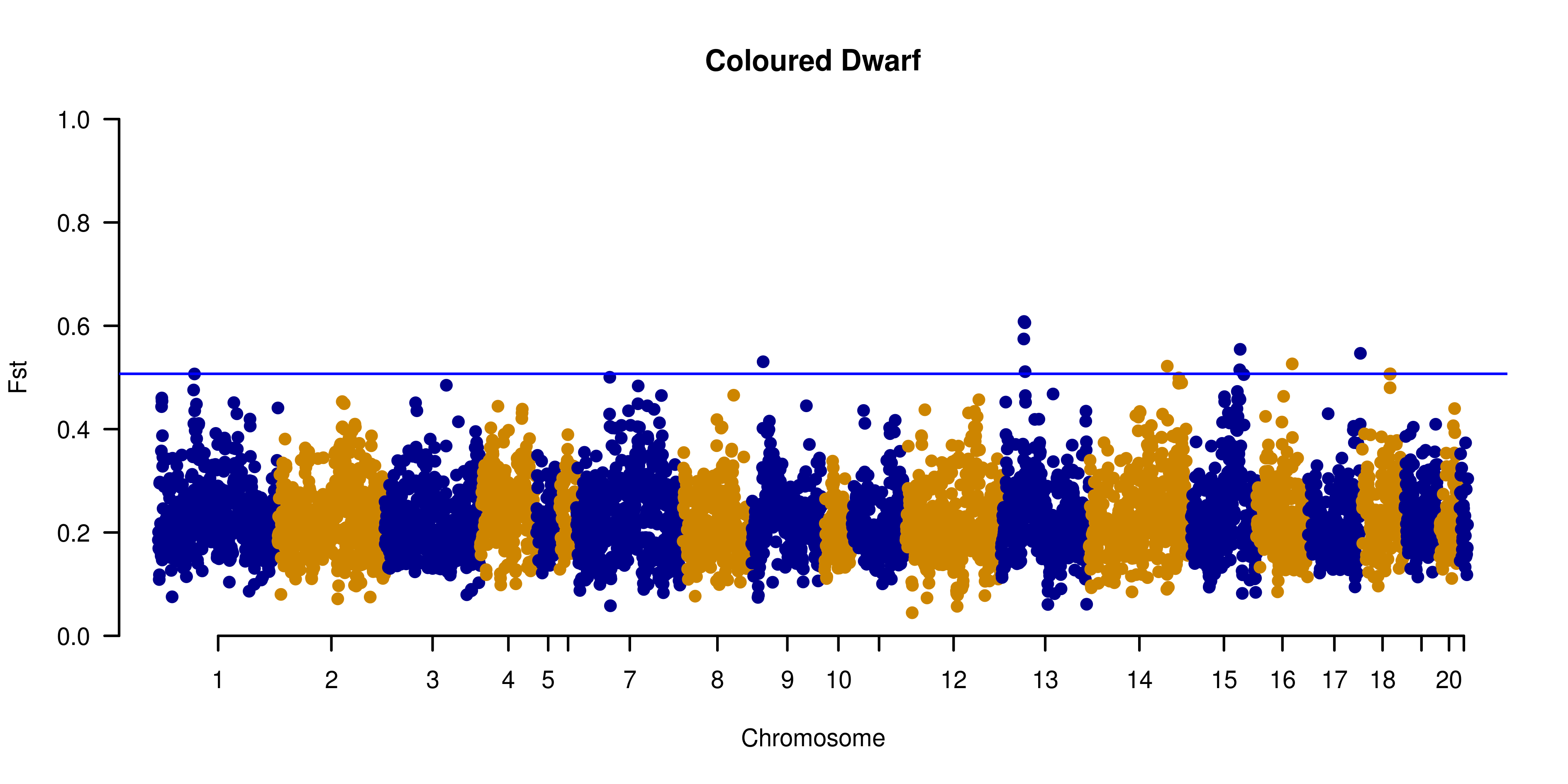


**
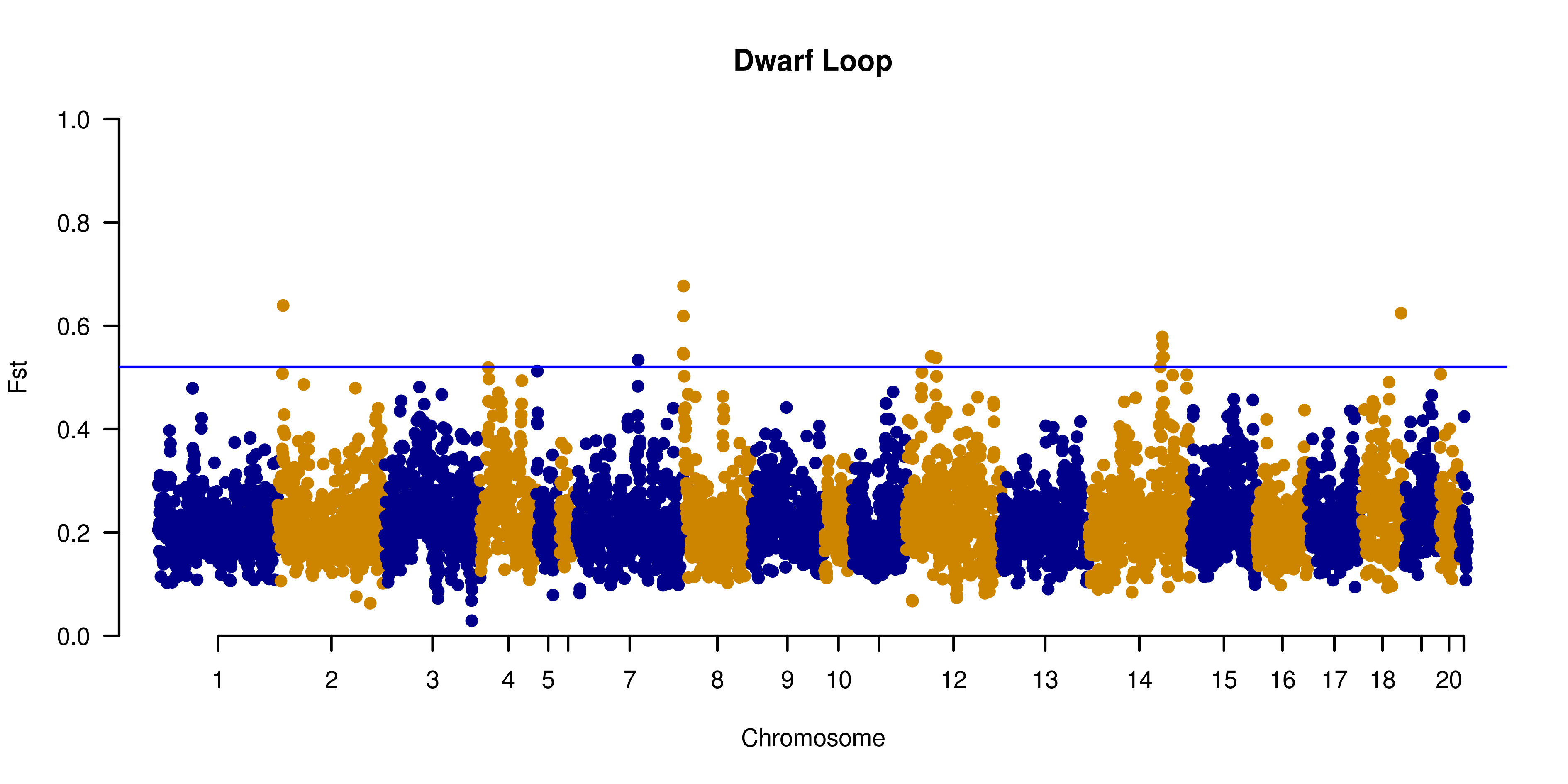

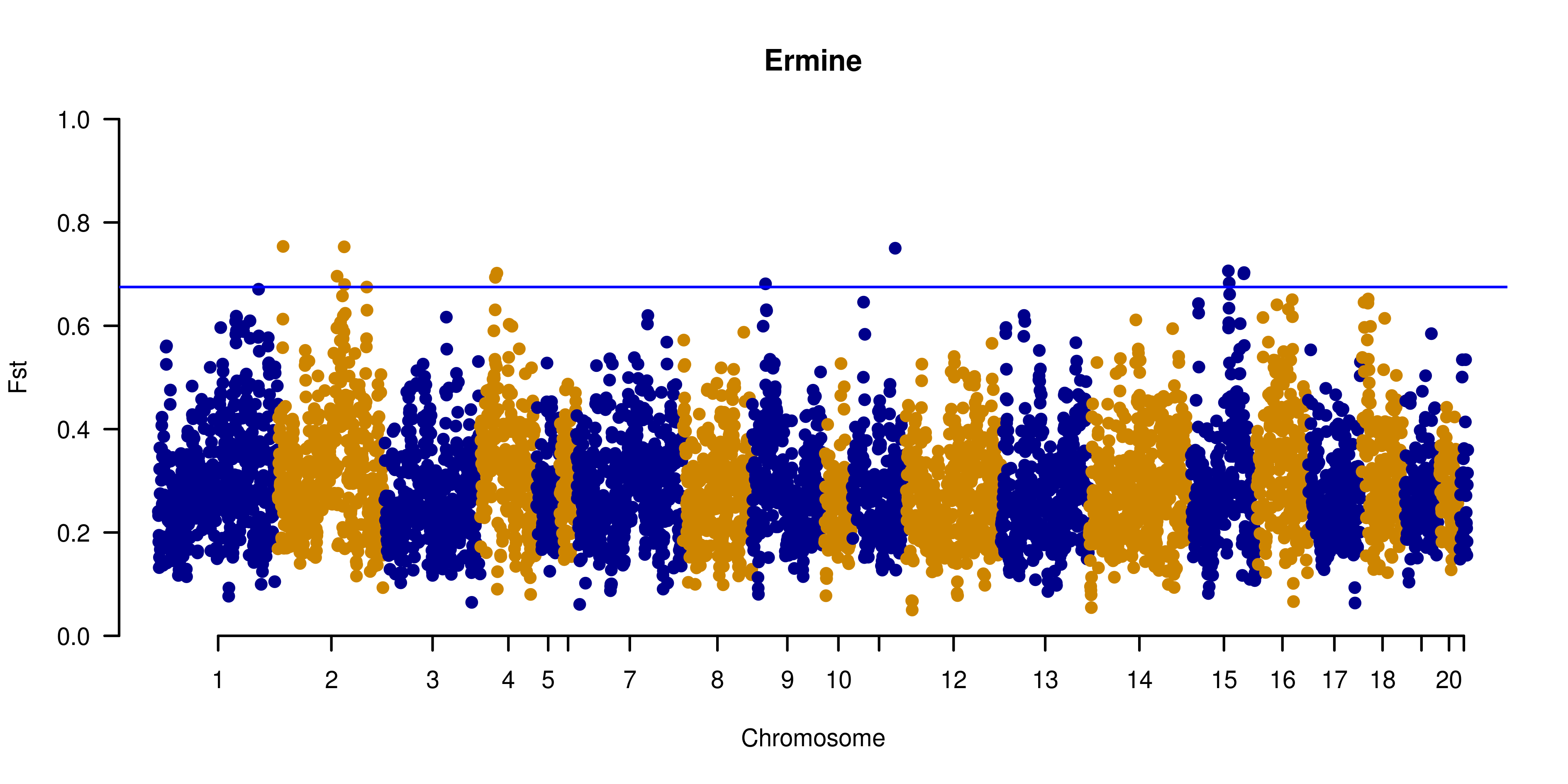
**


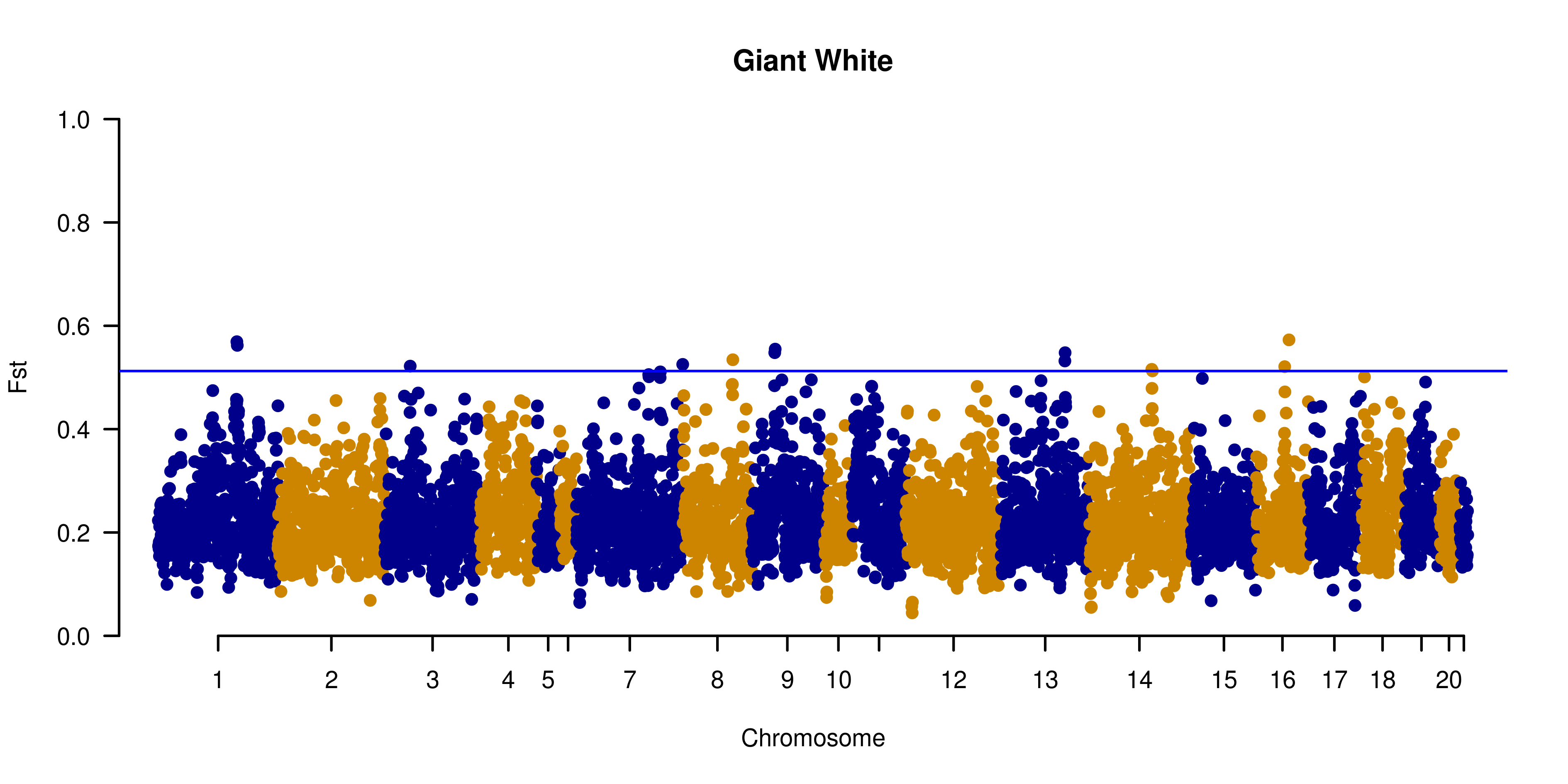


**
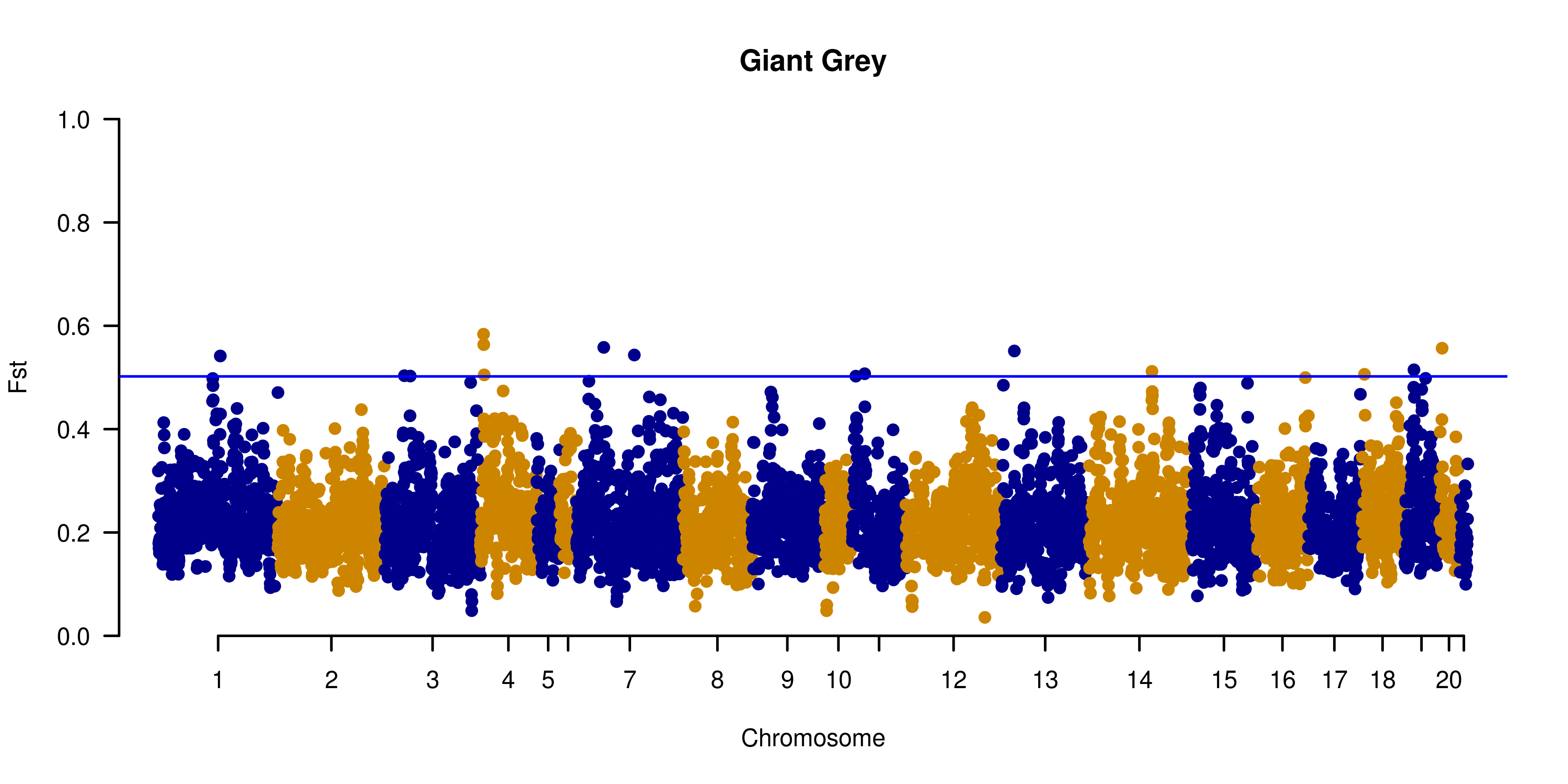
**

**
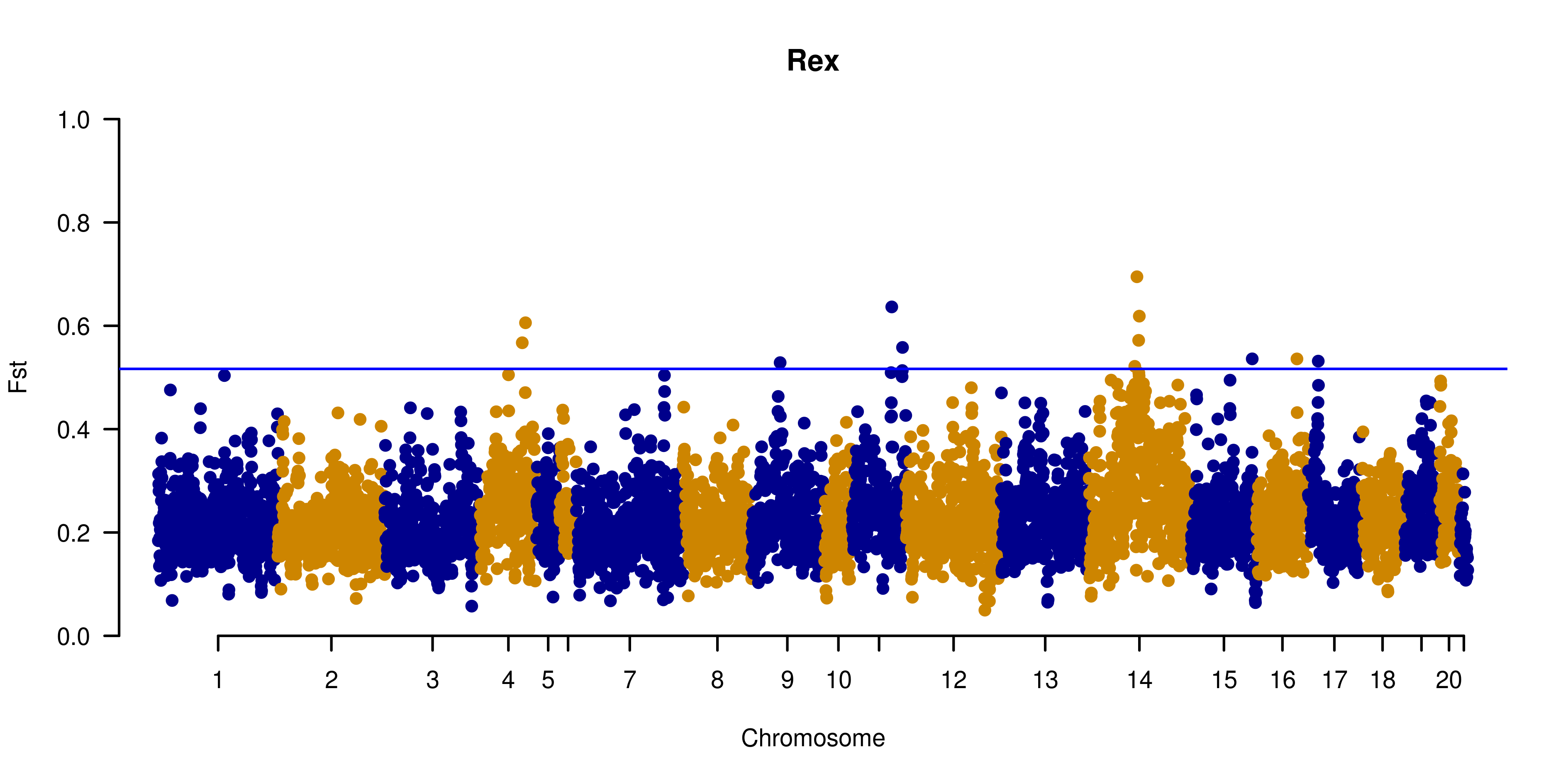

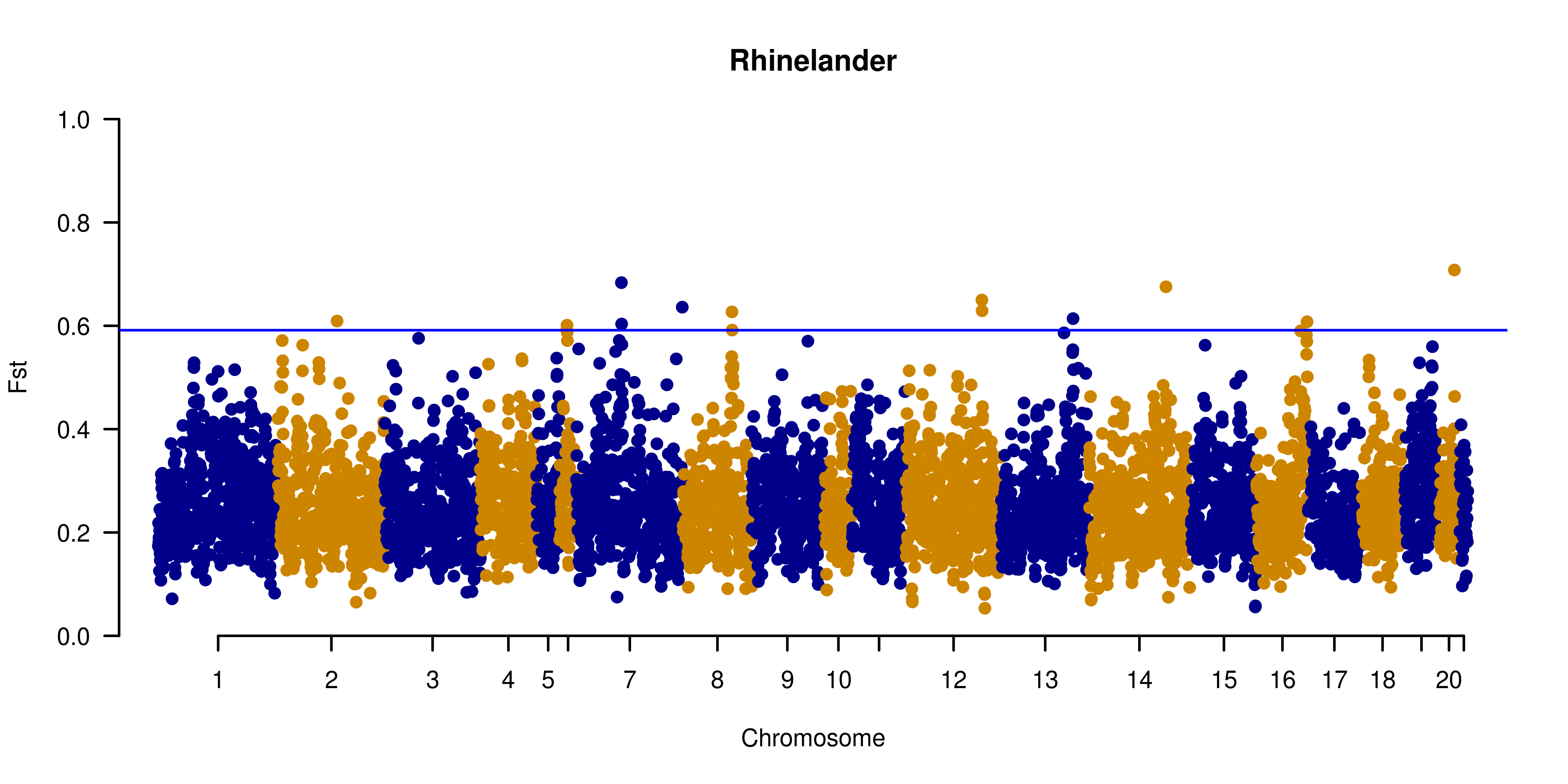

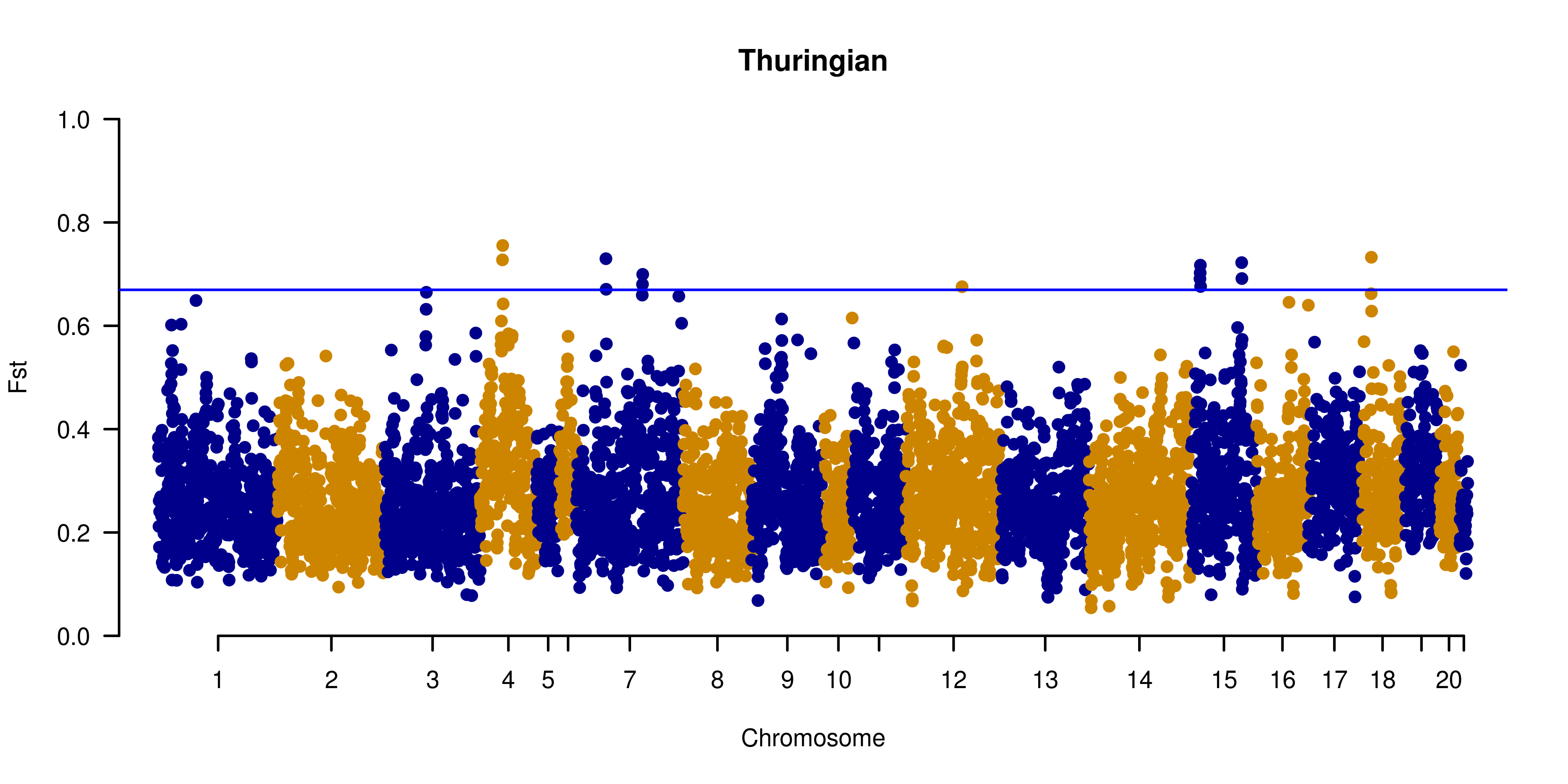
**
